# Microstructural alterations predict impaired bimanual control in Parkinson’s disease

**DOI:** 10.1093/braincomms/fcac137

**Published:** 2022-05-22

**Authors:** Philipp A. Loehrer, Immo Weber, Carina R. Oehrn, Felix S. Nettersheim, Haidar S. Dafsari, Susanne Knake, Marc Tittgemeyer, Lars Timmermann, Marcus Belke

**Affiliations:** Department of Neurology, Philipps-University Marburg, Marburg, Germany; Department of Neurology, Philipps-University Marburg, Marburg, Germany; Center for Mind, Brain and Behavior (CMBB), Philipps-University Marburg, Marburg, Germany; Department of Neurology, Philipps-University Marburg, Marburg, Germany; Center for Mind, Brain and Behavior (CMBB), Philipps-University Marburg, Marburg, Germany; Department of Cardiology, University Hospital Cologne, Cologne, Germany; Department of Cardiology, University Hospital Cologne, Cologne, Germany; Department of Neurology, University Hospital Cologne, Cologne, Germany; Department of Neurology, Philipps-University Marburg, Marburg, Germany; Center for Mind, Brain and Behavior (CMBB), Philipps-University Marburg, Marburg, Germany; Center for Personalized Translational Epilepsy Research (CePTER) Consortium, Frankfurt am Main, Germany; Max Planck Institute for Metabolism Research, Cologne, Germany; Excellence Cluster on Cellular Stress Responses in Aging-Associated Diseases (CECAD), Cologne, Germany; Department of Neurology, Philipps-University Marburg, Marburg, Germany; Center for Mind, Brain and Behavior (CMBB), Philipps-University Marburg, Marburg, Germany; Department of Neurology, Philipps-University Marburg, Marburg, Germany; Center for Personalized Translational Epilepsy Research (CePTER) Consortium, Frankfurt am Main, Germany

**Keywords:** bimanual coordination, Parkinson’s disease, diffusion imaging, NODDI, microstructure

## Abstract

Bimanual coordination is impaired in Parkinson’s disease affecting patients’ ability to perform activities of daily living and to maintain independence. Conveyance of information between cortical and subcortical areas is essential for bimanual coordination and relies on the integrity of cerebral microstructure. As pathological deposition of alpha-synuclein compromises microstructure in Parkinson’s disease, we investigated the relationship between microstructural integrity and bimanual coordination using diffusion-weighted MRI in 23 patients with Parkinson’s disease (mean age ± standard deviation: 56.0 ± 6.45 years; 8 female) and 26 older adults (mean age ± standard deviation: 58.5 ± 5.52 years). Whole-brain analysis revealed specific microstructural alterations between patients and healthy controls matched for age, sex, handedness, and cognitive status congruent with the literature and known Parkinson’s disease pathology. A general linear model revealed distinct microstructural alterations associated with poor bimanual coordination in Parkinson’s disease, corrected for multiple comparisons using a permutation-based approach. Integrating known functional topography, we conclude that distinct changes in microstructure cause an impediment of structures involved in attention, working memory, executive function, motor planning, motor control, and visual processing contributing to impaired bimanual coordination in Parkinson’s disease.

## Teaser

Loehrer *et al*. investigate the relationship between microstructural integrity and bimanual coordination in Parkinson’s disease and healthy controls employing diffusion-weighted imaging. Whole-brain analysis revealed that alterations in structures involved in attention, working memory, executive function, motor planning, motor control and visual processing predicted impaired bimanual coordination in Parkinson’s disease.

## Graphical Abstract

**Figure:**
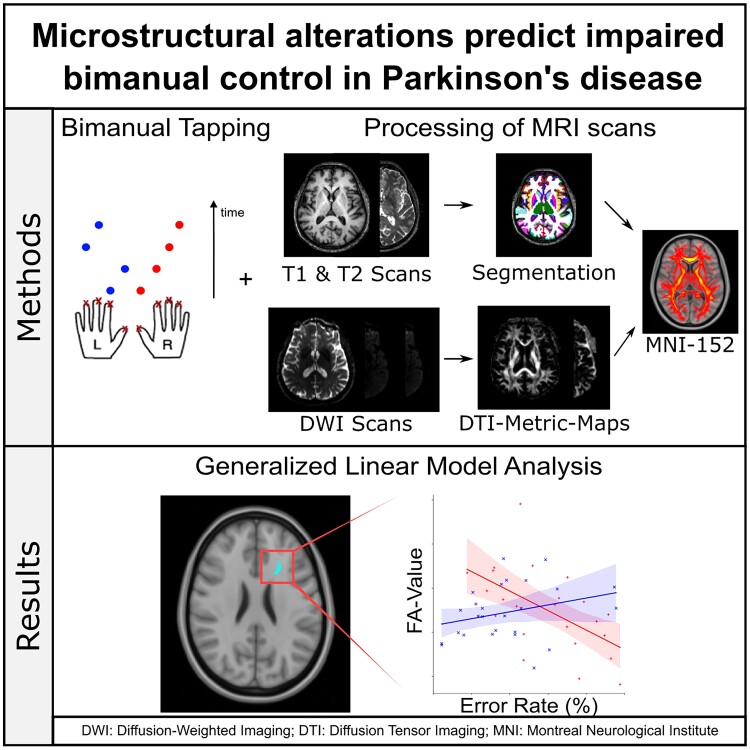

## Introduction

Bimanual coordination is essential for activities of daily living like eating with a knife and fork or buttoning a shirt. Patients with Parkinson’s disease commonly show difficulties in bimanual movement coordination affecting their ability to perform activities of daily living and to maintain independence.^1,2^ These difficulties particularly emerge during the performance of complex bimanual movements and can already be detected at the early stages of the disease.^3^

Bimanual movements are not mediated by a single dedicated area, but rather by a distributed network comprising distinct cortical and subcortical structures including the supplementary motor area (SMA), lateral premotor cortex, primary motor cortex (M1) and the basal ganglia.^4–7^ Information transfer between these distributed structures relies on the integrity of the connecting axons. Parkinson’s disease, however, is associated with a pathological deposition of alpha-synuclein in intraneuronal Lewy bodies within extended brain areas.^8^ This pathology is accompanied by axon demyelination as well as neuroglial damage, which represents specific microstructural alterations.^9^ Diffusion MRI (dMRI) non-invasively measures these changes *in vivo* by assessing the motion of water molecules within the tissue.^10^ In particular, diffusion tensor imaging (DTI) has been employed extensively to assess microstructural integrity in Parkinson’s disease.^9^ Recent advances in dMRI, namely the introduction and validation of Neurite Orientation Dispersion and Density Imaging (NODDI), have improved the capacity to characterize specific changes in tissue microstructure.^11,12^ NODDI provides information on the density and fanning of neurites, and the partial volume contamination from CSF and therefore increases specificity compared with conventional DTI measures.^11^ Previous studies employing DTI and NODDI in Parkinson’s disease have reported a complex distribution of microstructural alterations compared with healthy controls (HC) which could be linked to several motor and non-motor symptoms (for a review, see Zhang and Burock).^9^ Whether microstructural alterations underlie impaired bimanual coordination in Parkinson’s disease and the areas affected, however, remains to be addressed. We hypothesized that changes in the microstructure of distinct structures involved in working memory, executive function, motor planning and motor control contribute to impaired bimanual coordination in Parkinson’s disease. To assess the relationship between microstructure and bimanual coordination, we obtained dMRI scans from patients with Parkinson’s disease and HC, matched for age, sex, handedness and cognitive status, as well as performance metrics of complex bimanual finger movements. We employed DTI and NODDI to compare whole-brain microstructural alterations between patients and controls and relate alterations to behavioural parameters.

## Materials and methods

### Participants and behavioural data acquisition

Participants were recruited via the databases for recruiting patients with Parkinson’s disease and healthy participants of the Max Planck Institute for Metabolism Research (Department of Translational Neurocircuitry) and the University Hospital Cologne (Department of Neurology).

Thirty-three patients with Parkinson’s disease and 32 HC matched for age, sex, handedness and cognitive status participated in this study upon written informed consent. The clinical diagnosis of Parkinson’s disease was based on the UK Brain Bank Criteria. Patients were eligible to participate if they had normal MRI, no deep brain stimulation treatment, no concomitant neurological or psychiatric disease, were right-handed and aged under 65 years. The data sets of 23 patients with Parkinson’s disease and 26 HC (for sociodemographic data, see Table 1 and Supplementary Table 1) were included for further analysis (cf. below for exclusion criteria). Right-handedness was assessed with the Edinburgh Handedness Inventory.^13^ Participants did not play an instrument for >5 h/month and had normal neuropsychological test scores [Mini-Mental State Examination, DemTect (Dementia Detection Test) and Beck’s Depression Inventory; neuropsychological test scores are reported in Supplementary Tables 1 and 2].^14–16^ The local ethics committee approved the study (study number: 13-394) and experimental procedures were conducted in accordance with the Declaration of Helsinki.

**Table 1:** Sociodemographic information of patients with Parkinson’s disease

Age (years)	Gender	Hoehn and Yahr stage	UPDRS Part III OFF	UPDRS Part III ON	LEDD (mg)	Disease duration	Predominantly affected side
52	M	2	34	23	870	9	Right
43	M	2	15	10	500	3	Right
54	M	2	32	19	1298	7	Left
51	M	2.5	19	4	1195	4	Left
46	M	2	25	21	719,25	1	Left
64	F	2	22	4	562	8	Right
48	F	2	17	7	395	3	Right
63	F	3	29	15	1025	9	Right
64	F	2	20	7	320	6	Right
60	F	2	29	20	630	7	Right
58	M	2	31	11	710	7	Right
49	M	2	41	18	610	8	Right
57	F	2	22	10	300	2	Right
61	M	2	14	5	262	3	Right
64	M	2	10	4	420	6	Right
65	M	2	20	10	297	3	Right
50	M	2	34	26	100	6	Right
61	F	2	20	11	1110	6	Left
58	F	2	16	9	280	2	Left
56	M	2	17	10	715	4	Right
49	M	2	18	11	815	2	Left
58	M	2.5	19	6	240	1	Right
57	M	1	9	2	100	5	Right
Mean: 56.0	Ratio: F:M	Median: 2	Mean: 22.3	Mean: 11.4	Mean: 585.8	Mean: 4.9	Ratio: left:right
SD: 6.5	8:15	Range: 1–3	SD: 8.2	SD: 6.8	SD: 345.7	SD: 2.6	6:17

Sociodemographic information of Parkinson’s disease patients, severity of motor symptoms, medication requirements, disease duration since Parkinson’s disease diagnosis and side predominantly affected by Parkinson’s disease symptoms. F, female; LEDD, levodopa equivalent daily dose; M, male; SD, standard deviation; UPDRS, Unified Parkinson’s Disease Rating Scale.

**Figure 1:**
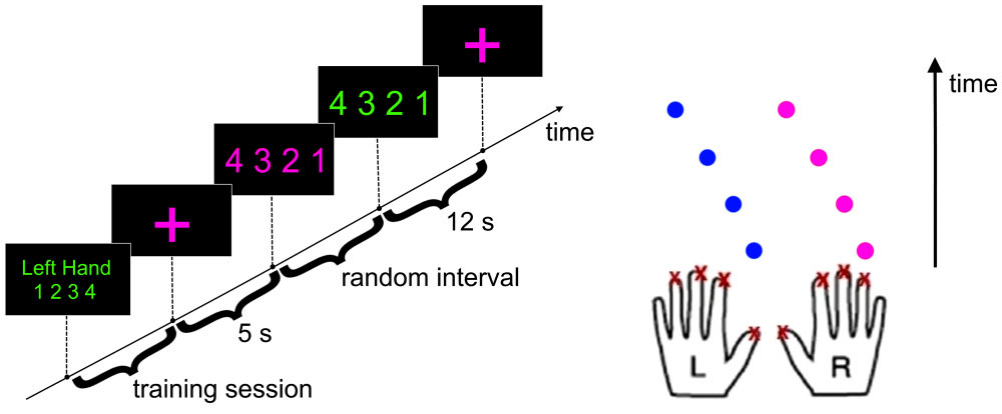
**Bimanual paradigm**. Instructions presented on a screen (left) and demanded button presses (right) in chronological order. Participants learned a sequence for their left hand (here: 1|2|3|4) and tapped a new sequence with their right hand. At the beginning of each trial, the upcoming sequence was presented in red (here: 4|3|2|1) and signalled the subject to prepare for the next trial. The switch from red numbers to green numbers served as ‘go’-signal, indicating to commence tapping. A red cross, followed by a short break of 5 s, marked the end of one trial. In this example, the first requested pair of button presses was the left thumb and right ring finger, followed by the left index and right middle finger. The third requested pair of button presses was the left middle and right index finger followed by the left ring finger and right thumb. Participants tapped in synchrony and were instructed to favour correct trial execution over speed. (Figure adapted from Loehrer *et al*.4; red was changed to magenta to provide a colour-blind-friendly figure.)

The behavioural task has been employed and validated in previous studies of our group.^1,4,7^ For a comprehensive description of the experimental conditions and paradigm, the reader is referred to Loehrer *et al*.^1,4^ In short, participants were seated in a comfortable chair in front of a computer screen. Their fingers were placed on a response pad (Cedrus, San Pedro, CA, USA) which consisted of eight buttons (four buttons for each hand). Each finger was allocated a number as well as a corresponding button (1 for left and right thumb; 2 for left and right index finger; 3 for left and right middle finger and 4 for left and right ring finger). Following a comprehensive introduction to the task, participants were instructed to memorize a sequence of four button presses for one hand (e.g. left hand 1|2|3|4). Subsequently, participants were able to practice the learned sequence and, at the end of each practice session, we assessed that tapping of both hands was strictly synchronous and a ceiling of errors had occurred. The learned sequence was executed with the respective hand while, simultaneously, the other hand tapped a different sequence (e.g. 4|3|2|1) that was presented on the screen (Fig. 1). To elaborate on differences in bimanual coordination between patients and HC, four sequences of two complexity levels, based on tapping direction and changes of tapping direction of a sequence, were defined. To avoid a learning bias and maintain comparability between groups, patients with Parkinson’s disease learned different sequences of the same complexity level for medication ON and OFF. These sequences were executed 24 times and participants were instructed to favour correct trial execution over speed. Learned sequences had to be executed with both hands, whereas the starting hand was counterbalanced across subjects. As differences in electrophysiology in medicated and unmedicated patients with Parkinson’s disease were assessed in another study,^1^ patients completed the behavioural paradigm in the medication OFF and ON (cf. Nettersheim *et al*.).^1^

Participants with >95% erroneous trials were excluded from further analysis (*n* = 4; three Parkinson’s disease patients, one HC). Furthermore, three patients with Parkinson’s disease and three HC discontinued behavioural analysis or withdrew from study participation due to various reasons (nausea after levodopa intake, severe sleep deprivation and scheduling difficulties) and were excluded. Three patients with Parkinson’s disease and one HC discontinued MRI acquisition due to claustrophobia, one patient with Parkinson’s disease did not receive a diffusion-weighted scan, and scans of one HC were affected by severe motion artefacts. These participants were excluded subsequently, leaving the data sets of 23 patients with Parkinson’s disease and 26 HC for final analysis.

### MRI data acquisition

Patients with Parkinson’s disease in the clinical ON and HC were scanned at the Max Planck Institute for Metabolism Research with a 3 T Trio scanner (Siemens, Erlangen, Germany). For each subject, a 3D T_1_-weighted Modified-Driven Equilibrium Fourier Transform sequence [MDEFT3D, field of view (FOV) = 256 × 256 × 128 voxel, voxel dimension: 1 × 1×1.25 mm, repetition-time (TR) = 1930 ms, echo-time (TE) = 5.8 ms, inversion time (TI) = 650 ms, flip-angle = 18°, bandwidth (BW) = 210 Hz/Pixel] was acquired. Furthermore, we obtained a 3D T2 (FOV = 240 × 256 × 176 voxel, voxel dimension 1 × 1×1 mm, TR = 3200 ms, TE = 458 ms, BW = 510 Hz/Pixel) and a diffusion scan (FOV = 128 × 128 × 90 voxel, voxel dimension 1.72 × 1.72 × 1.7 mm, TR = 11 200 ms, TE = 87 ms, BW = 1628 Hz/Pixel, six images with *b* = 0 s/mm² (b0) and 60 images with *b* = 1000 s/mm²). All images were investigated to be free of motion or ghosting, high frequency and/or wrap-around artefacts at the time of image acquisition.

### Image processing

The T1-MDEFT3D and T2 scans were transformed to a conform space with 1 mm isovoxel and an FOV of 256 × 256 × 256 voxel. MDEFT3D scans were analysed using the FreeSurfer-recon-all script, which was used with standard parameters, within FreeSurfer version 7.1. Processing included removal of non-brain tissue using a hybrid watershed/surface deformation procedure,^17^ automated Talairach transformations and segmentation of the subcortical white and deep grey matter volumetric structures.^18^ Furthermore, it included intensity normalization,^19^⁠ tessellation of the grey/white matter boundary, automated topology correction^20,21^⁠ and surface deformation following intensity gradients.^22^ Pial surfaces were improved using the different contrast in the T2 images.

DTI scans were preprocessed using FMRIB Software Library (FSL) 6.0.3. For motion and residual eddy current correction, each directional volume from the diffusion data set was registered and resampled to the first b0 volume.^23^ Subsequently, the diffusion tensor was calculated for each voxel in the volume using a linear regression fit to the diffusion signal. The first b0 image of each scan was linearly registered to the anatomical T1 space using a boundary-based method.^24^ Afterwards, a brainmask was calculated from the FreeSurfer segmentation including all white, cortical grey and subcortical grey matter. These masks were transformed to the diffusion space using the inverse of the previously calculated registration matrix. For evaluation of microstructural changes, fractional anisotropy (FA) was derived from the diffusion tensor.^25^ Additionally, the axial diffusivity (AD, *λ*1) and the radial diffusivity (RD, [*λ*2+*λ*3]/2) were calculated from the three eigenvalues (*λ*1, *λ*2, *λ*3) of the diffusion tensor.^26–28^ FA measures the directionality of random water motion and may be interpreted as a proxy of axonal integrity and the degree of axonal myelination.^9^ AD measures the extent of diffusion along the main axis and RD the extent of diffusion along the orthogonal axis. Decreased AD has been associated with axonal injury and increased RD with myelin degradation or thinning.^9,27^

Additionally, NODDI-DTI,^29^ a modification of NODDI,^11^ was used to calculate the intracellular volume fraction (ICVF) and orientation dispersion index (ODI) employing a python program based on DTI-NODDI.^30^ Here, ICVF represents neurite density and ODI the variability of neurite orientation.^11^ The b0 images were analysed to determine whether changes other than those in the tissue microstructure, e.g. white matter hyperintensities, contributed to the observed effects.

### Statistical analysis

Statistical analysis of behavioural data was performed using SPSS 22.0 (IBM, Armonk, NY, USA) for Windows 10. A non-Gaussian distribution of *error rates* was revealed by the Shapiro–Wilk test, thus data were square-root transformed. This resulted in normally distributed data which were used for further analysis. First, the variable e*rror rate* was entered into a repeated-measures ANOVA with the within-subject factors ‘complexity’ (Levels 1 and 2), ‘medication state’ (OFF versus ON) and ‘hand’ (learned sequence performed with left or right hand). Subsequently, we assessed differences in *error rates* between patients with Parkinson’s disease OFF medication and HC using a mixed-design ANOVA with the within-subjects factor ‘complexity’ and ‘hand’ as well as the between-subjects factor ‘group’ (Parkinson’s disease OFF versus Control). This analysis was repeated for patients with Parkinson’s disease ON medication (between-subject factor ‘group’: Parkinson’s disease ON versus Control). Homogeneity of variance and, when appropriate, sphericity were confirmed using Leven’s test and Mauchly’s test, respectively. Statistical significance was defined as *P* < 0.05. We pooled error rates for patients OFF and ON medication as well as complexity levels for further analysis as no differences in medication state were observed and to reduce data dimensionality.

Statistical analysis of image data was performed using FSL 6.0.3 and FreeSurfer Version 7.1. To calculate a voxelwise statistical analysis, the FA maps were first linearly and afterwards nonlinearly registered to the MNI152 space.^31,32^ The resulting warpfields were used to transform the FA, AD, RD, ICVF and ODI maps to the standard space. Subsequently, the brainmask was transformed using the same warpfields. The FA, AD, RD, ICVF and ODI maps were masked by these masks to exclude all voxels containing non-brain tissue and CSF and voxelwise statistics were carried out for the whole brain. Only voxels of brain tissue existing in every subject were included in the analysis.

Voxelwise cross-subject statistics were carried out as described previously,^33^ employing the tool mri_glmfit and mri_glmfit-sim of the FreeSurfer package. In short, the intensity values of each voxel were fit into a generalized linear model. Here, the forward model is given by:

y=W×X×B+n

with *y* being input data, *W* a weighting matrix, *X* the design matrix, *B* regression parameters and *n* noise. During estimation, the forward model is inverted to solve for *B*. Subsequently, a contrast matrix *C* is applied to *B* and an *F*-ratio is calculated for the given contrast. *F* is then used to derive a *P*-value.^34^ The results were corrected for multiple comparisons by a permutation-based approach based on the AFNI null-z simulator.^35^ Here, 12 000 simulations were performed under the null hypothesis. Voxels with a significance of *P* < 0.01 were clustered and a clusterwise *P*-value (CWP) was calculated. We only report clusters with a CWP of <0.05, corrected for multiple comparisons by the permutation-based approach described above.

### Data availability

The data that support the findings of this study are available on request from the corresponding author (P.A.L.). The data are not publicly available due to privacy or ethical restrictions.

### Code availability

All tools used for the analysis of MRI data are based on FreeSurfer Version 7.1 (http://surfer.nmr.mgh.harvard.edu/) and FSL 6.0.3 (http://www.fmrib.ox.ac.uk/fsl) packages, which are freely available. Scripts for automation were written in tcshell and parts of the statistics were written in Python using the packages numpy, pandas, seaborn, matplotlib, nibabel and scipy, which are also freely available. Python program code for the analysis of NODDI-DTI is available from https://github.com/dicemt/DTI-NODDI.

## Results

### Behavioural results

*Error rate* denotes the ratio of correct trials to overall trials. To evaluate differences in error rates between patients with Parkinson’s disease OFF and ON medication (*n* = 23), we conducted a repeated-measures ANOVA. Here, an effect for the within-subject factor ‘complexity’ [*F*(1,22) = 12.51, *P* = 0.002, Cohen’s *d*: 1.51] revealed that patients made more mistakes when they tapped a more complex sequence. Neither the factor ‘medication’ [ON versus OFF; *F*(1,22) = 0.21, *P* = 0.651, Cohen’s *d*: 0.2] nor ‘hand’ [learned sequence performed with left or right hand; *F*(1,22) = 0.004, *P* = 0.952, Cohen’s *d*: 0.03] differed between the respective variables. No further main effects or interactions were observed (all *P* > 0.066). To compare patients with Parkinson’s disease OFF medication (*n* = 23) and HC (*n* = 26), we employed a mixed-design ANOVA, which revealed that patients with Parkinson’s disease made more mistakes than HC [*F*(1,47) = 8.782, *P* = 0.005, Cohen’s *d*: 0.86]. No further main effects or interactions were found (all *P* > 0.152). Similarly, we compared patients with Parkinson’s disease ON medication (*n* = 23) and HC (*n* = 26) using a mixed-design ANOVA. Patients made more mistakes compared with HC [*F*(1,47) = 12.186, *P* = 0.001, Cohen’s *d*: 1.02] and a main effect for the within-subject factor ‘complexity’ [*F*(1,47) = 25.216, *P* < 0.001, Cohen’s *d*: 1.46] was revealed. Here, participants made more mistakes when they tapped a more complex sequence. No further main effects or interactions were observed (all *P* > 0.110).

### Image results

To assess differences in microstructure between patients with Parkinson’s disease (*n* = 23) and HC (*n* = 26), we employed a generalized linear model (GLM). Here, ‘Negative Cluster’ denotes clusters with reduced DTI-metric-values in patients with Parkinson’s disease compared with HC, whereby ‘Positive Cluster’ denotes clusters with increased DTI-metric-values in patients with Parkinson’s disease. Furthermore, the relationship between microstructure and participants’ error rates was assessed using a GLM. Here, ‘Negative Cluster’ denotes clusters with a lower slope in patients with Parkinson’s disease compared with HC in the association of participants’ DTI-metric-values and error rates, i.e. lower metric-values predicted increased error rates in patients. ‘Positive Cluster’, on the other hand, denotes clusters with a higher slope in patients with Parkinson’s disease compared with HC, i.e. higher metric-values predicted increased error rates in patients.

Clusters with significant changes were mostly located either in white or grey matter. Occasionally, some voxels of a cluster were located in both the white and grey matter of the MNI-152 template. This can be explained by an inaccuracy in the registration of the DTI data onto the MNI 152 template and by errors due to the upsampling of the 1.72 × 1.72 × 1.7 mm voxel to 1 × 1×1 mm voxel.

### Alterations of fractional anisotropy in patients with Parkinson’s disease

Patients with Parkinson’s disease showed lower FA values in comparison to HC in two clusters. Cluster 1 comprised the left red nucleus, left nucleus reticularis polaris and left substantia nigra (SN) (CWP: 0.024). Cluster 2 included the red nucleus, nucleus reticularis polaris and SN of the right hemisphere (CWP: 0.031). Furthermore, patients with Parkinson’s disease had higher FA values in three clusters which included the left corticospinal tract (CST; Cluster 1, CWP: 0.003), right inferior fronto-occipital fasciculus (IFOF) and right inferior longitudinal fasciculus (ILF; Cluster 2, CWP: 0.009), as well as left hippocampus (cluster 3, CWP: 0.042; Supplementary Fig. 1 and Table 3).

### Alterations of diffusivity measures in patients with Parkinson’s disease

AD values were reduced in 15 clusters in patients with Parkinson’s disease comprising multiple subcortical, cortical and cerebellar structures. Particularly, lower AD values were observed for patients with Parkinson’s disease in left (negative Cluster 2, CWP: <0.001) and right putamen (negative Cluster 5, CWP: <0.001; Supplementary Fig. 2 and Table 4 for AD-cluster-characteristics and Supplementary Table 5 for RD-cluster-characteristics). Furthermore, one cluster with increased RD values was observed comprising left anterior thalamic radiation (AThR, positive Cluster 1, CWP: 0.006).

### Alterations of NODDI parameters in patients with Parkinson’s disease

Patients with Parkinson’s disease showed higher ICVF and ODI parameters in 9 and 12 clusters, respectively, comprising subcortical, cortical and cerebellar structures overlapping with clusters harbouring altered diffusivity metrics (Supplementary Tables 6 and 7 and Figs. 3 and 4).

### Interaction between fractional anisotropy and bimanual performance

**Figure 2:**
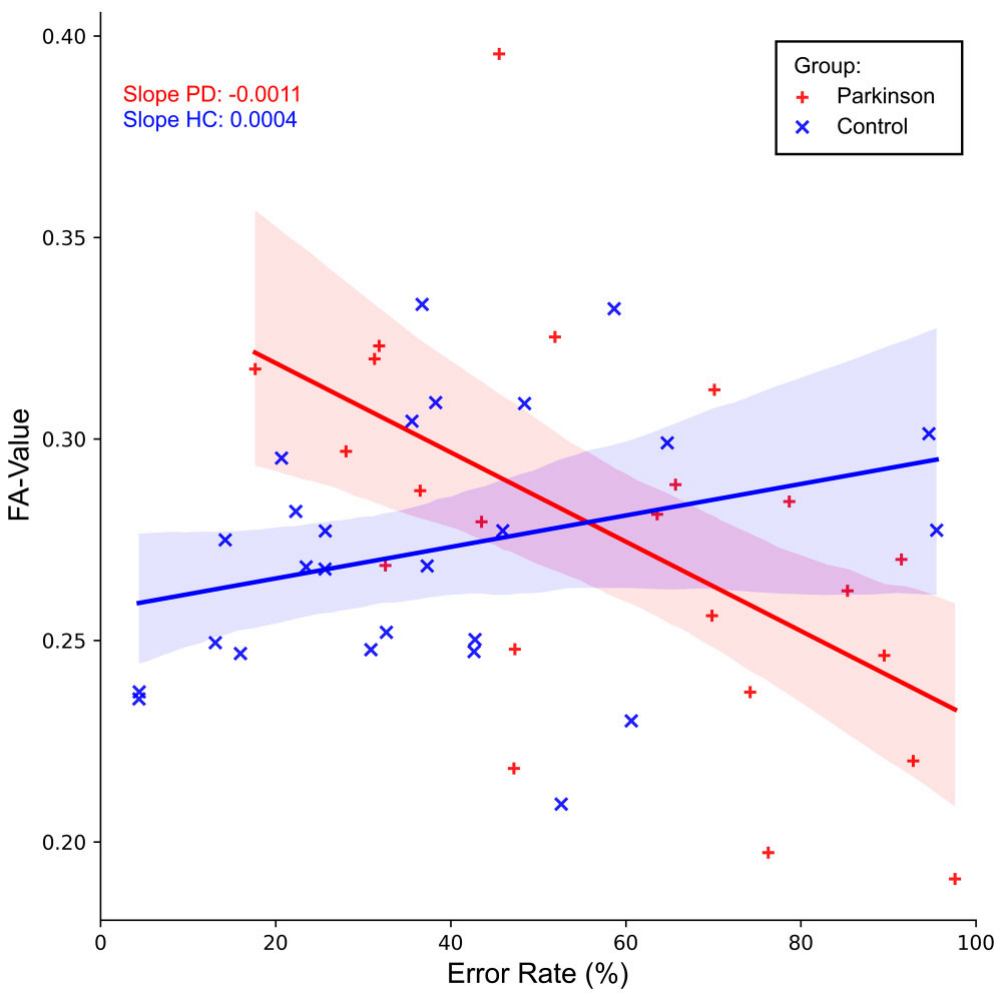
**Interaction between FA and bimanual performance in left AThR.** Association between white matter FA in left AThR and participants’ error rates as revealed by GLM. Lower FA values predicted higher error rates in patients with Parkinson’s disease, whereas this association was not present in HC.

**Figure 3:**
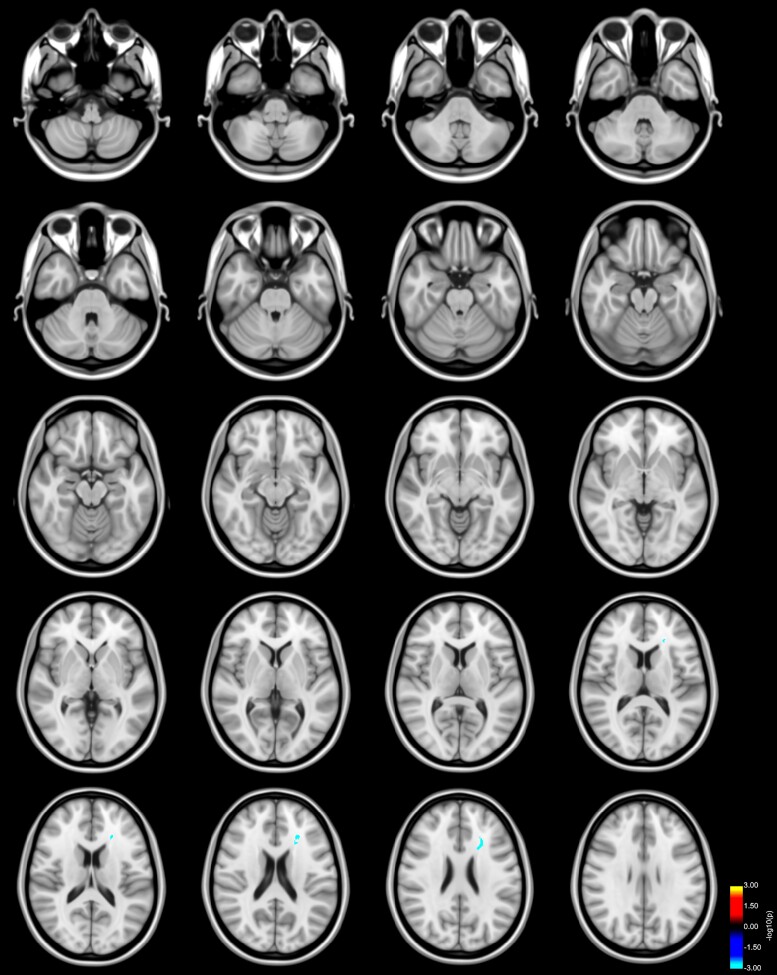
**Reduced FA in left AThR predicts bimanual performance in Parkinson’s disease**. Clusters with a lower slope (blue to light blue colour) in the analysis of patients with Parkinson’s disease FA values and error rates in comparison with the association of FA values and error rates of HC as revealed by the whole-brain analysis. *P*-values were corrected for multiple comparisons using a permutation-based approach. Results are displayed as the negative decadic logarithm of the *P*-value (*P* = 10^-*x*^).

The relationship between microstructure and participants’ error rates was assessed employing a GLM. Lower FA values in left AThR were related to increased *error rates* in patients with Parkinson’s disease (negative Cluster 1, CWP: < 0.001, Figs 2 and 3, Supplementary Table 8).

### Interaction between diffusivity measures and bimanual performance

Analysis of the interaction between participants’ AD values and *error rate* revealed 14 negative clusters in multiple subcortical, cortical and cerebellar structures. Particularly, negative clusters comprised major long association fibres such as the superior longitudinal fasciculus (SLF) in both hemispheres (negative Clusters 1 and 6, both CWP:  < 0.001), left ILF and left IFOF (both: negative Cluster 12, CWP: 0.015). Furthermore, negative clusters included bilateral pre- and post-central gyrus (negative Clusters 1 and 6, both CWP:  < 0.001) and the respective projection fibres, namely CST (right CST: negative Cluster 2, left CST: negative Cluster 6, both CWP:  < 0.001). Structures and association fibres of the limbic system, including the left hippocampus (negative Cluster 11, CWP: 0.012), left parahippocampus (negative Cluster 11, CWP: 0.012) and bilateral cingulum (CG; negative Clusters 3 and 5, both CWP:  < 0.001) as well as commissural fibres (forceps minor, e.g. negative cluster 3, CWP:  < 0.001) were inversely related to *error rates*.

**Figure 4:**
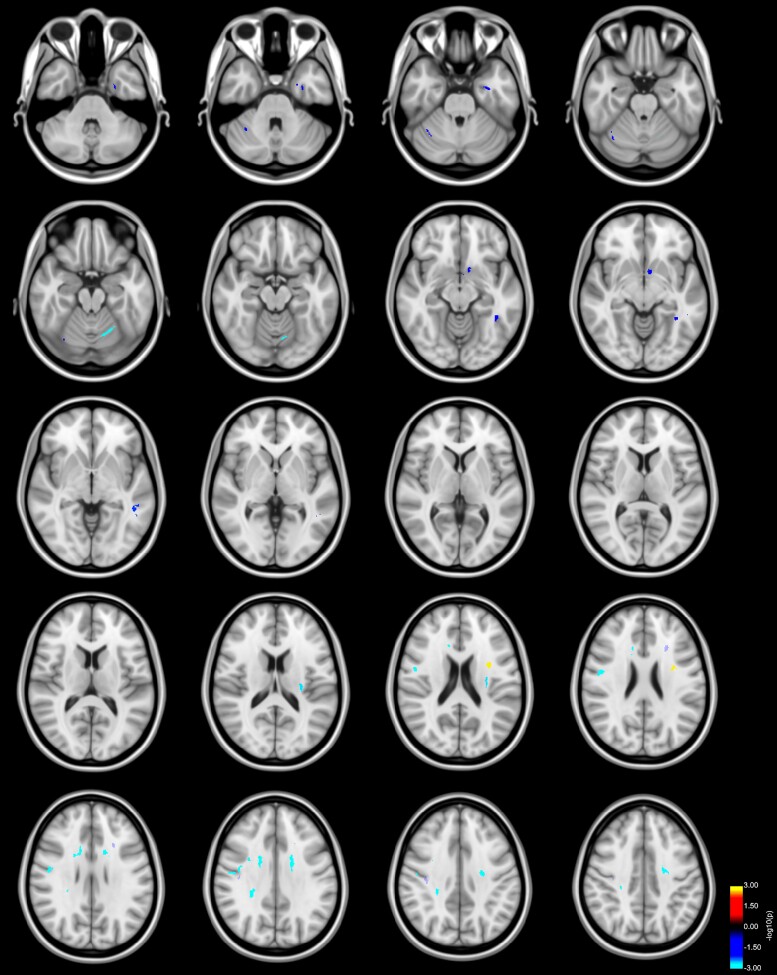
**Reduced axial and increased RD predicts bimanual performance in Parkinson’s disease.** Clusters with a lower (blue to light blue colour) or higher (red to yellow colour) slope in patients with Parkinson’s disease compared with HC in the association of participants’ AD values and error rates (only lower slopes depicted), as well as RD values and error rates (only higher slopes depicted) as revealed by the whole-brain analysis. *P*-values were corrected for multiple comparisons using a permutation-based approach. Results are displayed as the negative decadic logarithm of the *P*-value (*P* = 10^-*x*^).

Higher RD values in left SLF were related to increased *error rates* in patients with Parkinson’s disease (positive Cluster 1, CWP: 0.002; Fig. 4 and Supplementary Table 9 for AD values, Fig. 4 and Supplementary Table 10 for RD values).

### Interaction between NODDI parameters and bimanual performance

Analysis of interaction between participants’ ICVF values and *error rates* yielded positive clusters in the bilateral cerebellum (positive Cluster 1, CWP: 0.002; positive Cluster 3, CWP: 0.03) and left CG (positive Cluster 2, CWP: 0.01).

**Figure 5:**
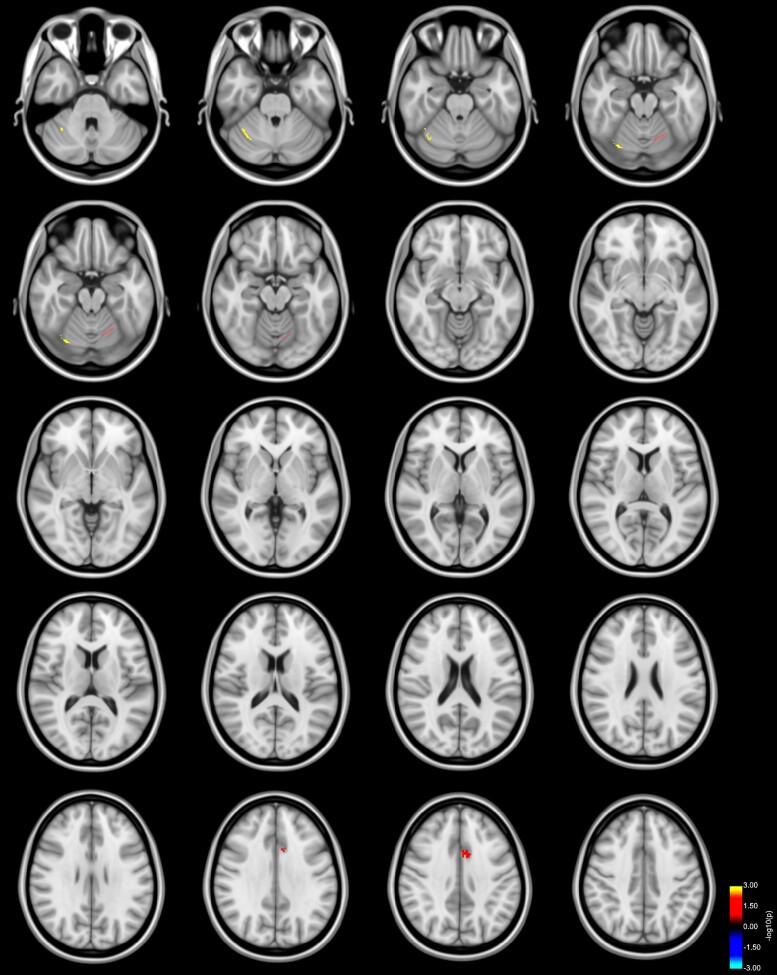
**Interaction between ICVF and bimanual performance**. Clusters with a lower (blue to light blue colour) or higher (red to yellow colour) slope in Parkinson’s disease patients compared with HC in the association of participants’ ICVF values and error rates as revealed by the whole-brain analysis. *P*-values were corrected for multiple comparisons using a permutation-based approach. Results are displayed as the negative decadic logarithm of the *P*-value (*P* = 10^-*x*^).

**Figure 6:**
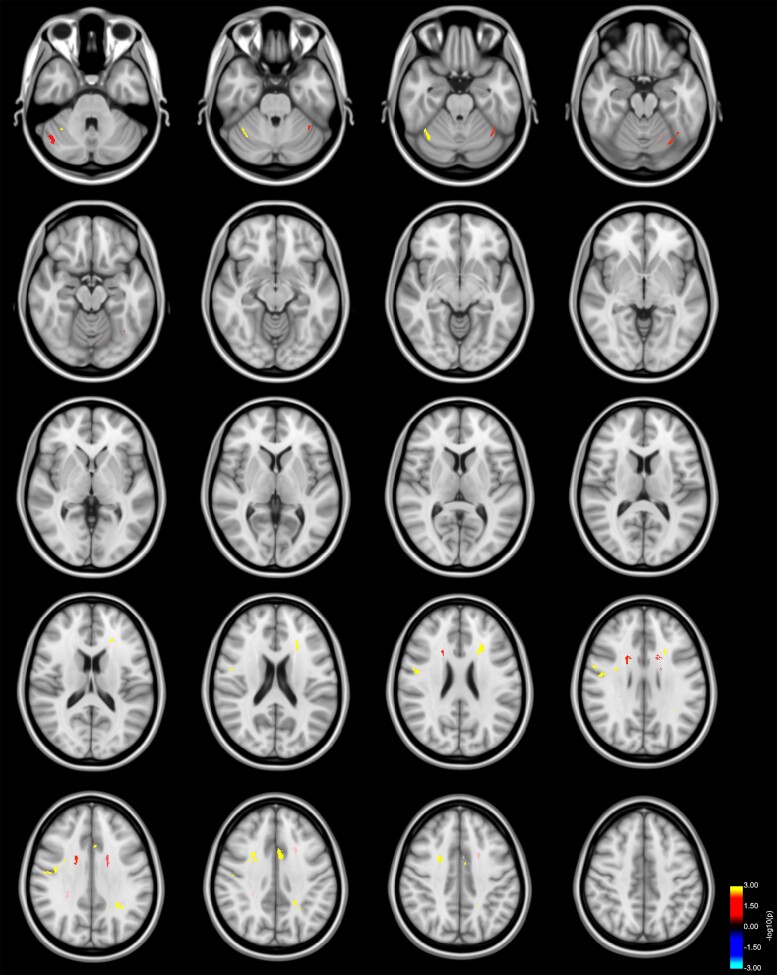
**Interaction between ODI and bimanual performance**. Clusters with a lower (blue to light blue colour) or higher (red to yellow colour) slope in patients with Parkinson’s disease compared with HC in the association of participants’ ODI values and error rates as revealed by the whole-brain analysis. *P*-values were corrected for multiple comparisons using a permutation-based approach. Results are displayed as the negative decadic logarithm of the *P*-value (*P* = 10^-*x*^).

Higher ODI values in patients with Parkinson’s disease were related to higher *error rates* in multiple subcortical, cortical and cerebellar structures overlapping with clusters harbouring altered diffusivity metrics (Fig. 5 and Supplementary Table 1^1^ for ICVF values, Fig. 6 and Supplementary Table 12 for ODI values).

## Discussion

In the present study, we investigated the relationship between microstructural integrity and bimanual motor control in Parkinson’s disease using diffusion-weighted MRIs of patients with Parkinson’s disease and HC matched for age, sex, handedness and cognitive status. We found spatially specific white matter abnormalities in projection and association fibres that predicted poor bimanual performance in Parkinson’s disease. Moreover, reduced microstructural integrity in patients with Parkinson’s disease within the left limbic system, commissural fibres, bilateral pre- and post-central gyrus and the cerebellum predicted impaired bimanual coordination.

### Alterations of microstructure in patients with Parkinson’s disease

Microstructural abnormalities in Parkinson’s disease have been studied extensively and a multifocal pattern of DTI changes has been described.^9^ These heterogeneous results reflect that Parkinson’s disease is a multisystem disorder and a clinically diverse disease on the one hand and the heterogeneous methodological approaches employed in the above-mentioned studies on the other. Consistent across the majority of studies investigating microstructural changes in Parkinson’s disease, however, is a reduced FA in SN.^9,36,37^ This finding is in accordance with the recognized pathophysiological hallmark of Parkinson’s disease, a degeneration of dopaminergic neurons in the SN pars compacts (SNc). In the present study, we replicated the finding of reduced FA values in bilateral SN in patients with Parkinson’s disease. It is, noteworthy, that most of the above-mentioned studies employed a region of interest (ROI) approach selecting the SN as seed ROI, whereas in our study, a whole-brain approach with a rigorous correction for multiple testing was implemented. Furthermore, our results revealed an increased FA in bilateral CST in accordance with previous findings.^9^ Although the physiological basis of increased FA is a matter of debate, a meta-analysis including 39 studies, which accounted for heterogeneities in methodology, also showed an increase in FA values within CST for Parkinson’s disease.^38^ These findings were interpreted as a possible compensatory mechanism secondary to a decreased input from the thalamus and striatum and could potentially reflect axonal sprouting.^38,39^ Furthermore, we found decreased AD, increased RD as well as increased ICVF and ODI in patients with Parkinson’s disease comprising multiple subcortical, cortical and cerebellar structures. Several of these alterations have been reported previously and linked to distinct motor and non-motor dysfunctions.^9^ Particularly, altered diffusivity measures in corpus callosum (CC), cingulate gyrus and IFOF correlated with motor dysfunction,^9^ whereas diffusion alterations in bilateral putamen were related to poor non-motor and motor experiences of daily living.^40^

Interpretation of increased AD and ICVF values as well as decreased RD values is a matter of ongoing debate. At present, neuropathologic validation for these changes is lacking and some authors argue that at least some of these changes might be due to methodological confounding factors.^9^ Thus, we refrain from interpreting these clusters.

Concluding, we found a distinct profile of microstructural changes in Parkinson’s disease replicating major findings consistent across the literature and in accordance with known Parkinson’s disease pathophysiology underlining the robustness of our findings.

### Interaction between microstructural changes and behavioural data in white matter tracts

Reduced FA and increased ODI in patients with Parkinson’s disease within the left AThR predicted poor bimanual performance. The AThR interconnects the anterior and midline nuclei of the thalamus with the frontal lobe, particularly the dorsolateral prefrontal cortex (DLPFC) and the premotor area.^41,42^ Besides the input from frontal and premotor areas, the respective nuclei receive input from gyrus cinguli, and the pallidum.^42^ Therefore, they are associated with the limbic system and thought to be involved in executive functions and planning of complex behaviour. The prefrontal cortex, on the other hand, is implicated in attention to action, online movement monitoring and working memory retrieval.^43,44^ Particularly, left DLPFC is thought to be involved in task setting and switching.^45^ As high levels of attention were required to accomplish our bimanual paradigm, reduced integrity in AThR is essential in this context. One possible explanation might be that microstructural alterations in this tract hamper the accurate relaying of motor and memory information between the pallidum and CG on the one hand and the prefrontal cortex on the other. In fact, it has consistently been shown that reduced FA values in AThR are associated with poor executive function in patients with bipolar disorder^46,47^ and movement dysfunction in Parkinson’s disease.^48,49^

The association of reduced AD and increased RD and ODI values in bilateral SLF with poor bimanual coordination suggests that altered information processing between frontal and parietal areas, connected via SLF,^50^ is a contributing factor to reduced bimanual coordination in Parkinson’s disease. A recent study suggests that the large fibre tract can be subdivided into four parts which facilitate distinct functions including visuospatial attention and motor control (dorsal SLF), auditory comprehension and articulatory processing (ventral SLF), language-related processing (posterior SLF) and language-related activities such as phonological processing (arcuate fasciculus, AF).^50^ Although the JHU-atlas employed in this study does not delineate the SLF into four parts, it comprises SLF and a temporal SLF (tSLF). tSLF includes the trajectories to the temporal lobe and corresponds to the posterior SLF and parts of the AF in Nakajima *et al*.^50,51^ Since altered diffusivity was observed in SLF only—and not tSLF—we conclude that primarily dorsal and ventral parts of the SLF were affected in our population. Both subdivisions are broadly involved in motor control, whereby a hemispheric predominance exists. Right-hemispheric SLF is involved in visuomotor processing and spatial working memory, whereas left-hemispheric SLF is involved in motor planning and recognition of postural changes.^50^ Furthermore, the dorsal SLF is a major part of the dorsal pathway of attention mediating visuospatial awareness and disruption of this pathway due to stroke or tumour results in contralateral spatial neglect.^50^ Based on these findings and the results of our analysis, we suggest that compromised microstructure of SLF contributes to impaired motor planning, visuomotor processing, spatial navigation and disruption of attentional circuits subsequently giving rise to constrained bimanual coordination.

The prediction of poor bimanual coordination by reduced AD values in ILF and IFOF suggests that altered processing of visual information is a contributing factor to poor bimanual coordination in Parkinson’s disease. The IFOF connects the frontal with the temporal and occipital lobes.^42^ Functions thought to be mediated by IFOF include attention and visual processing.^52^ The ILF, on the other hand, is involved in visual memory and object, face and place processing and is therefore particularly important for visually guided behaviour.^53^ Compromised microstructure might impede the conveyance of visual information between occipital, temporal and frontal lobes and therefore hamper neural integration of visual feedback. This is particularly important in the context of the task employed in this study, as instructions on the upcoming task were conveyed via visual information and no feedback on the tapping performance, except patients’ own visual feedback, was provided. In this regard, Ronsse *et al*.^^54^^ demonstrated that the performance of a bimanual task was superior when learning was accompanied by auditory feedback compared with visual feedback. Results of the complementing fMRI analysis suggested an increased reliance on feedback when learning with visual feedback, while a control mode less reliant on feedback seemed to develop when learning with auditory feedback.^54^ Integrating these findings with the finding of compromised microstructure in ILF and IFOF in patients with Parkinson’s disease, one could argue that patients could have pronounced difficulties when learning a bimanual motor task with visual feedback. This hypothesis requires further investigation. Furthermore, the role of compromised ILF and IFOF microstructure in Parkinson’s disease has been examined by a recent study linking the occurrence of visual hallucinations to reduced FA values in both tracts.^55^ Concluding, evidence exists that microstructural alterations in ILF and IFOF impairs visual information processing which contributes to the evolvement of specific clinical symptoms and can be associated with impaired behavioural measures. In the context of Parkinson’s disease, our results suggest that compromised microstructure in both fibre tracts can be a contributing factor to reduced bimanual coordination.

### Interaction between microstructural changes and behavioural data in grey matter

Microstructural alterations within grey matter can also be detected employing DTI. As cellular membranes of neurons are not aligned in one preferential direction, anisotropy is low.^56^ Thus, diffusivity measures such as AD and RD as well as NODDI parameters are appropriate metrics to investigate compromised microstructure due to a breakdown of cellular barriers resulting in increased diffusivity.^56^ In the present study, altered microstructure of grey matter predicted poor bimanual coordination of patients with Parkinson’s disease in bilateral pre- and post-central gyrus, left hippocampus and bilateral cerebellum. Furthermore, alterations in bilateral CST and CG are discussed here due to their close functional relation to pre- and post-central gyrus and hippocampus, respectively.

Reduced AD and increased ODI within and in close proximity to bilateral pre- and post-central gyrus and CST was associated with increased *error rates*. These findings indicate that microstructure within the major effectors of motor control is compromised in Parkinson’s disease which might contribute to poor bimanual coordination. Previous studies have shown congruent results and related alterations in these areas to overall motor dysfunction as measured by the UPDRS part III (Unified Parkinson’s Disease Rating Scale).^9,57^ Changes in microstructure in the post-central gyrus, in the proximity of the somatosensory cortex, indicate difficulties in integrating sensory information in Parkinson’s disease. Zhan *et al*.^^57^^ have previously described microstructural alterations in the post-central gyrus in patients with Parkinson’s disease and suggested that these changes are associated with sensory response abnormalities. Acknowledging the important role of sensory feedback in motor planning and execution,^58^ we speculate that altered microstructure in post-central gyrus affects sensory feedback processing and subsequently contributes to impaired bimanual control.

The fact that reduced AD and increased ODI (left CG only) in the left hippocampus, parahippocampus and bilateral CG predicted higher *error rates* indicates that impaired processing of memory information is a contributing factor to impaired bimanual coordination in Parkinson’s disease. The aforementioned structures are the key areas involved in the registration, storage and retrieval of memory information and thus essential for memory processing.^42^ In Parkinson’s disease, compromised microstructure in these areas has been associated with reduced scores in cognitive assessments,^59^ impaired visuospatial memory,^60^ dementia^61^ and visual hallucinations.^60^ Although participants in this study showed normal neuropsychological test scores and did not report visual hallucinations, even subtle changes in the aforementioned areas seem to contribute to impaired bimanual coordination.

Several changes in microstructure between HC and patients with Parkinson’s disease were observed in the cerebellum. An association with reduced bimanual coordination, however, was revealed in bilateral Lobule VI only. The cerebellum can be divided into three functional subdivisions comprising: (i) motor, (ii) attentional/executive and (iii) default-mode processing.^62^ According to this classification, Lobule VI is implicated in motor and particularly attentional and executive function.^62^ In this regard, Guell *et al*.^^63^^ could demonstrate that Lobule VI activation was present during finger tapping/toe grasping movements and 2-back working memory conditions. In the present study, participants recalled and tapped the sequence learned at the beginning of a session for one hand while a new sequence was tapped using the other hand. Therefore, utilizing working memory was vital in our paradigm. Hence, our results suggest that altered microstructure in Lobule VI in Parkinson’s disease impairs motor and working memory processing which might contribute to poor bimanual coordination.

### Limitations

Several limitations pertaining to our study have to be addressed. First, interpretation of DTI-derived indices in areas of low anisotropy (i.e. areas of complex axonal or dendritic architecture) is difficult. Second, although histopathological validation of NODDI exists and its use has been validated in Parkinson’s disease, no studies validating the neurite morphology revealed by NODDI in post-mortem brain tissue of patients with Parkinson’s disease exist. Third, we interpret our results in the light of the functional role of a respective structure described in the literature. This represents a limitation, as we did not employ tasks to test for attention, working memory or executive function separately. Testing for these functions, however, would have been impractical. Fourth, it is difficult to co-register the neocortex of every subject to a common space due to the broad inter-individual variety of the neocortex. Therefore, voxels with non-brain tissue in at least one subject were excluded.

## Conclusion

We describe a spatially distinct profile of microstructural alterations associated with poor bimanual coordination exceeding previously reported alterations related to overall movement dysfunction. Combining known functional topography with the present findings, structures important for attentional networks, working memory, executive function, overall motor control and planning as well as visual processing are affected and contribute to poor bimanual coordination in Parkinson’s disease.

## Supplementary Material

fcac137_Supplementary_Data

## Abbreviations

AD =: axial diffusivity
AThR =: anterior thalamic radiation
BW =: bandwidth
CG =: cingulum
CST =: corticospinal tract
CWP =: clusterwise *P*-value
DLPFC =: dorsolateral prefrontal cortex
dMRI =: diffusion magnetic resonance imaging
DTI =: diffusion tensor imaging
FA =: fractional anisotropy
FOV =: field of view
FSL =: FMRIB Software Library
GLM =: generalized linear model
HC =: healthy controls
ICVF =: intracellular volume fraction
IFOF =: inferior fronto-occipital fasciculus
ILF =: inferior longitudinal fasciculus
LEDD =: levodopa equivalent daily dose
lPM =: lateral premotor cortex
M1 =: primary motor cortex
MDEFT3D =: 3D T1-weighted modified-driven equilibrium Fourier transform sequence
MMSE =: Mini-Mental State Examination
MNI =: Montreal Neurological Institute
NODDI =: Neurite Orientation Dispersion and Density Imaging
ODI =: orientation dispersion index
RD =: radial diffusivity
ROI =: region of interest
SLF =: superior longitudinal fasciculus
SMA =: supplementary motor area
SN =: substantia nigra
TE =: echo-time
TI =: inversion time
TR =: repetition-time
UPDRS =: Unified Parkinson’s Disease Rating Scale
