# Supplementary material for: Microstructural alterations predict impaired bimanual control in Parkinson’s disease
Source: Brain Commun. 2022 May 22;4(3):fcac137. doi: 10.1093/braincomms/fcac137 (PMC9185383; doi:10.1093/braincomms/fcac137)
Supplement: fcac137_Supplementary_Data [file fcac137_supplementary_data.zip › Original Submission.pdf]

OXFORD UNIVERSITY PRESS | Brain Communications

Microstructural alterations predict impaired bimanual control in Parkinson's disease

Journal:	*Brain Communications*
Manuscript ID	BRAINCOM-2022-033
Manuscript Type:	Original Article
Date Submitted by the Author:	27-Jan-2022
Complete List of Authors:	Loehrer, Philipp; Philipps-Universität Marburg, Neurology<br>Weber, Immo; University Hospitals Giessen and Marburg, Neurology<br>Oehrn, Carina; Philipps-Universität Marburg, Department of Neurology; Philipps-Universität Marburg, Center for Mind, Brain and Behavior (CMBB); Philipps-Universität Marburg, Department of Neurology, Epilepsy Center Hessen<br>Nettersheim, Felix; University Hospital Cologne, Department of Cardiology<br>Dafsari, Haidar; University of Cologne, Faculty of Medicine and University Hospital Cologne, Department of Neurology<br>Knake, Susanne; Philipps-University Marburg, Dept. of Neurology<br>Tittgemeyer, Marc; Max-Planck Institute for Metabolism Research,<br>Timmermann, Lars; University Hospital Marburg Center of Neurology<br>Belke, Marcus; Philipps-Universität Marburg
Keywords:	Parkinson's disease, microstructure, Bimanual coordination, diffusion imaging, NODDI

SCHOLARONE™ Manuscripts

Microstructural alterations predict impaired bimanual control in Parkinson's disease

Philipp A. Loehrer[1*], Immo Weber[1,2], Carina R. Oehrn[1,2,3], Felix S. Nettersheim[3], Haidar S. Dafsari[4], Susanne Knake[1,2,5], Marc Tittgemeyer[6,7], Lars Timmermann[1,2], Marcus Belke[1,5]

[1]*Department of Neurology, Philipps-University Marburg, Marburg, Germany*

[2]*Center for Mind, Brain and Behavior (CMBB), Philipps-University Marburg, Marburg, Germany*

[3]*Department of Cardiology, University Hospital Cologne, Cologne, Germany*

[4]*Department of Neurology, University Hospital Cologne, Cologne, Germany*

[5]*Center for Personalized Translational Epilepsy Research (CePTER) Consortium*

[6]*Max Planck Institute for Metabolism Research, Cologne, Germany*

[7]*Excellence Cluster on Cellular Stress Responses in Aging-Associated Diseases (CECAD), Cologne, Germany*

*Corresponding author:

Philipp Loehrer, Department of Neurology, Philipps-University Marburg, Baldinger Str., 35043, Marburg, Germany, Tel: +49 6421 5866419, Email: Loehrer@staff.uni-marburg.de

**Number of words in the abstract:**	181/400
**Number of words in the manuscript:**	4652/6000
**Number of tables and figures:**	7/8
**Number of References:**	59
**Character count title:**	76/100
**Key words:**	5

Running title: Predicting bimanual control in Parkinson's disease

For Review Only

Running title: Predicting bimanual control in Parkinson's disease

Abstract

Bimanual coordination is impaired in Parkinson's disease (PD) affecting patients' ability to perform activities of daily living and to maintain independence. Conveyance of information between cortical and subcortical areas is essential for bimanual coordination and relies on the integrity of cerebral microstructure. As pathological deposition of alpha-synuclein compromises microstructure in Parkinson's disease, we investigated the relationship between microstructural integrity and bimanual coordination using diffusion-weighed MRI in 23 Parkinson's disease patients (mean age ± SD: 56.0 ± 6.45 years; 8 female) and 26 older adults (mean age ± SD: 58.5 ± 5.52 years). Whole-brain analysis revealed specific microstructural alterations between patients and healthy controls matched for age, sex, handedness, and cognitive status congruent with the literature and known Parkinson's disease-pathology. A general linear model revealed distinct microstructural alterations associated with poor bimanual coordination in Parkinson's disease, corrected for multiple comparisons using a permutation-based approach. Integrating known functional topography, we conclude that distinct changes in microstructure cause an impediment of structures involved in attention, working memory, executive function, motor planning, motor control, and visual processing contributing to impaired bimanual coordination in Parkinson's disease.

Key words: Bimanual coordination, Parkinson's disease, diffusion imaging, NODDI, microstructure

Abbreviations: AD = axial diffusivity; ANOVA = analysis of variance; AThR = anterior thalamic radiation; BW = bandwidth; BDI = Beck's Depression Inventory; CG = cingulum; CWP = clusterwise p-value; CST = corticospinal tract; DemTect = Dementia Detection Test; DLPFC = dorsolateral prefrontal cortex; dMRI = Diffusion Magnetic Resonance Imaging; DTI = Diffusion Tensor Imaging; EHI = Edinburgh Handedness Inventory; F = female; FA = fractional anisotropy; FOV = field of view; FSL = FMRIB Software Library; GLM = general

Running title: Predicting bimanual control in Parkinson's disease

linear model; HC = healthy controls; ICVF = intracellular volume fraction; IFOF = inferior fronto-occipital fasciculus; ILF = inferior longitudinal fasciculus; LEDD = levodopa equivalent daily dose; lPM = lateral premotor cortex; M = male; M1 = primary motor cortex; MDEFT3D = 3D T1-weighted Modified Driven Equilibrium Fourier Transform sequence; MMSE = Mini-Mental State Examination; MNI = Montreal Neurological Institute; NODDI = Neurite Orientation Dispersion and Density Imaging; ODI = orientation dispersion index; PD = Parkinson's disease; RD = radial diffusivity, ROI = region of interest; SD = standard deviation; SLF = superior longitudinal fasciculus; SMA = supplementary motor area; SN = substantia nigra; TE = echo-time; TI = inversion-time; TR = repetition-time UPDRS = Unified Parkinson's Disease Rating Scale

For Review Only

Running title: Predicting bimanual control in Parkinson's disease

1. Introduction

Bimanual coordination is essential for activities of daily living like eating with knife and fork or buttoning a shirt. Patients with Parkinson's disease (PD) commonly show difficulties in bimanual movement coordination affecting their ability to perform activities of daily living and to maintain independence.[1,2] These difficulties particularly emerge during the performance of complex bimanual movements and can already be detected at early stages of the disease.[3] Bimanual movements are not mediated by a single dedicated area, but rather a distributed network comprising distinct cortical and subcortical structures including the supplementary motor area (SMA), lateral premotor cortex (lPM), primary motor cortex (M1), and the basal ganglia.[4-7] Information transfer between these distributed structures relies on the integrity of the connecting axons. Parkinson's disease, however, is associated with a pathological deposition of alpha-synuclein in intraneuronal Lewy bodies within extended brain areas.[8] This pathology is accompanied by axon demyelination as well as neuroglial damage, which represent specific microstructural alterations.[9] Diffusion Magnetic Resonance Imaging (dMRI) non-invasively measures these changes in vivo by assessing the motion of water molecules within the tissue.[10] In particular, Diffusion Tensor Imaging (DTI) has been employed extensively to assess microstructural integrity in Parkinson's disease.[9] Recent advances in dMRI, namely the introduction and validation of Neurite Orientation Dispersion and Density Imaging (NODDI), have improved the capacity to characterise specific changes in tissue microstructure.[11,12] NODDI provides information on the density and fanning of neurites, and the partial volume contamination from cerebrospinal fluid and therefore increases specificity compared to conventional DTI measures.[11] Previous studies employing DTI and NODDI in Parkinson's disease have reported a complex distribution of microstructural alterations compared to healthy controls which could be linked to several motor and non-motor symptoms (for a review see Zhang and Burock).[9] Whether microstructural alterations underlie impaired bimanual

For Review Only

coordination in Parkinson's disease and the areas affected, however, remains to be addressed. We hypothesized that changes in microstructure of distinct structures involved in working memory, executive function, motor planning, and motor control contribute to impaired bimanual coordination in Parkinson's disease. To assess the relationship between microstructure and bimanual coordination, we obtained dMRI scans from Parkinson's disease patients and healthy controls (HC), matched for age, sex, handedness, and cognitive status, as well as performance metrics of complex bimanual finger movements. We employed DTI and NODDI to compare whole-brain microstructural alterations between patients and controls and relate alterations to behavioural parameters.

2. Materials and Methods

2.1 Participants and Behavioural Data Acquisition

Participants were recruited via the databases for recruiting Parkinson's disease patients and healthy participants of the Max-Planck-Institute for Metabolism Research (Department of Translational Neurocircuitry) and the University Hospital Cologne (Department of Neurology). 33 Parkinson's disease patients and 32 healthy controls (HC) matched for age, sex, handedness, and cognitive status participated in this study upon written informed consent. Clinical diagnosis of Parkinson's disease was based on the UK Brain Bank Criteria. Patients were eligible to participate if they had normal MR imaging, no deep brain stimulation treatment, no concomitant neurological or psychiatric disease, were right-handed, and aged under 65 years. The datasets of 23 Parkinson's disease patients and 26 HC (for sociodemographic data see Table 1 and Supplementary Table 1) were included for further analysis (cf. below for exclusion criteria). Right-handedness was assessed with the Edinburgh Handedness Inventory.[13] Participants did not play an instrument for more than five hours per month and had normal neuropsychological test scores (Mini-Mental State Examination, DemTect, and Beck's Depression Inventory; neuropsychological test scores are reported in Supplementary Table 1 and 2).[14-16] The local

For Review Only

ethics committee approved the study (study number: 13-394) and experimental procedures were conducted in accordance with the Declaration of Helsinki.

Sociodemographic information of Parkinson's disease patients

Age (years)	Gender	Hoehn & Yahr Stage	UPDRS Part III OFF	UPDRS Part III ON	LEDD (mg)	Disease duration	Predominantly affected side
52	M	2	34	23	870	9	right
43	M	2	15	10	500	3	right
54	M	2	32	19	1298	7	left
51	M	2.5	19	4	1195	4	left
46	M	2	25	21	719,25	1	left
64	F	2	22	4	562	8	right
48	F	2	17	7	395	3	right
63	F	3	29	15	1025	9	right
64	F	2	20	7	320	6	right
60	F	2	29	20	630	7	right
58	M	2	31	11	710	7	right
49	M	2	41	18	610	8	right
57	F	2	22	10	300	2	right
61	M	2	14	5	262	3	right
64	M	2	10	4	420	6	right
65	M	2	20	10	297	3	right
50	M	2	34	26	100	6	right
61	F	2	20	11	1110	6	left
58	F	2	16	9	280	2	left
56	M	2	17	10	715	4	right
49	M	2	18	11	815	2	left
58	M	2.5	19	6	240	1	right
57	M	1	9	2	100	5	right
Mean: 56.0 SD: 6.5	Ratio: F:M 8:15	Median: 2 Range: 1-3	Mean: 22.3 SD: 8.2	Mean: 11.4 SD: 6.8	Mean: 585.8 SD: 345.7	Mean: 4.9 SD: 2.6	Ratio: left:right 6:17

Table 1. Sociodemographic information of Parkinson's disease patients, severity of motor symptoms, medication requirements, disease duration since Parkinson's disease diagnosis, and side predominantly affected by Parkinson's disease symptoms. F = female; LEDD = levodopa equivalent daily dose; M = male; SD = standard deviation; UPDRS = Unified Parkinson's Disease Rating Scale

For Review Only

The behavioural task has been employed and validated in previous studies of our group.[1,4,7] For a comprehensive description of the experimental conditions and paradigm, the reader is referred to Loehrer et al.[1,4] In short, participants were seated in a comfortable chair in front of a computer screen. Their fingers were placed on a response pad (Cedrus, San Pedro, USA) which consisted of eight buttons (four buttons for each hand). Each finger was allocated a number as well as a corresponding button (1 for left and right thumb; 2 for left and right index finger; 3 for left and right middle finger and 4 for left and right ring finger). Following a comprehensive introduction to the task, participants were instructed to memorize a sequence of four button presses for one hand (e.g. left hand 1|2|3|4). Subsequently, participants were able to practice the learned sequence and, at the end of each practice session, we assessed that tapping of both hands was strictly synchronous and a ceiling of errors had occurred. The learned sequence was executed with the respective hand while, simultaneously, the other hand tapped a different sequence (e.g. 4|3|2|1) that was presented on the screen (Figure 1). To elaborate differences in bimanual coordination between patients and HC, four sequences of two complexity levels, based on tapping-direction and changes of tapping-direction of a sequence, were defined. To avoid a learning bias and maintain comparability between groups, Parkinson's disease patients learned different sequences of the same complexity level for medication ON and OFF. These sequences were executed 24 times and participants were instructed to favour correct trial execution over speed. Learned sequences had to be executed with both hands, whereas the starting hand was counter balanced across subjects. As differences in electrophysiology in medicated and unmedicated Parkinson's disease patients were assessed in another study,[1] patients completed the behavioural paradigm in the medication OFF and ON (cf. Nettersheim and Loehrer et al.).[1]

For Review Only

Running title: Predicting bimanual control in Parkinson's disease

Bimanual Paradigm

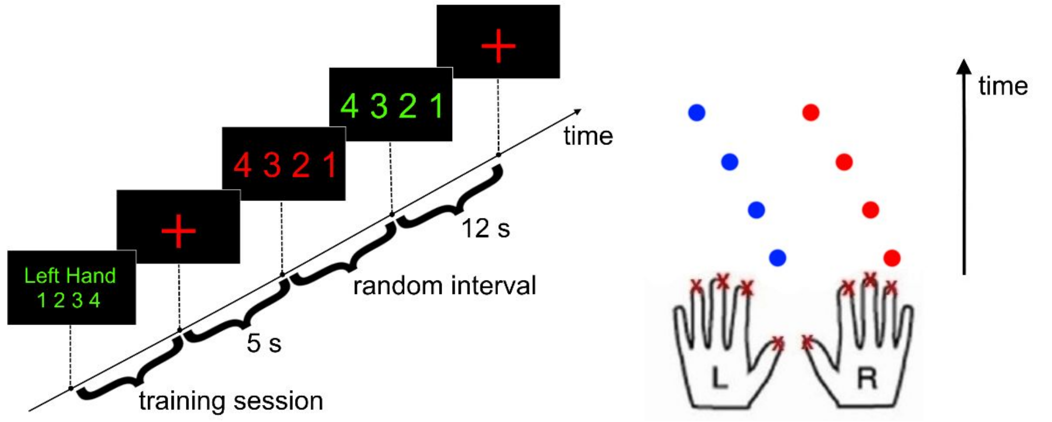

Figure 1. Instructions presented on a screen (left) and demanded button presses (right) in chronological order. Participants learned a sequence for their left hand (here: 1|2|3|4) and tapped a new sequence with their right hand. At the beginning of each trial, the upcoming sequence was presented in red (here: 4|3|2|1) and signalled the subject to prepare for the next trial. The switch from red numbers to green numbers served as "go"-signal, indicating to commence tapping. A red cross, followed by a short break of 5 seconds, marked the end of one trial. In this example, the first requested pair of button presses was left thumb and right ring finger, followed by left index and right middle finger. The third requested pair of button presses was left middle and right index finger followed by left ring finger and right thumb. Participants tapped in synchrony and were instructed to favour correct trial execution over speed. (Figure adapted from Loehrer et al., 2016).

For Review Only

Participants with more than 95% erroneous trials were excluded from further analysis (n = 4; three Parkinson's disease patients, one HC). Furthermore, three Parkinson's disease patients and three HC discontinued behavioural analysis or withdrew study participation due to various reasons (nausea after levodopa intake, severe sleep deprivation, and scheduling difficulties) and were excluded. Three Parkinson's disease patients and one HC discontinued MRI acquisition due to claustrophobia, one Parkinson's disease patient did not receive a diffusion weighed scan, and scans of one HC were affected by severe motion artefacts. These participants were excluded subsequently, leaving the datasets of 23 Parkinson's disease patients and 26 HC for final analysis.

2.2 MRI Data Acquisition

Parkinson's disease patients in the clinical ON and HC were scanned at the Max-Planck-Institute for Metabolism Research with a 3-Tesla Trio scanner (Siemens, Erlangen, Germany). For each subject a 3D T1-weighted Modified Driven Equilibrium Fourier Transform sequence (MDEFT3D, field of view (FOV) = 256x256x160 mm, voxel dimension: 1x1 mm, repetition-time (TR) = 1930 ms, echo-time (TE) = 5.8 ms, inversion time (TI) = 650 ms, flip-angle = 18°, bandwidth (BW) = 210 Hz/Pixel, slice-thickness = 1.25 mm) was acquired. Furthermore, we obtained a 3D T2 (FOV = 240x256x176 mm, voxel dimension 1x1 mm, TR = 3200 ms, TE = 458 ms, BW = 510 Hz/Pixel, slice-thickness = 1.0 mm) and a diffusion scan (FOV = 220x220x153 mm, voxel dimension 1.72x1.72 mm, TR = 11200 ms, TE = 87 ms, BW = 1628 Hz/Pixel, slice-thickness = 1.7 mm, six images with b = 0 s/mm² (b0) and 60 images with b = 1000 s/mm²). All images were investigated to be free of motion or ghosting, high frequency and/or wrap-around artefacts at the time of image acquisition.

For Review Only

2.3 Image Processing

The T1-MDEFT3D and T2 scans were transformed to a conform space with 1 mm isovoxel and a FOV of 256x256x256 voxel. MDEFT3D-scans were analysed using the FreeSurfer-recon-all script, which was used with standard parameters, within FreeSurfer version 7.1. Processing included removal of non-brain tissue using a hybrid watershed/surface deformation procedure,[17] automated Talairach transformation, and segmentation of the subcortical white and deep grey matter volumetric structures.[18] Furthermore, it included intensity normalization,[19] tessellation of the grey/white matter boundary, automated topology correction,[20,21] and surface deformation following intensity gradients.[22] Pial surfaces were improved using the different contrast in the T2 images.

DTI scans were preprocessed using FSL 6.0.3. For motion and residual eddy current correction, each directional volume from the diffusion dataset was registered and resampled to the first b0

volume.[23] Subsequently, the diffusion tensor was calculated for each voxel in the volume using a linear regression fit to the diffusion signal. The first b0 image of each scan was linearly registered to the anatomical T1 space using a boundary based method.[24] Afterwards a brainmask was calculated from the FreeSurfer segmentation including all white, cortical grey, and subcortical grey matter. These masks were transformed to the diffusion space using the inverse of the previously calculated registration matrix. For evaluation of microstructural changes, fractional anisotropy (FA) was derived from the diffusion tensor.[25] Additionally the axial diffusivity (AD, λ1) and the radial diffusivity (RD, [λ2+λ3]/2) were calculated from the three eigenvalues (λ1, λ2, λ3) of the diffusion tensor.[26-28] Fractional anisotropy (FA) measures the directionality of random water motion and may be interpreted as a proxy of axonal integrity and the degree of axonal myelination.[9] Axial diffusivity (AD) measures the extent of diffusion along the main axis and radial diffusivity (RD) the extent of diffusion along the orthogonal axis. Decreased AD has been associated with axonal injury and increased RD with myelin degradation or thinning.[9,29]

Additionally, NODDI-DTI,[30] a modification of NODDI,[11] was used to calculate the intracellular volume fraction (ICVF) and orientation dispersion index (ODI) employing a python-program based on DTI-NODDI.[31] Here, ICVF represents neurite density and ODI the variability of neurite orientation.[11] The b0 images were analysed to determine whether changes other than those in tissue microstructure, e.g. white-matter hyperintensities, contributed to the observed effects.

2.4 Statistical analysis

Statistical analysis of behavioural data was performed using SPSS 22.0 (IBM, Armonk, USA) for Windows 10. A non-Gaussian distribution of *error rates* was revealed by the Shapiro-Wilk test, thus square root transformed data were used for further analysis. First, the variable *error rate* was entered into a repeated measures analysis of variance (ANOVA) with the within-subject factors "complexity" (levels 1 and 2), "medication state" (OFF vs. ON), and "hand"

For Review Only

(learned sequence performed with left or right hand). Subsequently, we assessed differences in *error rates* between Parkinson's disease patients OFF medication and HC using a mixed design ANOVA with the within-subjects factor "complexity" and "hand" as well as the between-subjects factor "group" (PD OFF vs. Control). This analysis was repeated for Parkinson's disease patients ON medication (between-subject factor "group": PD ON vs. Control). Homogeneity of variance was assessed using Leven's Test and statistical significance was defined as $P < 0.05$. We pooled error rates for patients OFF and ON medication as well as complexity levels for further analysis as no differences in medication state were observed and to reduce data dimensionality.

Statistical analysis of image data was performed using FSL 6.0.3 and FreeSurfer Version 7.1. To calculate a voxelwise statistical analysis, the FA-maps were first linearly and afterwards nonlinearly registered to the MNI152 space.[32,33] The resulting warpfields were used to transform the FA-, AD-, RD-, ICVF- and ODI-maps to the standard space. Subsequently, the brainmask was transformed using the same warpfields. The FA-, AD-, RD-, ICVF- and ODI-maps were masked by these masks to exclude all voxels containing non-brain-tissue and cerebral spinal fluid and voxelwise statistics were carried out for the whole brain. Only voxels of brain tissue existing in every subject were included in the analysis.

Voxelwise cross-subject statistics were carried out as described previously,[34] employing FreeSurfer. The data was fit into a generalized linear model, and an unpaired t-test was performed. The results were corrected for multiple comparisons by a permutation-based approach based on the AFNI null-z simulator.[35] Here, 12.000 simulations were performed under the null hypothesis. Voxels with a significance of $p < .01$ were clustered and a clusterwise p-value was calculated. We only report clusters with a clusterwise p-Value of <.05, corrected for multiple comparisons by the permutation-based approach described above.

2.5 Data availability

The data that support the findings of this study are available on request from the corresponding author (PAL). The data are not publicly available due to privacy or ethical restrictions.

2.6 Code availability

All tools used for the analysis of MRI data are based on FreeSurfer Version 7.1 (http://surfer.nmr.mgh.harvard.edu/) and FSL 6.0.3 (http://www.fmrib.ox.ac.uk/fsl) packages, which are freely available. Scripts for automation were written in tcshell and parts of the statistics were written in Python using the packages numpy, pandas, seaborn, matplotlib, nibabel and scipy, which are also freely available. Python program code for the analysis of NODDI-DTI is available from https://github.com/dicemt/DTI-NODDI.

3. Results

3.1 Behavioural results

Error rate denotes the ratio of correct trials to overall trials. To evaluate differences in error rates between Parkinson's disease patients OFF and ON medication ($n = 23$), we conducted a repeated measures analysis of variance (ANOVA). Here, an effect for the within-subject factor "complexity" ($F(1,22) = 12.51$, $p = .002$, Cohen's d: 1.51) revealed that patients made more mistakes when they tapped a more complex sequence. Neither the factor "medication" (ON vs. OFF; $F(1,22) = .21$, $p = .651$, Cohen's d: .2) nor "hand" (learned sequence performed with left or right hand; $F(1,22) = .004$, $p = .952$, Cohen's d: .03) differed between the respective variables. No further main effects or interactions were observed (all $p > .066$). To compare Parkinson's disease patients OFF medication ($n = 23$) and HC ($n = 26$), we employed a mixed design ANOVA, which revealed that Parkinson's disease patients made more mistakes than HC ($F(1,47) = 8.782$, $p = .005$, Cohen's d: .86). No further main effects or interactions were found (all $p > .152$). Similarly, we compared Parkinson's disease patients ON medication ($n = 23$) and HC ($n = 26$) using a mixed design ANOVA. Patients made more mistakes compared to HC

For Review Only

(F(1,47) = 12.186, p = .001, Cohen's d: 1.02) and a main effect for the within-subject factor "complexity" (F(1,47) = 25.216, p < .001, Cohen's d: 1.46) was revealed. Here, participants made more mistakes when they tapped a more complex sequence. No further main effects or interactions were observed (all p > .110).

3.2 Image Results

3.2.1 Alterations of Fractional Anisotropy in Patients with Parkinson's disease

To assess differences in microstructure between Parkinson's disease patients and HC we employed a general linear model (GLM). Here, Parkinson's disease patients showed lower FA-values in comparison to HC in two clusters. Cluster 1 comprised left red nucleus, left nucleus reticularis polaris, and left substantia nigra (clusterwise p-value (CWP): .024). Cluster 2 included red nucleus, nucleus reticularis polaris, and substantia nigra of the right hemisphere (CWP: .031). Furthermore, Parkinson's disease patients had higher FA-values in three clusters which included left corticospinal tract (CST; cluster 1, CWP: .003), right inferior fronto-occipital fasciculus (IFOF) and right inferior longitudinal fasciculus (ILF; cluster 2, CWP: .009), as well as left hippocampus (cluster 3, CWP: .042; Supplementary Figure 1, Supplementary Table 3).

3.2.2 Alterations of Diffusivity Measures in Patients with Parkinson's disease

AD-values were reduced in 15 clusters in Parkinson's disease patients comprising multiple subcortical, cortical, and cerebellar structures. Particularly, lower AD-values were observed for Parkinson's disease patients in left (negative cluster 2, CWP: <.001) and right putamen (negative cluster 5, CWP: <.001; Supplementary Figure 2 and Supplementary Table 4 for AD-cluster-characteristics and Supplementary Table 5 for RD-cluster-characteristics). Furthermore, one cluster with increased RD-values was observed comprising left anterior thalamic radiation (AThR, positive cluster 1, CWP: .006).

For Review Only

3.2.3 Alterations of NODDI-Parameters in Patients with Parkinson's disease

Parkinson's disease patients showed higher ICVF- and ODI-parameters in nine and twelve clusters respectively comprising subcortical, cortical, and cerebellar structures overlapping with clusters harboring altered diffusivity metrics (Supplementary Tables 6 and 7, Supplementary Figure 3 and 4).

3.2.4 Interaction between Fractional Anisotropy and Bimanual Performance

The relationship between microstructure and participants' error rates was assessed employing a GLM. Lower FA-values in left anterior thalamic radiation (AThR) were related to increased *error rates* in Parkinson's disease patients (negative cluster 1, CWP: <.001, Fig. 2 and 3, Supplementary Table 8).

For Review Only

Running title: Predicting bimanual control in Parkinson's disease

Interaction between FA and bimanual performance in left anterior thalamic radiation

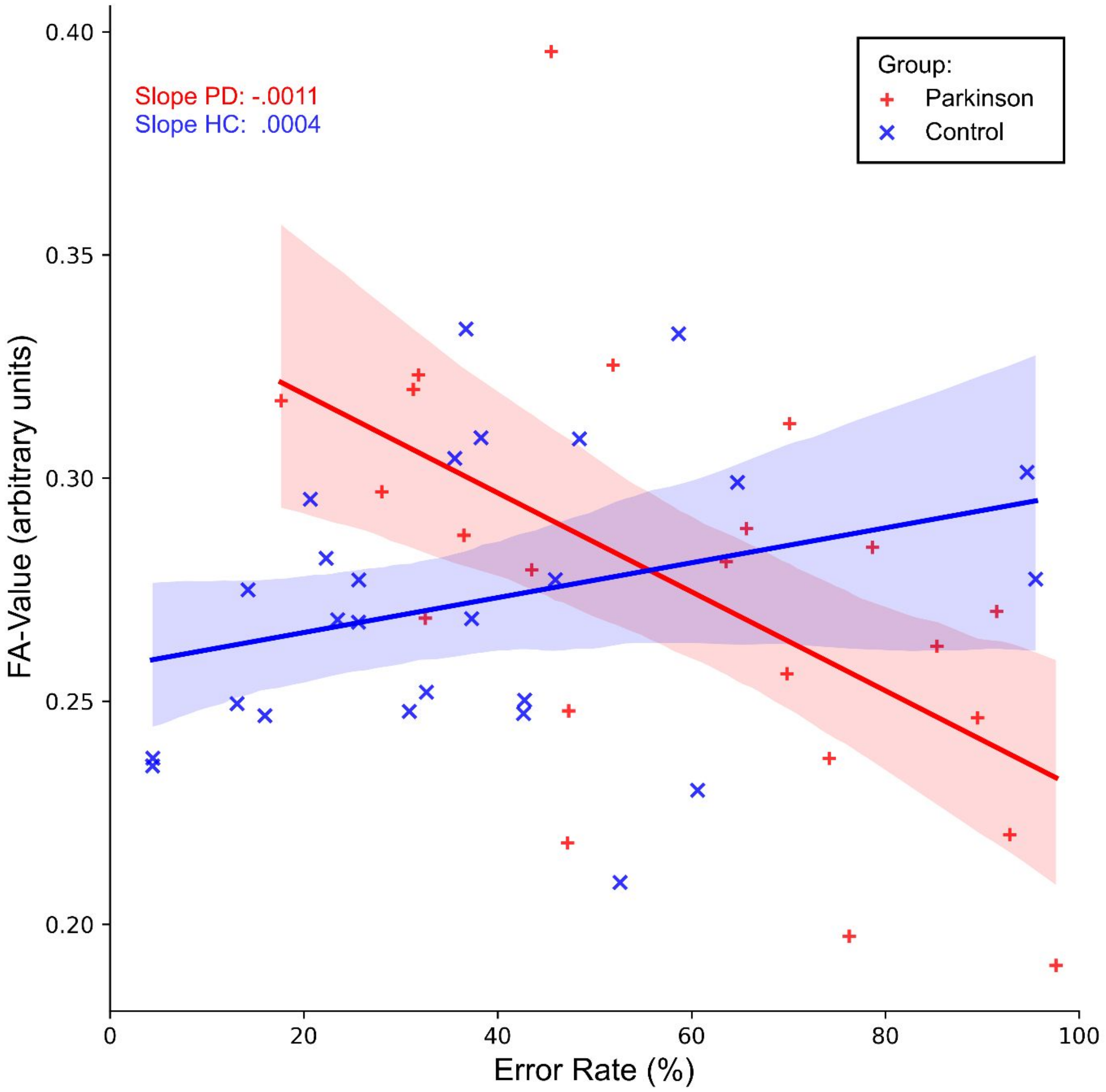

Figure 2. Association between white matter fractional anisotropy in left anterior thalamic radiation and participants' error rates as revealed by GLM. Lower FA-values predicted higher error rates in Parkinson's disease patients, whereas no significant association existed for healthy controls.

Reduced FA in left anterior thalamic radiation predicts bimanual performance in PD

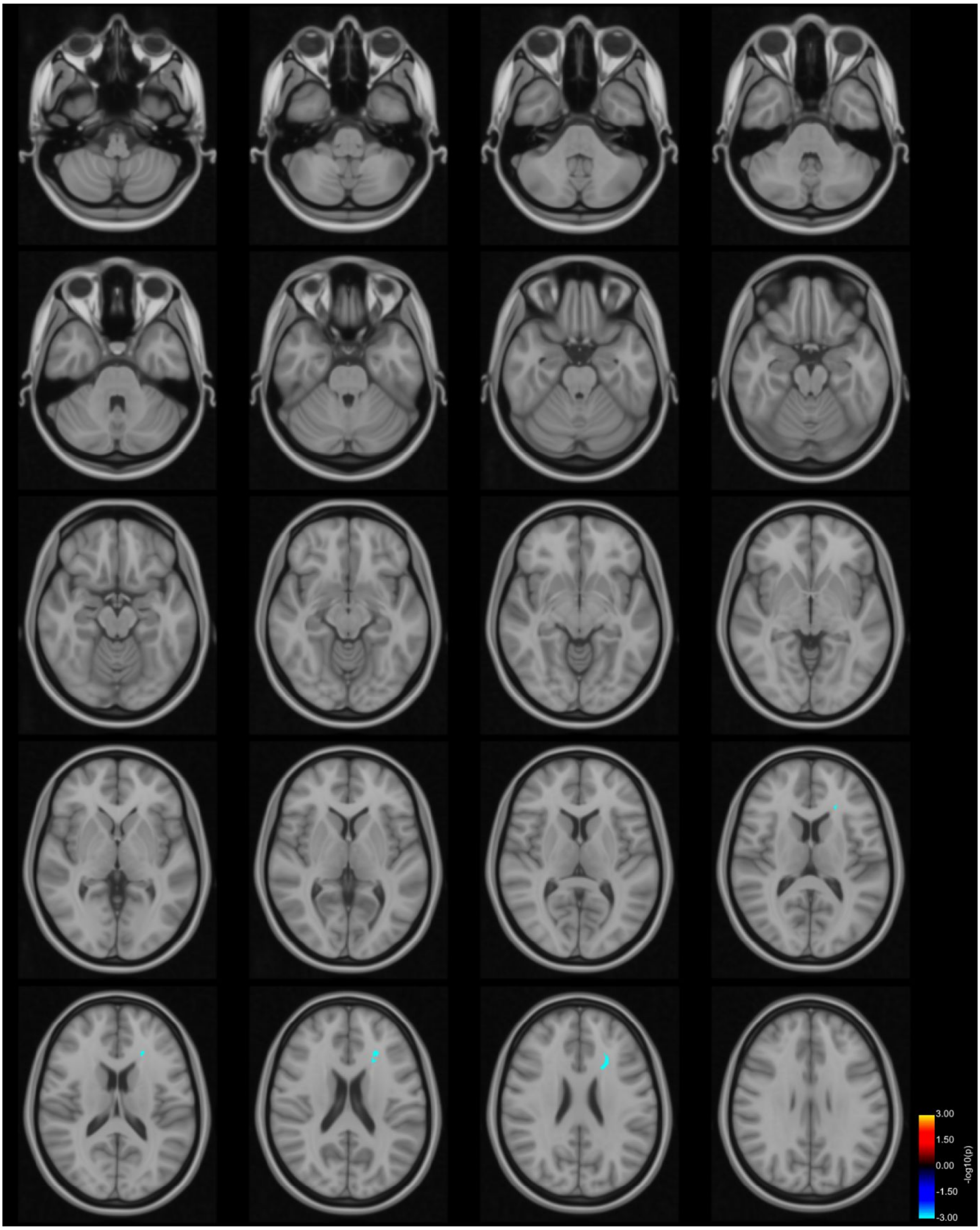

Figure 3. Clusters with a lower slope (blue to light blue colour) in the analysis of Parkinson's disease patients' FA-values and error rates in comparison to the association of FA-values and error rates of HC as revealed by the whole brain analysis. P-Values were corrected for multiple comparisons using a permutation-based approach. Results are displayed as the negative decadic logarithm of the p-value ($p=10^{-x}$).

3.2.5 Interaction between Diffusivity Measures and Bimanual Performance

Analysis of the interaction between participants' AD-values and *error rate* revealed 14 negative clusters in multiple subcortical, cortical, and cerebellar structures. Particularly, negative clusters comprised major long association fibers such as the superior longitudinal fasciculus (SLF) in both hemispheres (negative cluster 1 and 6, both CWP: <.001), left ILF, and left IFOF (both: negative cluster 12, CWP: .015). Furthermore, negative clusters included bilateral pre- and postcentral gyrus (negative cluster 1 and 6, both CWP: <.001) and the respective projection fibers, namely corticospinal tract (CST; right CST: negative cluster 2, left CST: negative cluster 6, both CWP: <.001). Structures and association fibers of the limbic system, including left hippocampus (negative cluster 11, CWP: .012), left parahippocampus (negative cluster 11, CWP: .012), and bilateral cingulum (CG; negative cluster 3 and 5, both CWP: <.001) as well as commissural fibers (forceps minor, e.g. negative cluster 3, CWP: <.001) were inversely related to *error rates*.

Higher RD-values in left SLF were related to increased *error rates* in Parkinson's disease patients (positive cluster 1, CWP: .002; Fig. 4 and Supplementary Table 9 for AD-values, Fig. 4 and Supplementary Table 10 for RD-values).

For Review Only

Reduced axial and increased radial diffusivity predicts bimanual performance in PD

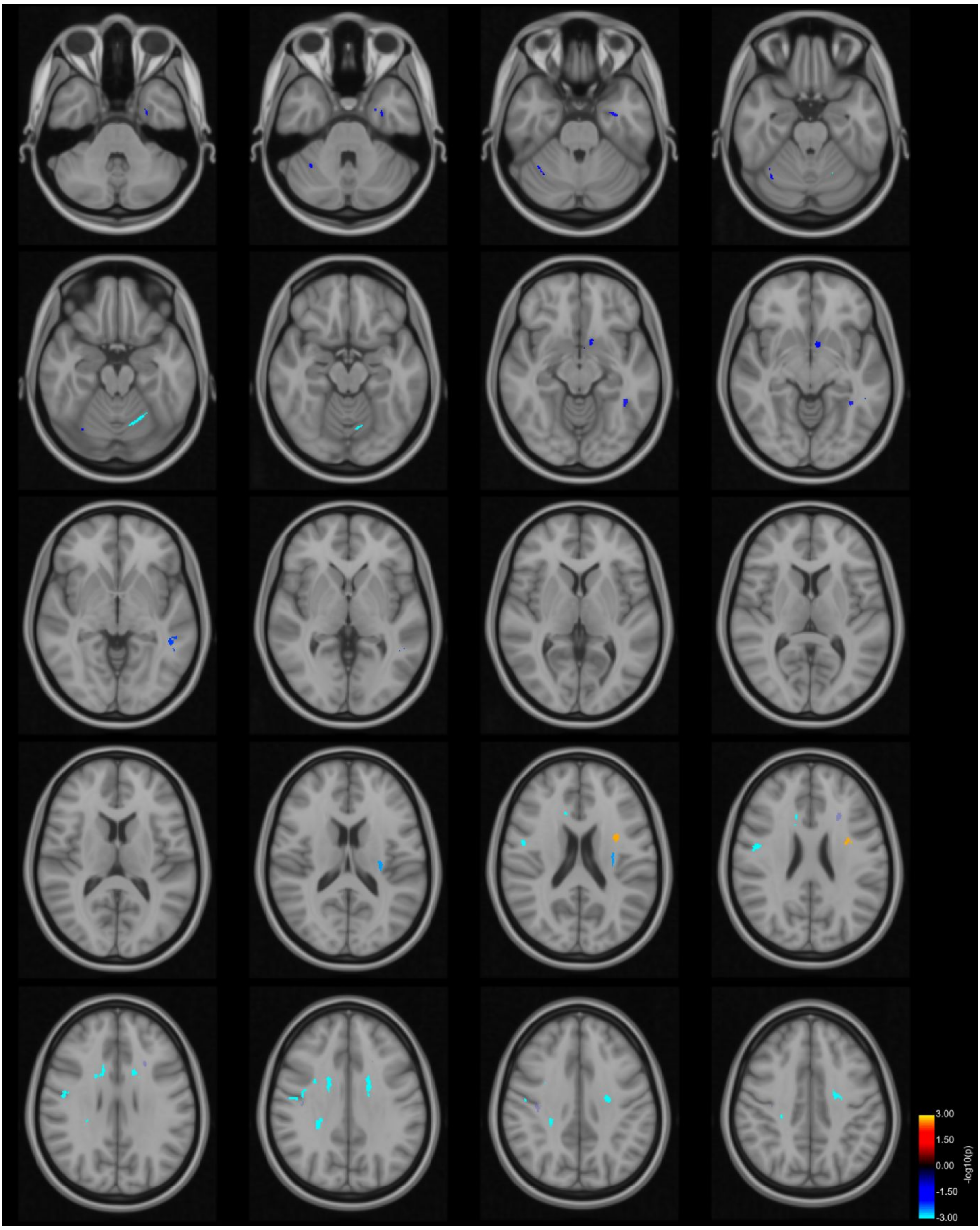

Figure 4. Clusters with a lower (blue to light blue colour) or higher (red to yellow colour) slope in Parkinson's disease patients compared to HC in the association of participants' AD-values and error rates (only lower slopes depicted), as well as RD-values and error rates (only higher slopes depicted) as revealed by the whole brain analysis. P-Values were corrected for multiple

comparisons using a permutation-based approach. Results are displayed as the negative decadic logarithm of the p-value ($p=10^{-x}$).

3.2.6 Interaction between NODDI-Parameters and Bimanual Performance

Analysis of interaction between participants' ICVF-values and *error rates* yielded positive clusters in bilateral cerebellum (positive cluster 1, CWP: .002; positive cluster 3, CWP: .03) and left cingulum (positive cluster 2, CWP: .01).

Higher ODI-values in Parkinson's disease-patients were related to higher *error rates* in multiple subcortical, cortical, and cerebellar structures overlapping with clusters harboring altered diffusivity metrics (Fig. 5 and Supplementary Table 11 for ICVF-values, Fig. 6 and Supplementary Table 12 for ODI-values).

For Review Only

Interaction between intracellular volume fraction and bimanual performance

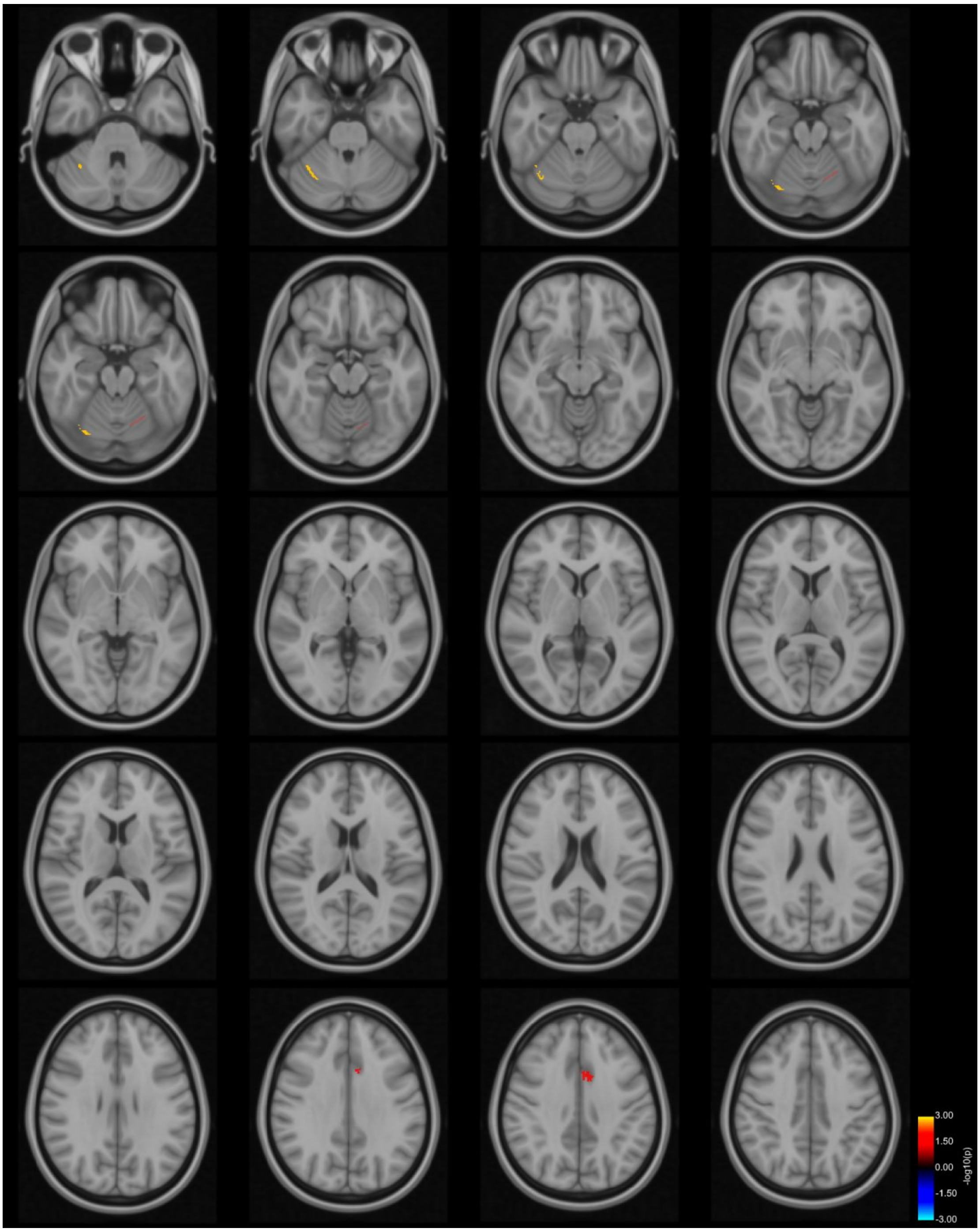

Figure 5. Clusters with a lower (blue to light blue colour) or higher (red to yellow colour) slope in Parkinson's disease patients compared to HC in the association of participants' ICVF-values and error rates as revealed by the whole brain analysis. P-Values were corrected for multiple comparisons using a permutation-based approach. Results are displayed as the negative decadic logarithm of the p-value ($p=10^{-x}$).

Interaction between orientation dispersion index and bimanual performance

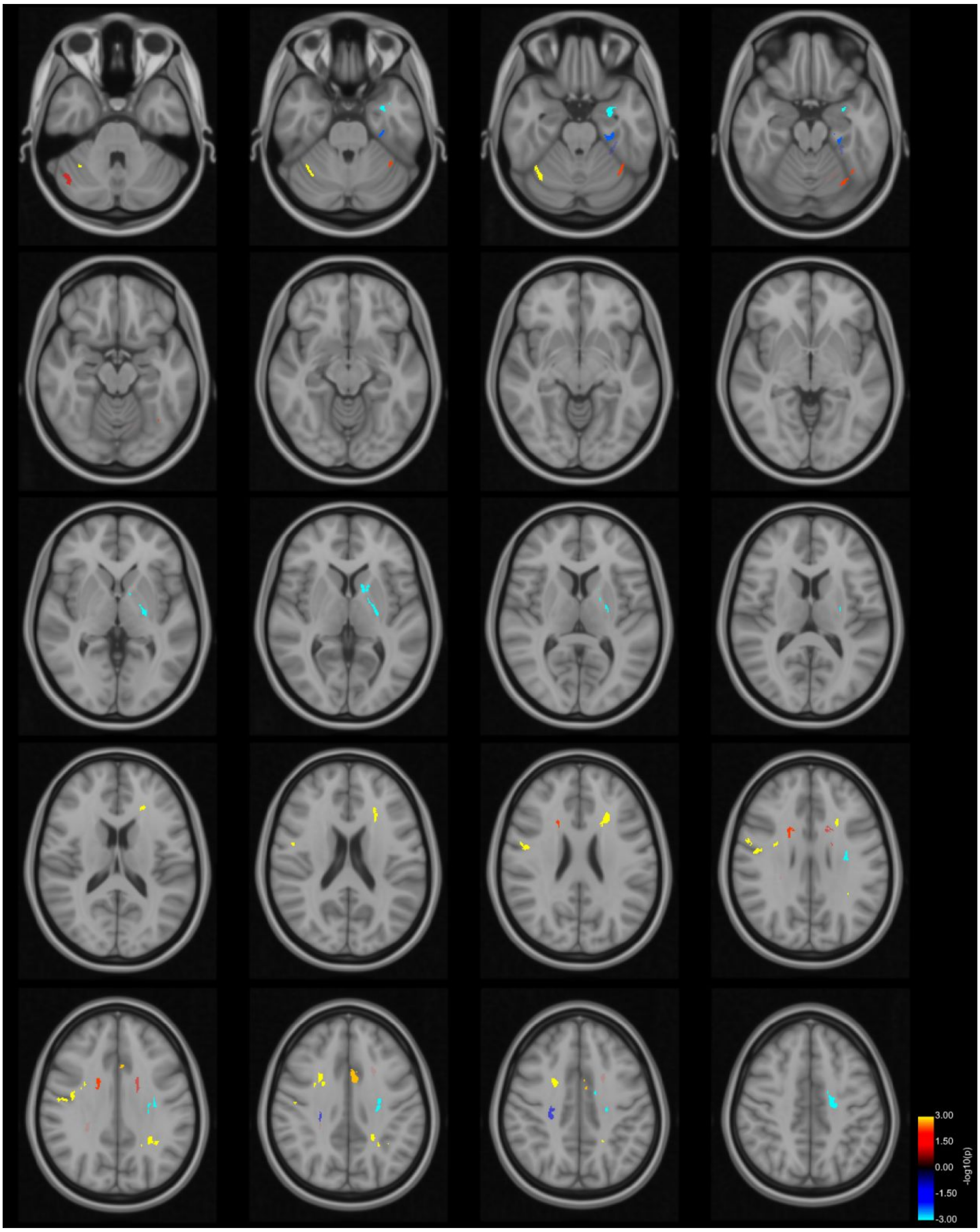

Figure 6. Clusters with a lower (blue to light blue colour) or higher (red to yellow colour) slope in Parkinson's disease patients compared to HC in the association of participants' ODI-values and error rates (red to yellow colour) as revealed by the whole brain analysis. P-Values were corrected for multiple comparisons using a permutation-based approach. Results are displayed as the negative decadic logarithm of the p-value ($p=10^{-x}$).

4. Discussion

In the present study, we investigated the relationship between microstructural integrity and bimanual motor control in Parkinson's disease using diffusion-weighed MRIs of patients with Parkinson's disease and healthy controls matched for age, sex, handedness, and cognitive status. We found spatially specific white matter abnormalities in projection and association fibers that predicted poor bimanual performance in Parkinson's disease. Moreover, reduced microstructural integrity in Parkinson's disease patients within the left limbic system, commissural fibers, bilateral pre- and postcentral gyrus, and the cerebellum predicted impaired bimanual coordination.

4.1 Alterations of microstructure in patients with Parkinson's disease

Microstructural abnormalities in Parkinson's disease have been studied extensively and a multifocal pattern of DTI changes has been described.[9] Consistent across the majority of studies investigating microstructural changes in Parkinson's disease, however, is a reduced FA in substantia nigra (SN).[9,36,37] In the present study, we replicated the finding of reduced FA-values in bilateral SN in Parkinson's disease patients. It is noteworthy, that most of the above-mentioned studies employed a region of interest (ROI) approach selecting the SN as seed ROI, whereas in our study, a whole brain approach with a rigorous correction for multiple testing was implemented. Furthermore, we found decreased AD, increased RD, as well as increased ICVF and ODI in Parkinson's disease patients comprising multiple subcortical, cortical, and cerebellar structures. Several of these alterations have been reported previously and linked to distinct motor and non-motor dysfunctions.[9]

Interpretation of increased AD- and ICVF- as well as decreased RD-values is a matter of ongoing debate. At present, neuropathologic validation for these changes is lacking and some authors argue that at least some of these changes might be due to methodological confounding factors.[9] Thus, we refrain from interpreting these clusters.

For Review Only

Concluding, we found a distinct profile of microstructural changes in Parkinson's disease replicating major findings consistent across the literature and in accordance with known Parkinson's disease pathophysiology underlining the robustness of our findings.

4.2 Interaction between microstructural changes and behavioural data in white matter tracts

Reduced FA and increased ODI in Parkinson's disease patients within the left anterior thalamic radiation predicted poor bimanual performance. The AThR interconnects the anterior and midline nuclei of the thalamus with the frontal lobe, particularly the dorsolateral prefrontal cortex (DLPFC) and the premotor area.[38,39] Besides the input from frontal and premotor areas, the respective nuclei receive input from gyrus cinguli, and the pallidum.[39] Therefore, they are associated with the limbic system and thought to be involved in executive functions and planning of complex behaviour. The prefrontal cortex, on the other hand, is implicated in attention to action, online movement monitoring, and working memory retrieval.[40,41] Particularly, left DLPFC is thought to be involved in task setting and switching.[42] As high levels of attention were required to accomplish our bimanual paradigm, reduced integrity in AThR is essential in this context. One possible explanation might be that microstructural alterations in this tract hamper the accurate relaying of motor and memory information between the pallidum and cingulum on the one hand and the prefrontal cortex on the other. In fact, it has consistently been shown that reduced FA-values in AThR are associated with poor executive function in patients with bipolar disorder[43,44] and movement dysfunction in Parkinson's disease.[45,46]

The association of reduced AD- and increased RD- and ODI-values in bilateral SLF with poor bimanual coordination suggests that altered information processing between frontal and parietal areas, connected via SLF,[47] is a contributing factor to reduced bimanual coordination in Parkinson's disease. A recent study suggests that the large fiber tract can be subdivided into four parts which facilitate distinct functions including visuospatial attention and motor control

(dorsal SLF), auditory comprehension and articulatory processing (ventral SLF), language-related processing (posterior SLF), and language-related activities such as phonological processing (arcuate fasciculus, AF).[47] Although the JHU-atlas employed in this study does not delineate the SLF into four parts, it comprises SLF and a temporal SLF (tSLF). tSLF includes the trajectories to the temporal lobe and corresponds to the posterior SLF and parts of the AF in Nakajima et al.[47,48] Since altered diffusivity was observed in SLF only – and not tSLF – we conclude that primarily dorsal and ventral parts of the SLF were affected in our population. Both subdivisions are broadly involved in motor control, whereby a hemispheric predominance exists. Right hemispheric SLF is involved in visuomotor processing and spatial working memory, whereas left hemispheric SLF is involved in motor planning and recognition of postural changes.[47] Furthermore, the dorsal SLF is a major part of the dorsal pathway of attention mediating visuospatial awareness and disruption of this pathway due to stroke or tumor results in contralateral spatial neglect.[47] Based on these findings and the results of our analysis we suggest that compromised microstructure of SLF contributes to impaired motor planning, visuomotor processing, spatial navigation, and disruption of attentional circuits subsequently giving rise to constrained bimanual coordination.

The prediction of poor bimanual coordination by reduced AD-values in ILF and IFOF suggests that altered processing of visual information is a contributing factor to poor bimanual coordination in Parkinson's disease. The IFOF connects the frontal with the temporal and occipital lobes.[39] Functions thought to be mediated by IFOF include attention and visual processing.[49] The ILF, on the other hand, is involved in visual memory and object, face, and place processing and is therefore particularly important for visually guided behaviour.[50] Compromised microstructure might impede the conveyance of visual information between occipital, temporal, and frontal lobes and therefore hamper neural integration of visual feedback. The role of compromised ILF and IFOF microstructure in Parkinson's disease has

For Review Only

been examined by a recent study linking the occurrence of visual hallucinations to reduced FA-values in both tracts.[51] Concluding, evidence exists that microstructural alterations in ILF and IFOF impairs visual information processing which contributes to the evolvement of specific clinical symptoms and can be associated to impaired behavioural measures. In the context of Parkinson's disease, our results suggest that compromised microstructure in both fiber tracts can be a contributing factor to reduced bimanual coordination.

4.3 Interaction between microstructural changes and behavioural data in grey matter

Microstructural alterations within grey matter can also be detected employing DTI. As cellular membranes of neurons are not aligned in one preferential direction, anisotropy is low.[52] Thus, diffusivity measures such as AD and RD as well as NODDI-parameters are appropriate metrics to investigate compromised microstructure due to a breakdown of cellular barriers resulting in increased diffusivity.[52] In the present study, altered microstructure of grey matter predicted poor bimanual coordination of Parkinson's disease patients in bilateral pre- and postcentral gyrus, left hippocampus, and bilateral cerebellum. Furthermore, alterations in bilateral CST and cingulum are discussed here due to their close functional relation to pre- and postcentral gyrus and hippocampus respectively.

Reduced AD and increased ODI within and in close proximity to bilateral pre- and postcentral gyrus and CST was associated with increased *error rates*. These findings indicate that microstructure within the major effectors of motor control is compromised in Parkinson's disease which might contribute to poor bimanual coordination. Previous studies have shown congruent results and related alterations in these areas to overall motor dysfunction as measured by the UPDRS part III (Unified Parkinson's Disease Rating Scale).[9,53] Changes in microstructure in postcentral gyrus, in the proximity of the somatosensory cortex, indicate difficulties in integrating sensory information in Parkinson's disease. Zhan and colleagues have

For Review Only

previously described microstructural alterations in postcentral gyrus in Parkinson's disease patients and suggested that these changes are associated with sensory response abnormalities.[53] Acknowledging the important role of sensory feedback in motor planning and execution,[54] we speculate that altered microstructure in postcentral gyrus affects sensory feedback processing and subsequently contributes to impaired bimanual control.

The fact that reduced AD and increased ODI (left cingulum only) in left hippocampus, parahippocampus, and bilateral cingulum predicted higher *error rates* indicates that impaired processing of memory information is a contributing factor to impaired bimanual coordination in Parkinson's disease. The aforementioned structures are the key areas involved in registration, storage, and retrieval of memory information and thus essential for memory processing.[39] In Parkinson's disease, compromised microstructure in these areas has been associated with reduced scores in cognitive assessments,[55] impaired visuospatial memory,[56] dementia,[57] and visual hallucinations.[56] Although participants in this study showed normal neuropsychological test scores and did not report visual hallucinations, even subtle changes in the aforementioned areas seem to contribute to impaired bimanual coordination.

Several changes in microstructure between HC and Parkinson's disease patients were observed in the cerebellum. An association with reduced bimanual coordination, however, was revealed in bilateral lobule VI only. The cerebellum can be divided into three functional subdivisions comprising: (i) motor, (ii) attentional/executive, and (iii) default-mode processing.[58] According to this classification, lobule VI is implicated in motor and particularly attentional and executive function.[58] In this regard, Guell and colleagues could demonstrate that lobule VI activation was present during finger tapping/toe grasping movements and 2-back working memory conditions.[59] In the present study, participants recalled and tapped the sequence learned at the beginning of a session for one hand while a new sequence was tapped using the other hand. Therefore, utilizing working memory was vital in our paradigm. Hence, our results suggest that

For Review Only

altered microstructure in lobule VI in Parkinson's disease impairs motor and working memory processing which might contribute to poor bimanual coordination.

4.4 Limitations

Several limitations pertaining to our study have to be addressed. First, interpretation of DTI-derived indices in areas of low anisotropy (i.e. areas of complex axonal or dendritic architecture) is difficult. Second, although histopathological validation of NODDI exists and its use has been validated in Parkinson's disease, no studies validating the neurite morphology revealed by NODDI in post-mortem brain tissue of Parkinson's disease patients exists. Third, we interpret our results in light of the functional role of a respective structure described in the literature. This represents a limitation, as we did not employ tasks to test for attention, working memory, or executive function separately. Testing for these functions, however, would have been impractical. Fourth, it is difficult to co-register the neocortex of every subject to a common space due to the broad inter-individual variety of the neocortex. Therefore, voxels with non-brain tissue in at least one subject were excluded.

5. Conclusion

In conclusion, we describe a spatially distinct profile of microstructural alterations associated with poor bimanual coordination exceeding previously reported alterations related to overall movement dysfunction. Combining known functional topography with the present findings, structures important for attentional networks, working memory, executive function, overall motor control and planning, as well as visual processing are affected and contribute to poor bimanual coordination in Parkinson's disease.

6. Acknowledgement

The authors would like to thank the participants for their active engagement in this study.

For Review Only

7. Author contributions

1. Research project: A. Conception, B. Organization, C. Execution; 2. Statistical Analysis: A. Design, B. Execution, C. Review and Critique; 3. Manuscript Preparation: A. Writing of the first draft, B. Review and Critique;

PAL: 1.A, 1.B, 1.C, 2.A, 2.B, 3.A, 3.B

IW: 1.A, 1.B, 1.C, 2.C, 3.B

CRO: 1.C, 2.C, 3.B

FSN: 1.A, 1.B, 1.C, 2.C, 3.B

HSD: 1.C, 2.C, 3.B

MT: 1.B, 1.C, 2.C, 3.B

SK: 1.C, 2.C, 3.B

LT: 1.A, 1.B, 2.C, 3.B

MB: 1.A, 1.B, 2.A, 2.B, 2.C, 3.B

8. Funding

PAL was supported by the SUCCESS-Program of the Philipps-University of Marburg and the 'Stiftung zur Förderung junger Neurowissenschaftler'. IW reports no financial disclosures. CRO was supported by the von Behring-Roentgen-Foundation (Grant 66-0014) and the Thiemann Fellowship of the Thiemann Foundation. FSN reports no financial disclosures. HSD was supported by KölnFortune, the Thiemann Fellowship of the Thiemann Foundation and the Felgenhauer Foundation. MT reports no financial disclosures. SK reports no financial disclosures. L.T. received payments as a consultant for Medtronic Inc. and Boston Scientific and received honoraria as a speaker on symposia sponsored by Bial, Zambon Pharma, UCB Schwarz Pharma, Desitin Pharma, Medtronic, Boston Scientific, and Abbott. The institution of L.T., not L.T. personally, received funding by the German Research Foundation, the German

For Review Only

Ministry of Education and Research, and Deutsche Parkinson Vereinigung. MB reports no financial disclosures.

9. Competing interests

The authors report no competing interests

10. References

1. Nettersheim FS, Loehrer PA, Weber I, et al. Dopamine substitution alters effective connectivity of cortical prefrontal, premotor, and motor regions during complex bimanual finger movements in Parkinson's disease. *NeuroImage.* 2019;190:118-132.

2. Foki T, Vanbellingen T, Lungu C, et al. Limb-kinetic apraxia affects activities of daily living in Parkinson's disease: a multi-center study. *European journal of neurology.* 2016;23(8):1301-1307.

3. Ponsen MM, Daffertshofer A, van den Heuvel E, Wolters E, Beek PJ, Berendse HW. Bimanual coordination dysfunction in early, untreated Parkinson's disease. *Parkinsonism Relat Disord.* 2006;12(4):246-252.

4. Loehrer PA, Nettersheim FS, Jung F, et al. Ageing changes effective connectivity of motor networks during bimanual finger coordination. *NeuroImage.* 2016;143:325-342.

5. Swinnen SP. Intermanual coordination: from behavioural principles to neural-network interactions. *Nature reviews Neuroscience.* 2002;3(5):348-359.

6. Wu T, Wang L, Hallett M, Li K, Chan P. Neural correlates of bimanual anti-phase and in-phase movements in Parkinson's disease. *Brain : a journal of neurology.* 2010;133(Pt 8):2394-2409.

For Review Only

7. Loehrer PA, Nettersheim FS, Oehrn CR, et al. Increased prefrontal top-down control in older adults predicts motor performance and age-group association. *NeuroImage.* 2021;240:118383.

8. Dickson DW, Braak H, Duda JE, et al. Neuropathological assessment of Parkinson's disease: refining the diagnostic criteria. *The Lancet Neurology.* 2009;8(12):1150-1157.

9. Zhang Y, Burock MA. Diffusion Tensor Imaging in Parkinson's Disease and Parkinsonian Syndrome: A Systematic Review. *Frontiers in neurology.* 2020;11:531993.

10. De Micco R, Russo A, Tessitore A. Structural MRI in Idiopathic Parkinson's Disease. *International review of neurobiology.* 2018;141:405-438.

11. Zhang H, Schneider T, Wheeler-Kingshott CA, Alexander DC. NODDI: Practical in vivo neurite orientation dispersion and density imaging of the human brain. *NeuroImage.* 2012;61(4):1000-1016.

12. Kamagata K, Zalesky A, Hatano T, et al. Gray Matter Abnormalities in Idiopathic Parkinson's Disease: Evaluation by Diffusional Kurtosis Imaging and Neurite Orientation Dispersion and Density Imaging. *Human brain mapping.* 2017;38(7):3704-3722.

13. Oldfield RC. The assessment and analysis of handedness: The Edinburgh inventory. *Neuropsychologia.* 1971;9(1):97-113.

14. Beck AT, Steer RA, Brown GK. Manual for the beck depression inventory-II. *San Antonio, TX: Psychological Corporation.* 1996;1:82.

For Review Only

15. Folstein MF, Folstein SE, McHugh PR. "Mini-mental state": A practical method for grading the cognitive state of patients for the clinician. *Journal of Psychiatric Research.* 1975;12(3):189-198.

16. Kalbe E, Kessler J, Calabrese P, et al. DemTect: a new, sensitive cognitive screening test to support the diagnosis of mild cognitive impairment and early dementia. *International Journal of Geriatric Psychiatry.* 2004;19(2):136-143.

17. Ségonne F, Dale AM, Busa E, et al. A hybrid approach to the skull stripping problem in MRI. *NeuroImage.* 2004;22:1060-1075.

18. Fischl B, Salat DH, Busa E, et al. Whole brain segmentation: automated labeling of neuroanatomical structures in the human brain. *Neuron.* 2002;33:341-355.

19. Sled JG, Zijdenbos AP, Evans AC. A nonparametric method for automatic correction of intensity nonuniformity in MRI data. *IEEE Trans Med Imaging.* 1998;17:87-97.

20. Fischl B, Liu A, Dale AM. Automated manifold surgery: constructing geometrically accurate and topologically correct models of the human cerebral cortex. *IEEE Trans Med Imaging.* 2001;20:70-80.

21. Ségonne F, Pacheco J, Fischl B, Segonne F, Pacheco J, Fischl B. Geometrically accurate topology-correction of cortical surfaces using nonseparating loops. *IEEE Trans Med Imaging.* 2007;26:518-529.

22. Dale AM, Fischl B, Sereno MI. Cortical surface-based analysis. I. Segmentation and surface reconstruction. *NeuroImage.* 1999;9:179-194.

23. Jenkinson M, Smith S. A global optimisation method for robust affine registration of brain images. *Medical Image Analysis.* 2001;5:143-156.

For Review Only

24. Greve DN, Fischl B. Accurate and robust brain image alignment using boundary-based registration. *NeuroImage.* 2009;48:63-72.

25. Pierpaoli C, Basser PJ. Toward a quantitative assessment of diffusion anisotropy. *Magnetic Resonance in Medicine.* 1996;36:893-906.

26. Budde MD, Joong HK, Liang HF, et al. Toward accurate diagnosis of white matter pathology using diffusion tensor imaging. *Magnetic Resonance in Medicine.* 2007;57:688-695.

27. Song SK, Sun SW, Ju WK, Lin SJ, Cross AH, Neufeld AH. Diffusion tensor imaging detects and differentiates axon and myelin degeneration in mouse optic nerve after retinal ischemia. *NeuroImage.* 2003;20:1714-1722.

28. Song SK, Sun SW, Ramsbottom MJ, Chang C, Russell J, Cross AH. Dysmyelination revealed through MRI as increased radial (but unchanged axial) diffusion of water. *NeuroImage.* 2002;17:1429-1436.

29. Song SK, Sun SW, Ju WK, Lin SJ, Cross AH, Neufeld AH. Diffusion tensor imaging detects and differentiates axon and myelin degeneration in mouse optic nerve after retinal ischemia. *NeuroImage.* 2003;20(3):1714-1722.

30. Edwards LJ, Pine KJ, Ellerbrock I, Weiskopf N, Mohammadi S. NODDI-DTI: Estimating Neurite Orientation and Dispersion Parameters from a Diffusion Tensor in Healthy White Matter. *Frontiers in Neuroscience.* 2017;11(720).

31. *DTI-NODDI. Implementation of diffusion tensor image based neurite orientation dispersion and density imaging (DTI-NODDI) written in Python. Available from https://github.com/dicemt/DTI-NODDI* [computer program]. https://github.com/dicemt/DTI-NODDI2020.

For Review Only

32. Andersson JLR, Jenkinson M, Smith S. Non-linear registration, aka spatial normalisation. - FMRIB technical report TR07JA2 from wwwfmriboxacuk/analysis/techrep. 2007.

33. Jenkinson M, Bannister P, Brady M, Smith S. Improved optimization for the robust and accurate linear registration and motion correction of brain images. *NeuroImage.* 2002;17:825-841.

34. Diffusion Tensor Imaging (DTI) in idiopathic REM sleep behaviour disorder (iRBD), Klinische Neurophysiologie(2010).

35. Nichols TE, Holmes AP. Nonparametric permutation tests for functional neuroimaging: A primer with examples. *Human brain mapping.* 2002;15:1-25.

36. Péran P, Cherubini A, Assogna F, et al. Magnetic resonance imaging markers of Parkinson's disease nigrostriatal signature. *Brain : a journal of neurology.* 2010;133(11):3423-3433.

37. Scherfler C, Esterhammer R, Nocker M, et al. Correlation of dopaminergic terminal dysfunction and microstructural abnormalities of the basal ganglia and the olfactory tract in Parkinson's disease. *Brain : a journal of neurology.* 2013;136(10):3028-3037.

38. George K MDJ. Neuroanatomy, Thalamocortical Radiations. *StatPearls [Internet]* 2020; https://www.ncbi.nlm.nih.gov/books/NBK546699/. Accessed 31.05.2021.

39. Bähr M, Frotscher M. Neurologisch-topische Diagnostik Anatomie - Funktion - Klinik. 2014.

40. Jueptner M, Stephan KM, Frith CD, Brooks DJ, Frackowiak RSJ, Passingham RE. Anatomy of Motor Learning. I. Frontal Cortex and Attention to Action. *Journal of Neurophysiology.* 1997;77(3):1313-1324.

For Review Only

41. Rowe J, Friston K, Frackowiak R, Passingham R. Attention to action: specific modulation of corticocortical interactions in humans. *NeuroImage.* 2002;17(2):988-998.

42. Stuss DT, Alexander MP. Is there a dysexecutive syndrome? *Philosophical Transactions of the Royal Society B: Biological Sciences.* 2007;362(1481):901-915.

43. Niida R, Yamagata B, Niida A, Uechi A, Matsuda H, Mimura M. Aberrant Anterior Thalamic Radiation Structure in Bipolar Disorder: A Diffusion Tensor Tractography Study. *Frontiers in Psychiatry.* 2018;9(522).

44. Oertel-Knöchel V, Reinke B, Alves G, et al. Frontal white matter alterations are associated with executive cognitive function in euthymic bipolar patients. *Journal of Affective Disorders.* 2014;155:223-233.

45. Shine JM, Matar E, Ward PB, et al. Exploring the cortical and subcortical functional magnetic resonance imaging changes associated with freezing in Parkinson's disease. *Brain : a journal of neurology.* 2013;136(4):1204-1215.

46. Tan SYZ, Keong NCH, Selvan RMP, et al. Periventricular White Matter Abnormalities on Diffusion Tensor Imaging of Postural Instability Gait Disorder Parkinsonism. *American Journal of Neuroradiology.* 2019;40(4):609-613.

47. Nakajima R, Kinoshita M, Shinohara H, Nakada M. The superior longitudinal fascicle: reconsidering the fronto-parietal neural network based on anatomy and function. *Brain Imaging and Behavior.* 2020;14(6):2817-2830.

48. Wakana S, Caprihan A, Panzenboeck MM, et al. Reproducibility of quantitative tractography methods applied to cerebral white matter. *NeuroImage.* 2007;36(3):630-644.

For Review Only

49. Catani M, Thiebaut de Schotten M. A diffusion tensor imaging tractography atlas for virtual in vivo dissections. *Cortex; a journal devoted to the study of the nervous system and behavior.* 2008;44(8):1105-1132.

50. Herbet G, Zemmoura I, Duffau H. Functional Anatomy of the Inferior Longitudinal Fasciculus: From Historical Reports to Current Hypotheses. *Frontiers in Neuroanatomy.* 2018;12(77).

51. Lenka A, Ingalhalikar M, Shah A, et al. Abnormalities in the white matter tracts in patients with Parkinson disease and psychosis. *Neurology.* 2020;94(18):e1876-e1884.

52. Pierpaoli C, Jezzard P, Basser PJ, Barnett A, Di Chiro G. Diffusion tensor MR imaging of the human brain. *Radiology.* 1996;201(3):637-648.

53. Zhan W, Kang GA, Glass GA, et al. Regional alterations of brain microstructure in Parkinson's disease using diffusion tensor imaging. *Movement Disorders.* 2012;27(1):90-97.

54. Cheron G, Dan B, Borenstein S. Sensory and motor interfering influences on somatosensory evoked potentials. *Journal of clinical neurophysiology : official publication of the American Electroencephalographic Society.* 2000;17(3):280-294.

55. Carlesimo GA, Piras F, Assogna F, Pontieri FE, Caltagirone C, Spalletta G. Hippocampal abnormalities and memory deficits in Parkinson disease. *Neurology.* 2012;78(24):1939.

56. Shine JM, Halliday GM, Naismith SL, Lewis SJG. Visual misperceptions and hallucinations in Parkinson's disease: Dysfunction of attentional control networks? *Movement Disorders.* 2011;26(12):2154-2159.

For Review Only

57. Chen B, Fan GG, Liu H, Wang S. Changes in anatomical and functional connectivity of Parkinson's disease patients according to cognitive status. *European Journal of Radiology.* 2015;84(7):1318-1324.

58. Guell X, Schmahmann J. Cerebellar Functional Anatomy: a Didactic Summary Based on Human fMRI Evidence. *The Cerebellum.* 2020;19(1):1-5.

59. Guell X, Gabrieli JDE, Schmahmann JD. Triple representation of language, working memory, social and emotion processing in the cerebellum: convergent evidence from task and seed-based resting-state fMRI analyses in a single large cohort. *NeuroImage.* 2018;172:437-449.

11. Figure legends

Figure 1. Instructions presented on a screen (left) and demanded button presses (right) in chronological order. Participants learned a sequence for their left hand (here: 1|2|3|4) and tapped a new sequence with their right hand. At the beginning of each trial, the upcoming sequence was presented in red (here: 4|3|2|1) and signalled the subject to prepare for the next trial. The switch from red numbers to green numbers served as "go"-signal, indicating to commence tapping. A red cross, followed by a short break of 5 seconds, marked the end of one trial. In this example, the first requested pair of button presses was left thumb and right ring finger, followed by left index and right middle finger. The third requested pair of button presses was left middle and right index finger followed by left ring finger and right thumb. Participants tapped in synchrony and were instructed to favour correct trial execution over speed. (Figure adapted from Loehrer et al., 2016).

Figure 2. Association between white matter fractional anisotropy in left anterior thalamic radiation and participants' error rates as revealed by GLM. Lower FA-values predicted higher error rates in Parkinson's disease patients, whereas no significant association existed for healthy controls.

For Review Only

Figure 3. Clusters with a lower slope (blue to light blue colour) in the analysis of Parkinson's disease patients' FA-values and error rates in comparison to the association of FA-values and error rates of HC as revealed by the whole brain analysis. P-Values were corrected for multiple comparisons using a permutation-based approach. Results are displayed as the negative decadic logarithm of the p-value ($p=10^{-x}$).

Figure 4. Clusters with a lower (blue to light blue colour) or higher (red to yellow colour) slope in Parkinson's disease patients compared to HC in the association of participants' AD-values and error rates (only lower slopes depicted), as well as RD-values and error rates (only higher slopes depicted) as revealed by the whole brain analysis. P-Values were corrected for multiple comparisons using a permutation-based approach. Results are displayed as the negative decadic logarithm of the p-value ($p=10^{-x}$).

Figure 5. Clusters with a lower (blue to light blue colour) or higher (red to yellow colour) slope in Parkinson's disease patients compared to HC in the association of participants' ICVF-values and error rates as revealed by the whole brain analysis. P-Values were corrected for multiple comparisons using a permutation-based approach. Results are displayed as the negative decadic logarithm of the p-value ($p=10^{-x}$).

Figure 6. Clusters with a lower (blue to light blue colour) or higher (red to yellow colour) slope in Parkinson's disease patients compared to HC in the association of participants' ODI-values and error rates (red to yellow colour) as revealed by the whole brain analysis. P-Values were corrected for multiple comparisons using a permutation-based approach. Results are displayed as the negative decadic logarithm of the p-value ($p=10^{-x}$).

For Review Only

Supplementary Material

Age (years)	Gender	EHI	MMSE	DemTect	BDI-2
52	F	82	30	18	0
54	M	85	30	18	0
64	M	8	30	18	0
52	M	60	29	18	0
57	M	75	28	18	5
63	M	88	30	18	3
62	M	91	29	18	0
68	M	75	30	18	0
62	F	83	29	15	0
62	F	92	30	18	2
50	M	83	30	18	0
56	M	100	29	17	1
62	M	100	28	18	0
68	M	100	29	17	3
52	F	91	30	18	3
62	F	64	30	15	10
55	F	91	30	18	1
56	M	94	30	18	3
68	M	78	30	18	1
50	M	100	30	18	2
62	M	5	30	16	0
56	F	76	29	18	2
58	F	100	30	18	0
56	F	100	28	18	0
61	F	100	29	18	0
53	F	100	28	17	5

For Review Only

Mean: 58.5 SD: 5.5	Ratio: F:M 11:15	Mean: 81.6 SD: 24.9	Mean: 29.4 SD: .8	Mean: 17.6 SD: .9	Mean: 1.6 SD: 2.3

Supplementary Table 1. Sociodemographic data and neuropsychological test scores of healthy controls. BDI-2 = Beck's Depression Inventory 2; DemTect = Dementia Detection Test; EHI = Edinburgh Handedness Inventory; F = female; M = male; MMSE = Minimental State Examination; SD = standard deviation

For Review Only

Age (years)	Gender	EHI	MMSE	DemTect	BDI-2
52	M	26	25	15	11
43	M	41	29	18	10
54	M	100	30	15	13
51	M	100	29	18	9
46	M	88	28	18	9
64	F	75	30	18	2
48	F	100	29	18	1
63	F	58	30	17	11
64	F	90	29	18	7
60	F	92	30	17	13
58	M	75	29	18	1
49	M	68	29	14	7
57	F	45	30	17	7
61	M	100	29	16	6
64	M	83	27	18	4
65	M	70	28	15	4
50	M	83	29	16	4
61	F	43	30	16	11
58	F	92	30	18	4
56	M	89	29	18	2
49	M	67	28	17	3
58	M	92	29	14	9
57	M	100	29	18	3
Mean: 56.0 SD: 6.5	Ratio: F:M 8:15	Mean: 77 SD: 21.8	Mean: 28.9 SD: 1.2	Mean: 16.8 SD: 1.4	Mean: 6.6 SD: 3.9

Supplementary Table 2. Neuropsychological test scores of Parkinson's disease patients. BDI-2 = Beck's Depression Inventory 2; DemTect = Dementia Detection Test; EHI = Edinburgh Handedness Inventory; F = female; M = male; MMSE = Minimental State Examination; SD = standard deviation

For Review Only

Negative Cluster	Location	p-Value	Volume in mm³	MNI152-Coordinates X	Y	Z
1	Left red nucleus<br>Left ncl. reticularis polaris<br>Left substantia nigra	0.024	245	-6	-16	-9
2	Right ncl. reticularis polaris<br>Right red nucleus<br>Right substantia nigra	0.031	236	8	-15	-4
Positive Cluster						
1	Left corticospinal tract	0.003	310	-18	-21	-5
2	Right inferior fronto-occipital fasciculus<br>Right inferior longitudinal fasciculus	0.009	271	27	-21	-7
3	Left cingulum (hippocampus)	0.042	224	-25	-17	-22

Supplementary Table 3. Characteristics of clusters differing in FA-values between Parkinson's disease patients and HC. "Negative Cluster" denotes clusters with reduced FA-values in Parkinson's disease patients compared to HC, whereas "Positive Cluster" denotes clusters with increased FA-values in Parkinson's disease patients. "Location" indicates the anatomical landmark comprising the majority of voxels of a cluster according to Johns Hopkins University (JHU) white matter atlas, Harvard-Oxford cortical and subcortical atlas, University College London (UCL) cerebellar atlas, and a brainstem atlas based on the DISTAL Atlas of Lead DBS. P-Values are clusterwise p-values corrected for multiple comparisons. "Volume in mm³" denotes the size of a cluster and "MNI152-coordinates" describes the coordinates of the cluster's center of gravity in MNI152-space.

For Review Only

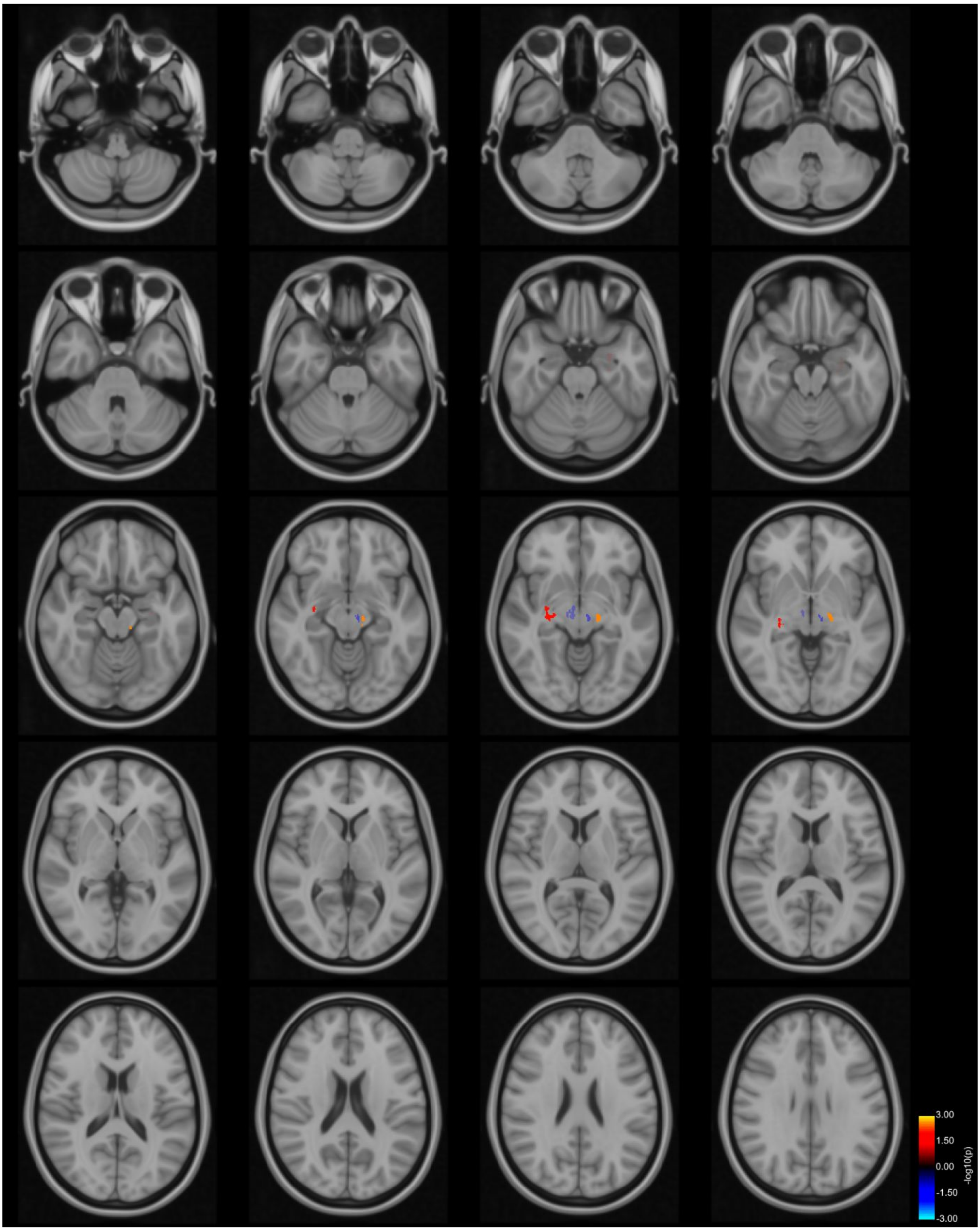

Supplementary Figure 1. Clusters differing in fractional anisotropy between Parkinson's disease patients and HC as revealed by the whole brain analysis. P-Values were corrected for multiple comparisons using a permutation-based approach. Blue to light blue colour indicates clusters with lower FA-values in Parkinson's disease patients compared to HC. Red to yellow colour indicates clusters with higher FA-values in Parkinson's disease patients compared to HC. Results are displayed as the negative decadic logarithm of the p-value ($p=10^{-x}$).

Negative Cluster	Location	p-Value	Volume in mm³	MNI152-Coordinates X	Y	Z
1	Left crus I	< .001	795	-35	-65	-22
2	Left putamen<br>Left ncl. accumbens	< .001	481	-15	6	-10
3	Forceps Major	< .001	373	3	-36	11
4	Right forceps major<br>Right precuneous cortex	< .001	340	16	-44	23
5	Right putamen<br>Right ncl. accumbens	< .001	330	17	8	-8
6	Right crus I	< .001	240	29	-67	-24
7	Right inferior fronto-occipital fasciculus	< .001	235	31	-34	3
8	Left IX	< .001	216	-9	-52	-25
9	Right VI	< .001	208	17	-66	-13
10	Left and right I-IV	.001	197	-1	-58	-15
11	Left forceps minor<br>Left frontal pole<br>Left anterior thalamic radiation	.001	193	-22	45	9
12	Left parahippocampal gyrus, anterior division	.015	159	-17	-12	-26
13	Left Cingulum (cingulate gyrus, anterior division)	.037	143	-7	-7	24
14	Right inferior fronto-occipital fasciculus<br>Right optic tract	.037	143	15	-8	-10
15	Right cingulum (cingulate gyrus, posterior division)	.048	138	13	-27	27
**Positive Cluster**						
1	Left corticospinal tract	< .001	965	-19	-13	42
2	Left Pallidum	< .001	451	-23	-9	3
3	Right corticospinal tract	< .001	306	25	-27	35
4	Left Cingulum	< .001	217	-30	-26	-19
5	Right Pallidum	.048	138	19	-7	3

Supplementary Table 4. Characteristics of clusters differing in AD-values between Parkinson's disease patients and HC. "Negative Cluster" denotes clusters with reduced AD-

values in Parkinson's disease patients compared to HC, whereby "Positive Cluster" denotes clusters with increased AD-values in Parkinson's disease patients. "Location" indicates the anatomical landmark comprising the majority of voxels of a cluster according to Johns Hopkins University (JHU) white matter atlas, Harvard-Oxford cortical and subcortical atlas, University College London (UCL) cerebellar atlas, and a brainstem atlas based on the DISTAL Atlas of Lead DBS. P-Values are clusterwise p-values corrected for multiple comparisons. "Volume in mm^3" denotes the size of a cluster and "MNI152-coordinates" describes the coordinates of the cluster's center of gravity in MNI152-space.

For Review Only

Negative Cluster	Location	p-Value	Volume in mm³	MNI152-Coordinates X	Y	Z
1	Left crus I	< .001	666	-35	-65	-21
2	Left Putamen<br>Left uncinate fasciculus	< .001	369	-17	6	-11
3	Left anterior thalamic radiation	< .001	220	-28	-28	9
4	Right forceps major<br>Right precuneous cortex	< .001	220	23	-47	22
5	Right inferior longitudinal fasciculus<br>Right corticospinal tract<br>Right inferior fronto-occipital fasciculus	.002	210	27	-22	-5
6	Left IX	.002	198	-9	-51	-26
7	Right inferior longitudinal fasciculus<br>Right planum polare	.015	164	44	-23	-9
8	Left inferior longitudinal fasciculus<br>Lateral occipital cortex, superior division	.028	153	-23	-60	32
**Positive Cluster**						
1	Left anterior thalamic radiation	.006	182	-3	-32	-22

Supplementary Table 5. Characteristics of clusters differing in RD-values between Parkinson's disease patients and HC. "Negative Cluster" denotes clusters with reduced RD-values in Parkinson's disease patients compared to HC, whereby "Positive Cluster" denotes clusters with increased RD-values in Parkinson's disease patients. "Location" indicates the anatomical landmark comprising the majority of voxels of a cluster according to Johns Hopkins University (JHU) white matter atlas, Harvard-Oxford cortical and subcortical atlas, University College London (UCL) cerebellar atlas, and a brainstem atlas based on the DISTAL Atlas of Lead DBS. P-Values are clusterwise p-values corrected for multiple comparisons. "Volume in mm³" denotes the size of a cluster and "MNI152-coordinates" describes the coordinates of the cluster's center of gravity in MNI152-space.

For Review Only

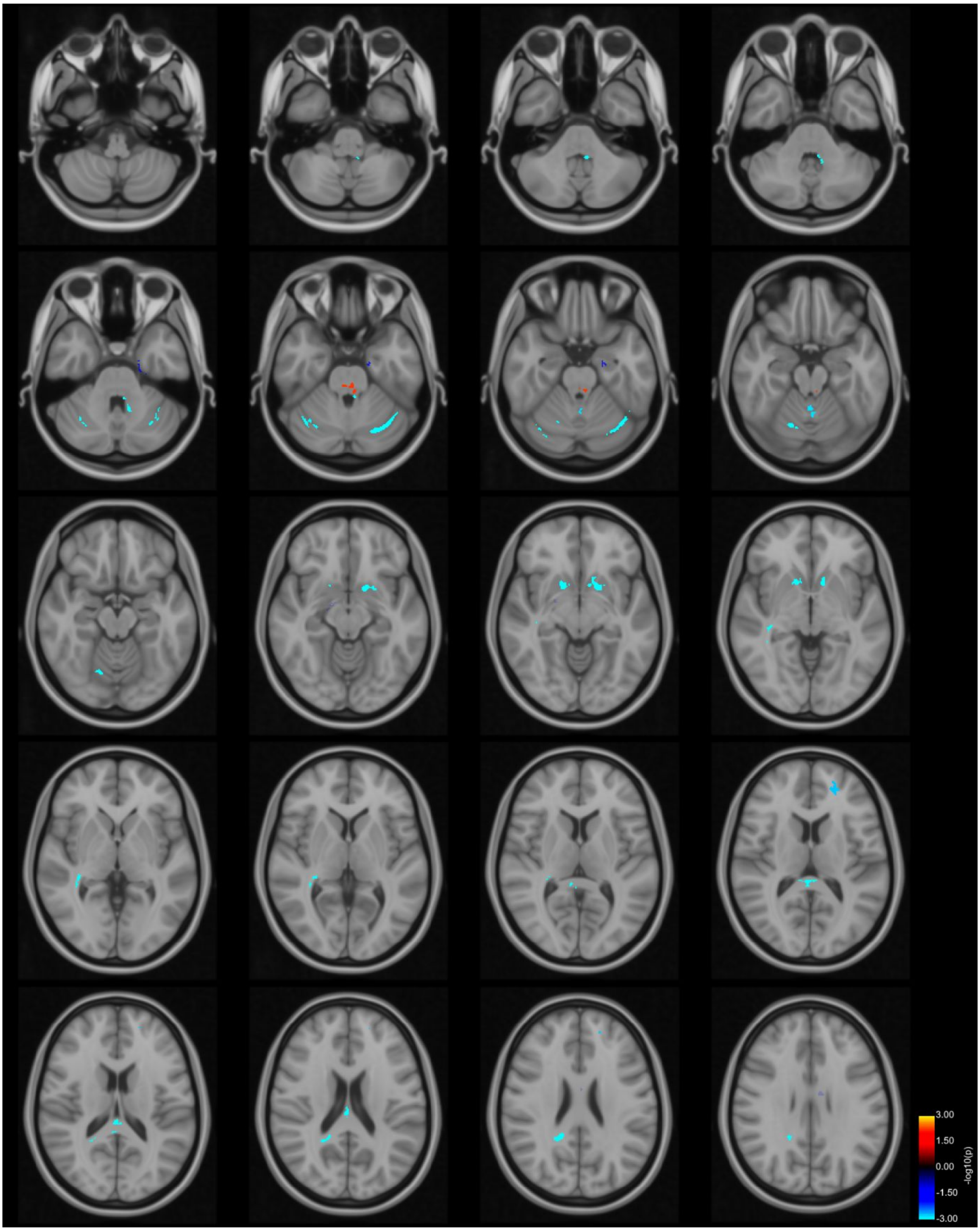

Supplementary Figure 2. Clusters differing in axial and radial diffusivity between Parkinson's disease patients and HC as revealed by the whole brain analysis. P-Values were corrected for multiple comparisons using a permutation-based approach. Blue to light blue colour indicates clusters with lower AD-values in Parkinson's disease patients compared to HC. Red to yellow colour indicates clusters with higher RD-values in Parkinson's disease compared to HC patients. Results are displayed as the negative decadic logarithm of the p-value ($p=10^{-x}$).

Positive Cluster	Location	p-Value	Volume in mm³	MNI152-Coordinates		
				X	Y	Z
1	Left crus I	< .001	798	-35	-65	-21
2	Left putamen<br>Left ncl. accumbens	< .001	658	-17	6	-10
3	Right forceps major<br>Right precuneous cortex	< .001	515	22	-49	15
4	Right inferior fronto-occipital fasciculus<br>Right inferior longitudinal fasciculus	< .001	507	31	-33	4
5	Forceps Major	< .001	448	3	-36	10
6	Left frontal pole<br>Left anterior thalamic radiation<br>Left forceps minor	.004	316	-19	41	20
7	Right putamen<br>Right anterior thalamic radiation	< .009	296	15	5	-9
8	Right crus I	.01	282	31	-65	-23
9	Right VI	.02	270	29	-58	-16

Supplementary Table 6. Characteristics of clusters differing in ICVF-values between Parkinson's disease patients and HC. "Positive Cluster" denotes clusters with increased ICVF-values in Parkinson's disease patients. "Location" indicates the anatomical landmark comprising the majority of voxels of a cluster according to Johns Hopkins University (JHU) white matter atlas, Harvard-Oxford cortical and subcortical atlas, University College London (UCL) cerebellar atlas, and a brainstem atlas based on the DISTAL Atlas of Lead DBS. P-Values are clusterwise p-values corrected for multiple comparisons. "Volume in mm³" denotes the size of a cluster and "MNI152-coordinates" describes the coordinates of the cluster's center of gravity in MNI152-space.

For Review Only

Negative Cluster	Location	p-Value	Volume in mm³	MNI152-Coordinates X	Y	Z
1	Left corticospinal tract<br>Left superior longitudinal fasciculus	< .001	963	-19	-14	42
2	Left pallidum<br>Left anterior thalamic radiation	< .001	450	-13	5	3
3	Left parahippocampal gyrus, anterior division<br>Left Amygdala	< .001	305	-26	-6	-20
4	Left parahippocampal gyrus, posterior division	.004	245	-28	-26	-21
5	Right corticospinal tract	.02	202	25	-19	36
6	Left temporal fusiform cortex, posterior division	.04	186	-28	-36	-17
**Positive Cluster**						
1	Left crus I<br>Left VI	< .001	785	-30	-68	-22
2	Left and right I-IV	.001	478	3	-53	-16
3	Left putamen<br>Left ncl. accumbens	< .001	427	-12	12	-6
4	Right putamen<br>Right ncl. Accumbens<br>Right anterior thalamic radiation	< .001	401	13	10	-6
5	Right inferior fronto-occipital fasciculus	< .001	310	39	-28	-4
6	Right crus I	.002	260	42	-59	-19
7	Right precuneous cortex<br>Right forceps major	.002	250	16	-44	23
8	Right insular cortex	.004	239	40	-8	7
9	Left anterior thalamic radiation<br>Left Thalamus	.02	201	-3	-2	-7
10	Right intracalcarine cortex<br>Right lingual gyrus	.02	195	10	-69	7
11	Right VI	.04	184	18	-67	-14

12	Left frontal pole<br>Left forceps minor<br>Left anterior thalamic radiation	.04	183	-21	51	10

Supplementary Table 7. Characteristics of clusters differing in ODI-values between Parkinson's disease patients and HC. "Negative Cluster" denotes clusters with reduced ODI-values in Parkinson's disease patients compared to HC, whereby "Positive Cluster" denotes clusters with increased ODI-values in Parkinson's disease patients. "Location" indicates the anatomical landmark comprising the majority of voxels of a cluster according to Johns Hopkins University (JHU) white matter atlas, Harvard-Oxford cortical and subcortical atlas, University College London (UCL) cerebellar atlas, and a brainstem atlas based on the DISTAL Atlas of Lead DBS. P-Values are clusterwise p-values corrected for multiple comparisons. "Volume in mm^3" denotes the size of a cluster and "MNI152-coordinates" describes the coordinates of the cluster's center of gravity in MNI152-space.

For Review Only

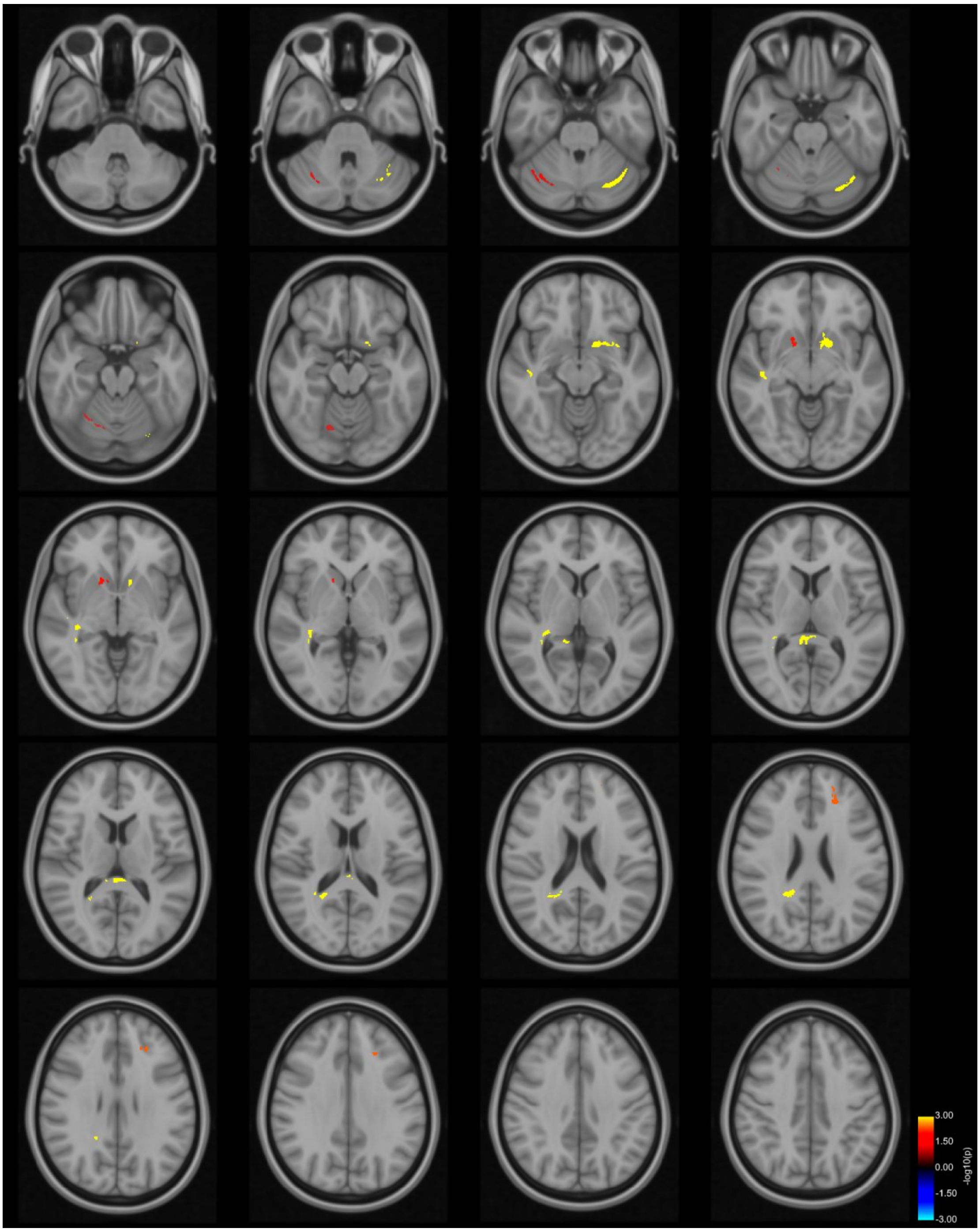

Supplementary Figure 3. Clusters differing in ICVF between Parkinson's disease patients and HC as revealed by the whole brain analysis. P-Values were corrected for multiple comparisons using a permutation-based approach. Red to yellow colour indicates clusters with higher ICVF-values in Parkinson's disease patients compared to HC. Results are displayed as the negative decadic logarithm of the p-value ($p=10^{-x}$).

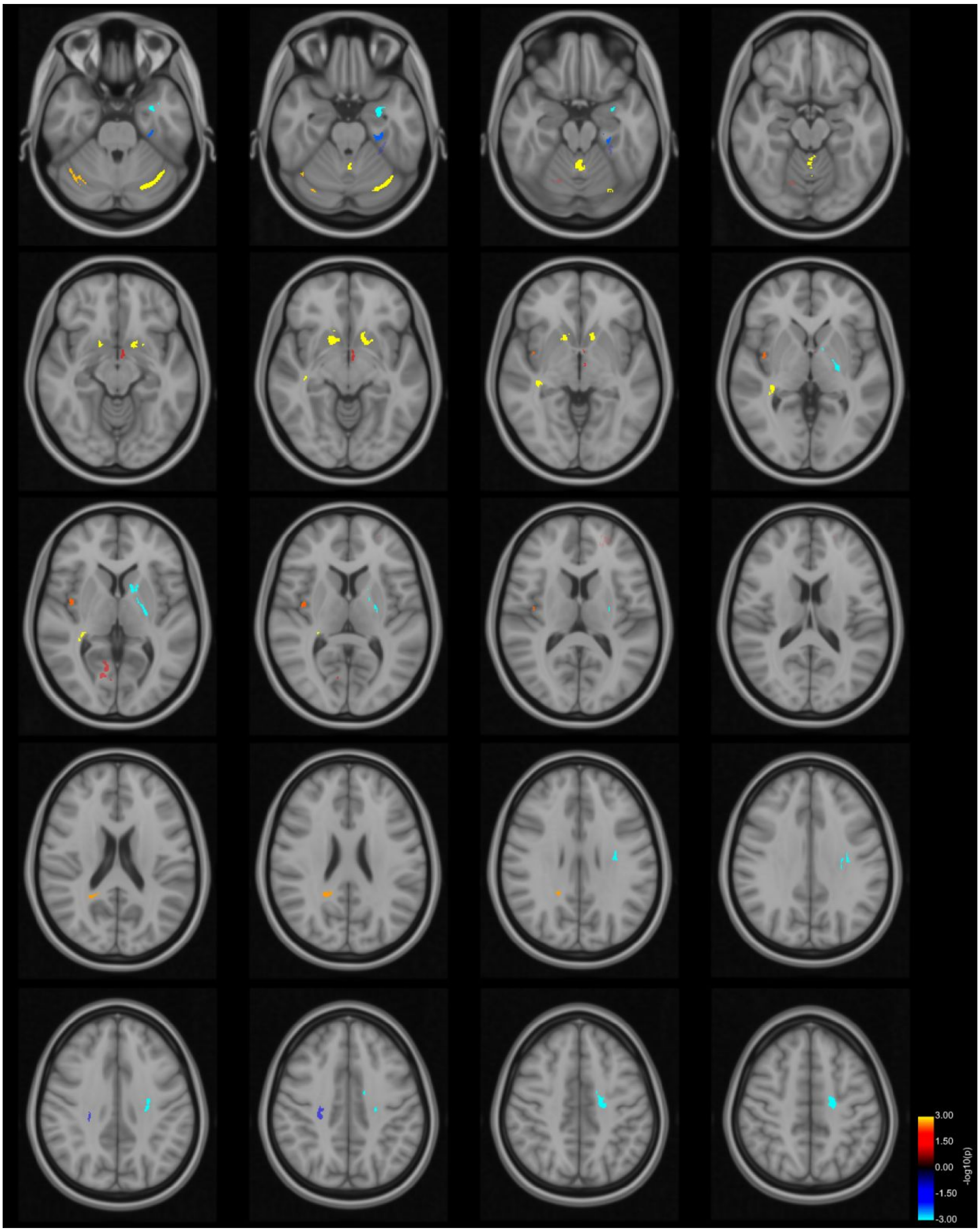

Supplementary Figure 4. Clusters differing in ODI between Parkinson's disease patients and HC as revealed by the whole brain analysis. P-Values were corrected for multiple comparisons using a permutation-based approach. Red to yellow colour indicates clusters with higher ODI-values in Parkinson's disease compared to HC patients. Blue to light blue colour indicates clusters with lower ODI-values in Parkinson's disease patients compared to HC. Results are displayed as the negative decadic logarithm of the p-value ($p=10^{-x}$).

Negative Cluster	Location	Slope (HC, Parkinson's disease)	Intercept (HC, Parkinson's disease)	p-Value	Volume in mm³	MNI152-Coordinates X	Y	Z
1	Left anterior thalamic radiation	.0004<br>-.0011	.2576<br>.341	< .001	386	-22	28	11

Supplementary Table 8. Characteristics of the cluster with an interaction between participants' FA-values and error rates. "Negative Cluster" denotes clusters with a lower slope in Parkinson's disease patients compared to HC in the association of participants' FA-values and error rates. "Location" indicates the anatomical landmark comprising the majority of voxels of a cluster according to Johns Hopkins University (JHU) white matter atlas, Harvard-Oxford cortical and subcortical atlas, University College London (UCL) cerebellar atlas, and a brainstem atlas based on the DISTAL Atlas of Lead DBS. P-Values are clusterwise p-values corrected for multiple comparisons. "Volume in mm³" denotes the size of a cluster and "MNI152-Coordinates" describes the coordinates of the cluster's center of gravity in MNI152-space.

For Review Only

Negative Cluster	Location	Slope (HC, Parkinson's disease)	Intercept (HC, Parkinson's disease)	p-Value	Volume in mm³	MNI152-Coordinates X	Y	Z
1	Right superior longitudinal fasciculus<br>Right postcentral gyrus<br>Right precentral gyrus	.0019<br>-.0011	1.0186<br>1.1395	< .001	523	49	-7	23
2	Right superior longitudinal fasciculus<br>Right corticospinal tract	.0012<br>-.0011	1.015<br>1.1587	< .001	310	27	-36	29
3	Right cingulum (cingulate gyrus, anterior division)<br>Forceps minor	.0021<br>-.0012	1.2089<br>1.331	< .001	300	14	8	26
4	Left VI	.003<br>-.0011	.93<br>1.0802	< .001	265	-21	-59	-16
5	Left cingulum (cingulate gyrus, anterior division)<br>Forceps minor<br>Left superior longitudinal fasciculus	.0025<br>-.0011	1.1592<br>1.2715	< .001	264	-17	-2	28
6	Left corticospinal tract<br>Left superior longitudinal fasciculus<br>Left precentral gyrus	.0012<br>-.0007	.9034<br>1.0072	< .001	234	-24	-10	32
7	Left corticospinal tract<br>Left superior longitudinal fasciculus<br>Left insular cortex	.0012<br>-.001	1.2084<br>1.3106	< .001	201	-29	-22	16
8	Left superior longitudinal fasciculus<br>Left middle temporal gyrus	.0013<br>-.0018	1.0582<br>1.1933	< .001	177	-50	-37	-4

9	Left ncl. accumbens<br>Left uncinate fasciculus	.002<br>-.0012	.8757<br>.9955	< .001	166	-8	5	-11
10	Right VI<br>Right crus I	.0025<br>-.0035	1.0054<br>1.2659	.01	165	37	-57	-20
11	Left Cingulum (hippocampus)<br>Left parahippocampal gyrus, anterior division	.0053<br>-.0003	.8349<br>1.0223	.012	160	-30	-9	-25
12	Left inferior longitudinal fasciculus<br>Left inferior fronto-occipital fasciculus<br>Left temporal occipital fusiform cortex	.0018<br>-.0019	.9678<br>1.158	.015	158	-40	-47	-9
13	Left anterior thalamic radiation	.0012<br>-.0013	.9913<br>1.1168	.037	141	-24	19	20
14	Right superior longitudinal fasciculus<br>Right postcentral gyrus<br>Right precentral gyrus	.0013<br>-.0013	1.0513<br>1.1766	.038	140	38	-19	33
Positive Cluster								
1	Left forceps minor<br>Left frontal medial cortex<br>Left frontal Pole	-.0014<br>.0018	1.1432<br>.9703	.049	137	-14	29	-18

Supplementary Table 9. Characteristics of clusters with an interaction between participants' AD-values and error rates. "Negative Cluster" denotes clusters with a lower slope in Parkinson's disease patients compared to HC in the association of participants' AD-values and error rates, whereas "Positive Cluster" denotes clusters with a higher slope in Parkinson's disease patients compared to HC. "Location" indicates the anatomical landmark comprising the majority of voxels of a cluster according to Johns Hopkins University (JHU) white matter atlas, Harvard-Oxford cortical and subcortical atlas, University College London (UCL) cerebellar atlas, and a brainstem atlas based on the DISTAL Atlas of Lead DBS. P-Values are clusterwise p-values corrected for multiple comparisons. "Volume in mm^3" denotes the size of a cluster and

“MNI152-coordinates” describes the coordinates of the cluster’s center of gravity in MNI152-space.

For Review Only

Negative Cluster	Location	Slope (HC, Parkinson's disease)	Intercept (HC, Parkinson's disease)	p-Value	Volume in mm³	MNI152-Coordinates X	Y	Z
1	Left VI	.0031<br>-.0008	.7006<br>.8426	< .001	218	-16	-62	-15
2	Left superior longitudinal fasciculus<br>Left postcentral gyrus<br>Left precentral gyrus	.0011<br>-.0006	.5438<br>.6091	< .001	211	-30	-26	40
3	Left inferior longitudinal fasciculus<br>Left inferior fronto-occipital fasciculus<br>Left inferior temporal gyrus	.0013<br>-.0012	.6238<br>.7389	.019	159	-41	-43	-10
4	Right VI<br>Right crus I	.0019<br>-.0036	.8<br>1.0501	.033	149	37	-57	-20
Positive Cluster								
1	Left superior longitudinal fasciculus	-.0007<br>.0014	.6056<br>.5167	.002	194	-33	3	17

Supplementary Table 10. Characteristics of clusters with an interaction between participants' RD-values and error rates. "Negative Cluster" denotes clusters with a lower slope in Parkinson's disease patients compared to HC in the association of participants' RD-values and error rates, whereas "Positive Cluster" denotes clusters with a higher slope in Parkinson's disease patients compared to HC. "Location" indicates the anatomical landmark comprising the majority of voxels of a cluster according to Johns Hopkins University (JHU) white matter atlas, Harvard-Oxford cortical and subcortical atlas, University College London (UCL) cerebellar atlas, and a brainstem atlas based on the DISTAL Atlas of Lead DBS. P-Values are clusterwise p-values corrected for multiple comparisons. "Volume in mm³" denotes the size of a cluster and "MNI152-coordinates" describes the coordinates of the cluster's center of gravity in MNI152-space.

For Review Only

Positive Cluster	Location	Slope (HC, Parkinson's disease)	Intercept (HC, Parkinson's disease)	p-Value	Volume in mm³	MNI152-Coordinates X	Y	Z
1	Right VI<br>Right crus I	-.002<br>.003	.3837<br>.1934	.002	340	36	-64	-17
2	Left cingulum (cingulate gyrus, anterior division)	-.001<br>.002	.4379<br>.2838	.01	281	-8	12	30
3	Left VI	-.002<br>.001	.4762<br>.3633	.03	249	-17	-61	-16

Supplementary Table 11. Characteristics of clusters with an interaction between participants' ICVF-values and error rates. "Positive Cluster" denotes clusters with a higher slope in Parkinson's disease patients compared to HC in the association of participants' ICVF-values and error rates. "Location" indicates the anatomical landmark comprising the majority of voxels of a cluster according to Johns Hopkins University (JHU) white matter atlas, Harvard-Oxford cortical and subcortical atlas, University College London (UCL) cerebellar atlas, and a brainstem atlas based on the DISTAL Atlas of Lead DBS. P-Values are clusterwise p-values corrected for multiple comparisons. "Volume in mm³" denotes the size of a cluster and "MNI152-coordinates" describes the coordinates of the cluster's center of gravity in MNI152-space.

For Review Only

Positive Cluster	Location	Slope (HC, Parkinson's disease)	Intercept (HC, Parkinson's disease)	p-Value	Volume in mm³	MNI152-Coordinates X	Y	Z
1	Right superior longitudinal fasciculus<br>Right postcentral gyrus<br>Right precentral gyrus	-.001<br>.001	.2264<br>.1406	< .001	473	47	-6	22
2	Left anterior thalamic radiation	-.0006<br>.0009	.2504<br>.1782	< .001	446	-24	19	20
3	Right superior longitudinal fasciculus	-.0008<br>.0005	.276<br>.2117	< .001	352	28	3	31
4	Right VI<br>Right crus I	-.002<br>.0017	.345<br>.1904	< .001	299	36	-63	-18
5	Left superior longitudinal fasciculus<br>Left superior parietal lobule	-.0007<br>.0007	.2197<br>.1428	< .001	293	-36	-48	32
6	Left cingulum (cingulate gyrus, anterior division)	-.0017<br>.0018	.3319<br>.1607	.002	254	-2	14	30
7	Right anterior thalamic radiation<br>Right superior longitudinal fasciculus	-.0013<br>.0009	.2343<br>.1545	.006	231	16	6	27
8	Left VI	-.002<br>.0015	.3508<br>.1337	.006	230	-37	-51	-22
9	Right crus I	-.002	.4467	.018	200	46	-62	-26

		.001	.2873					
10	Left superior longitudinal fasciculus	-.001<br>.0005	.1801<br>.1298	.023	195	-16	10	26
11	Left anterior thalamic radiation<br>Left superior longitudinal fasciculus	-.0003<br>.0007	.2389<br>.1771	.042	182	-21	9	35
12	Right superior longitudinal fasciculus	-.0006<br>.0008	.2132<br>.1275	.042	182	27	-36	29
13	Left VI	-.0014<br>.0007	.366<br>.2843	.045	180	-21	-59	-15

Supplementary Table 12. Characteristics of clusters with an interaction between participants' ODI-values and error rates. "Positive Cluster" denotes clusters with a higher slope in Parkinson's disease patients compared to HC in the association of participants' ODI-values and error rates. "Location" indicates the anatomical landmark comprising the majority of voxels of a cluster according to Johns Hopkins University (JHU) white matter atlas, Harvard-Oxford cortical and subcortical atlas, University College London (UCL) cerebellar atlas, and a brainstem atlas based on the DISTAL Atlas of Lead DBS. P-Values are clusterwise p-values corrected for multiple comparisons. "Volume in mm³" denotes the size of a cluster and "MNI152-coordinates" describes the coordinates of the cluster's center of gravity in MNI152-space.

Review Only

Figure legends

Figure 1. Instructions presented on a screen (left) and demanded button presses (right) in chronological order. Participants learned a sequence for their left hand (here: 1|2|3|4) and tapped a new sequence with their right hand. At the beginning of each trial, the upcoming sequence was presented in red (here: 4|3|2|1) and signalled the subject to prepare for the next trial. The switch from red numbers to green numbers served as "go"-signal, indicating to commence tapping. A red cross, followed by a short break of 5 seconds, marked the end of one trial. In this example, the first requested pair of button presses was left thumb and right ring finger, followed by left index and right middle finger. The third requested pair of button presses was left middle and right index finger followed by left ring finger and right thumb. Participants tapped in synchrony and were instructed to favour correct trial execution over speed. (Figure adapted from Loehrer et al., 2016).

Figure 2. Association between white matter fractional anisotropy in left anterior thalamic radiation and participants' error rates as revealed by GLM. Lower FA-values predicted higher error rates in Parkinson's disease patients, whereas no significant association existed for healthy controls.

Figure 3. Clusters with a lower slope (blue to light blue colour) in the analysis of Parkinson's disease patients' FA-values and error rates in comparison to the association of FA-values and error rates of HC as revealed by the whole brain analysis. P-Values were corrected for multiple comparisons using a permutation-based approach. Results are displayed as the negative decadic logarithm of the p-value ($p=10^{-x}$).

Figure 4. Clusters with a lower (blue to light blue colour) or higher (red to yellow colour) slope in Parkinson's disease patients compared to HC in the association of participants' AD-values and error rates (only lower slopes depicted), as well as RD-values and error rates (only higher slopes depicted) as revealed by the whole brain analysis. P-Values were corrected for multiple comparisons using a permutation-based approach. Results are displayed as the negative decadic logarithm of the p-value ($p=10^{-x}$).

For Review Only

Figure 5. Clusters with a lower (blue to light blue colour) or higher (red to yellow colour) slope in Parkinson's disease patients compared to HC in the association of participants' ICVF-values and error rates as revealed by the whole brain analysis. P-Values were corrected for multiple comparisons using a permutation-based approach. Results are displayed as the negative decadic logarithm of the p-value ($p=10^{-x}$).

Figure 6. Clusters with a lower (blue to light blue colour) or higher (red to yellow colour) slope in Parkinson's disease patients compared to HC in the association of participants' ODI-values and error rates (red to yellow colour) as revealed by the whole brain analysis. P-Values were corrected for multiple comparisons using a permutation-based approach. Results are displayed as the negative decadic logarithm of the p-value ($p=10^{-x}$).

For Review Only

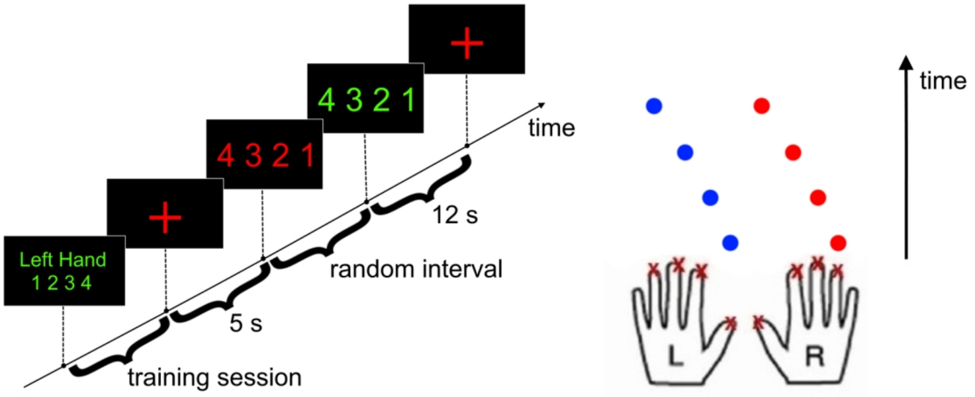

Bimanual Paradigm / Figure 1. Instructions presented on a screen (left) and demanded button presses (right) in chronological order. Participants learned a sequence for their left hand (here: 1|2|3|4) and tapped a new sequence with their right hand. At the beginning of each trial, the upcoming sequence was presented in red (here: 4|3|2|1) and signalled the subject to prepare for the next trial. The switch from red numbers to green numbers served as "go"-signal, indicating to commence tapping. A red cross, followed by a short break of 5 seconds, marked the end of one trial. In this example, the first requested pair of button presses was left thumb and right ring finger, followed by left index and right middle finger. The third requested pair of button presses was left middle and right index finger followed by left ring finger and right thumb. Participants tapped in synchrony and were instructed to favour correct trial execution over speed. (Figure adapted from Loehrer et al., 2016).

139x59mm (300 x 300 DPI)

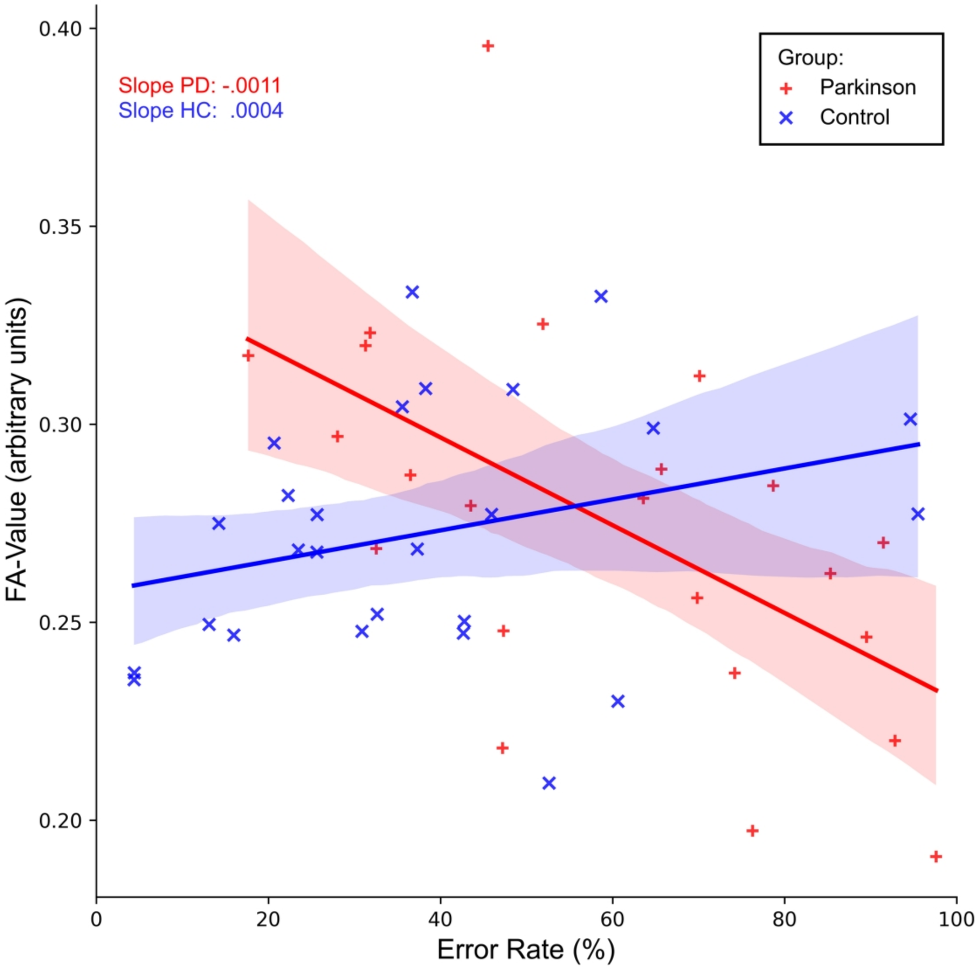

Interaction between FA and bimanual performance in left anterior thalamic radiation / Figure 2. Association between white matter fractional anisotropy in left anterior thalamic radiation and participants' error rates as revealed by GLM. Lower FA-values predicted higher error rates in Parkinson's disease patients, whereas no significant association existed for healthy controls.

178x175mm (300 x 300 DPI)

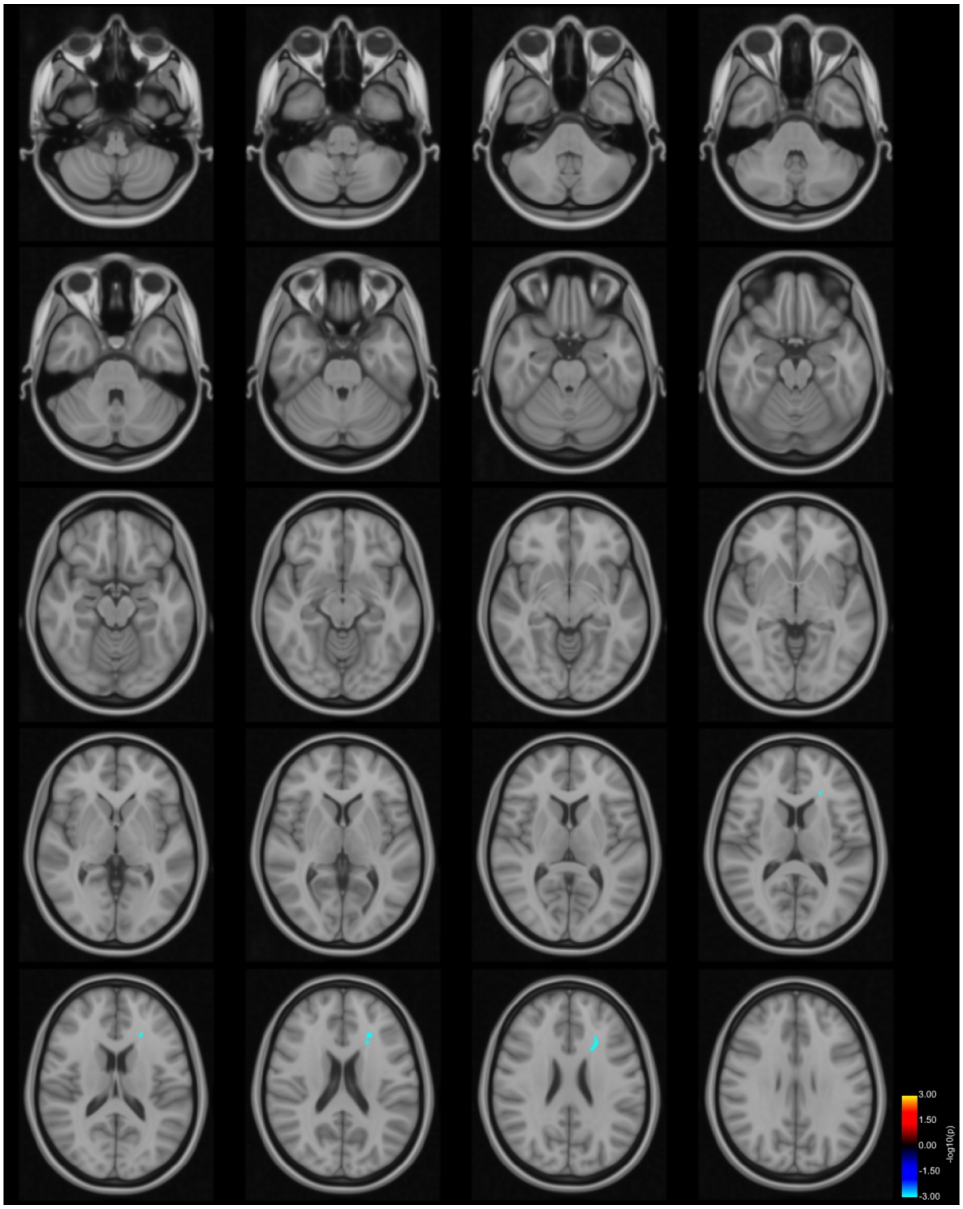

Reduced FA in left anterior thalamic radiation predicts bimanual performance in PD / Figure 3. Clusters with a lower slope (blue to light blue colour) in the analysis of Parkinson's disease patients' FA-values and error rates in comparison to the association of FA-values and error rates of HC as revealed by the whole brain analysis. P-Values were corrected for multiple comparisons using a permutation-based approach. Results are displayed as the negative decadic logarithm of the p-value (p=10-x).

371x466mm (118 x 118 DPI)

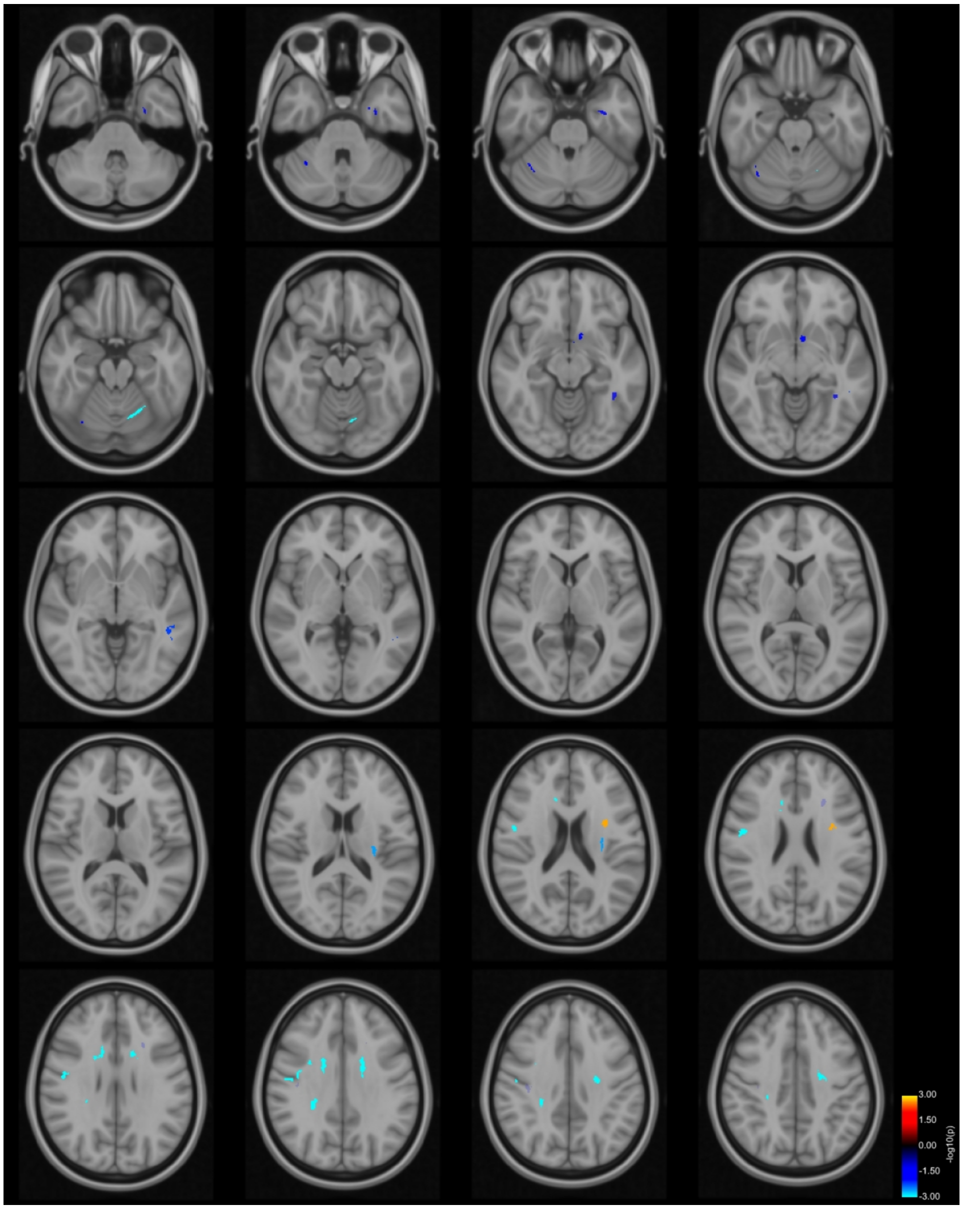

Reduced axial and increased radial diffusivity predicts bimanual performance in PD / Figure 4. Clusters with a lower (blue to light blue colour) or higher (red to yellow colour) slope in Parkinson's disease patients compared to HC in the association of participants' AD-values and error rates (only lower slopes depicted), as well as RD-values and error rates (only higher slopes depicted) as revealed by the whole brain analysis. P-Values were corrected for multiple comparisons using a permutation-based approach. Results are displayed as the negative decadic logarithm of the p-value (p=10-x).

371x466mm (118 x 118 DPI)

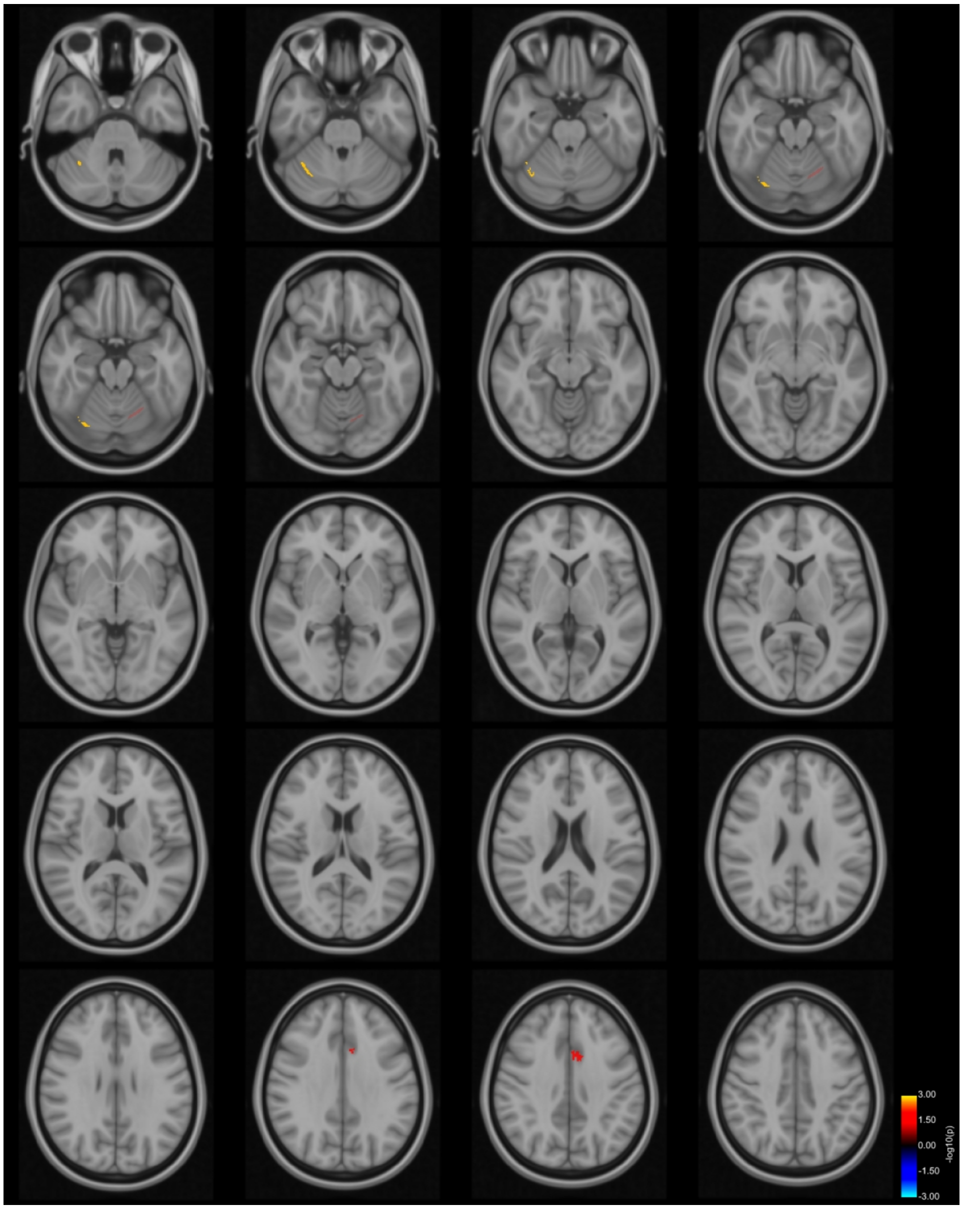

Interaction between intracellular volume fraction and bimanual performance / Figure 5. Clusters with a lower (blue to light blue colour) or higher (red to yellow colour) slope in Parkinson's disease patients compared to HC in the association of participants' ICVF-values and error rates as revealed by the whole brain analysis. P-Values were corrected for multiple comparisons using a permutation-based approach. Results are displayed as the negative decadic logarithm of the p-value (p=10-x).

371x466mm (118 x 118 DPI)

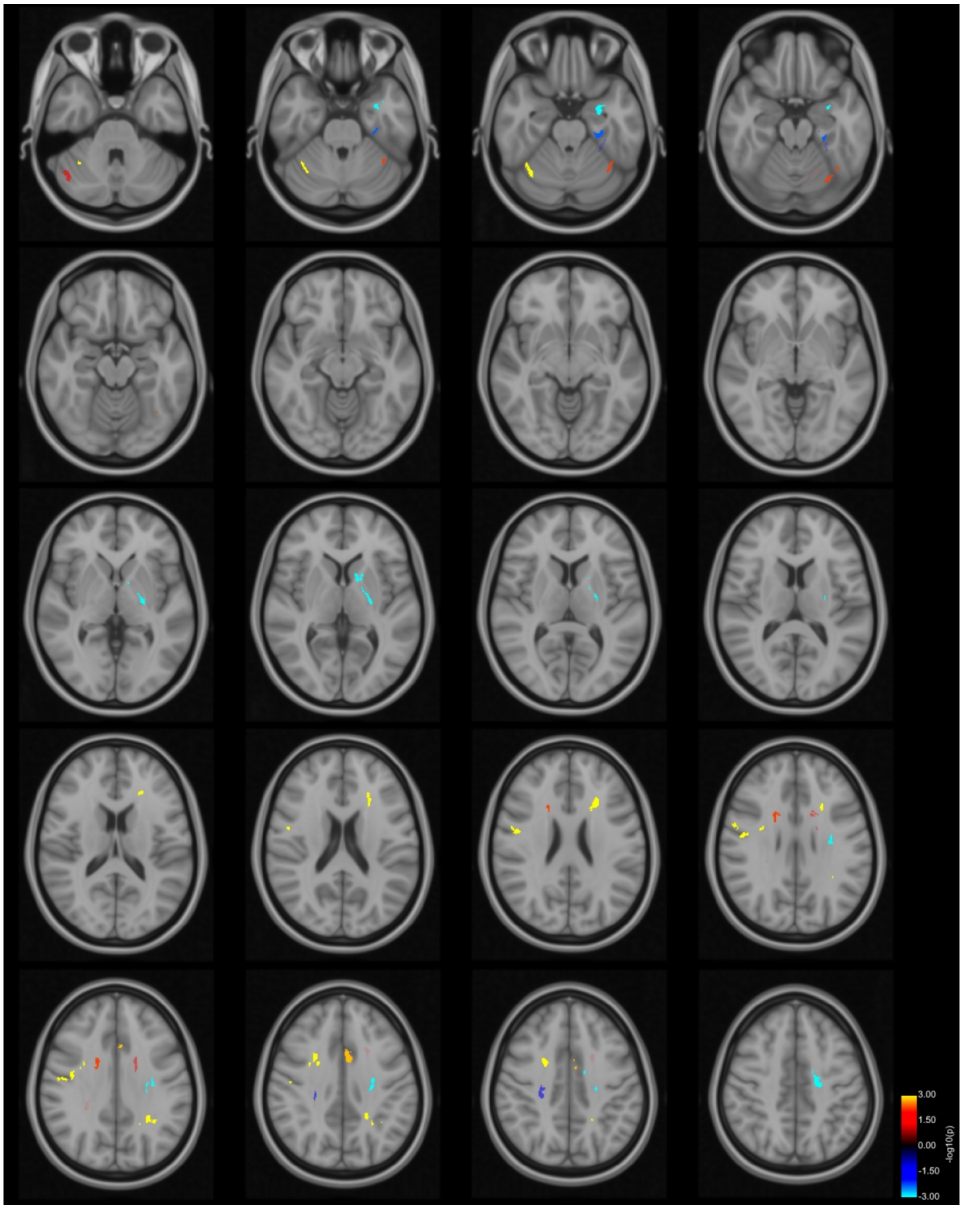

Interaction between orientation dispersion index and bimanual performance / Figure 6. Clusters with a lower (blue to light blue colour) or higher (red to yellow colour) slope in Parkinson's disease patients compared to HC in the association of participants' ODI-values and error rates (red to yellow colour) as revealed by the whole brain analysis. P-Values were corrected for multiple comparisons using a permutation-based approach. Results are displayed as the negative decadic logarithm of the p-value (p=10-x).

371x466mm (118 x 118 DPI)

Tables

Sociodemographic information of Parkinson's disease patients

Age (years)	Gender	Hoehn & Yahr Stage	UPDRS Part III OFF	UPDRS Part III ON	LEDD (mg)	Disease duration	Predominantly affected side
52	M	2	34	23	870	9	right
43	M	2	15	10	500	3	right
54	M	2	32	19	1298	7	left
51	M	2.5	19	4	1195	4	left
46	M	2	25	21	719,25	1	left
64	F	2	22	4	562	8	right
48	F	2	17	7	395	3	right
63	F	3	29	15	1025	9	right
64	F	2	20	7	320	6	right
60	F	2	29	20	630	7	right
58	M	2	31	11	710	7	right
49	M	2	41	18	610	8	right
57	F	2	22	10	300	2	right
61	M	2	14	5	262	3	right
64	M	2	10	4	420	6	right
65	M	2	20	10	297	3	right
50	M	2	34	26	100	6	right
61	F	2	20	11	1110	6	left
58	F	2	16	9	280	2	left
56	M	2	17	10	715	4	right
49	M	2	18	11	815	2	left
58	M	2.5	19	6	240	1	right
57	M	1	9	2	100	5	right
Mean: 56.0 SD: 6.5	Ratio: F:M 8:15	Median: 2 Range: 1-3	Mean: 22.3 SD: 8.2	Mean: 11.4 SD: 6.8	Mean: 585.8 SD: 345.7	Mean: 4.9 SD: 2.6	Ratio: left:right 6:17

Table 1. Sociodemographic information of Parkinson's disease patients, severity of motor symptoms, medication requirements, disease duration since Parkinson's disease diagnosis, and side predominantly affected by Parkinson's disease symptoms. F = female; LEDD = levodopa equivalent daily dose; M = male; SD = standard deviation; UPDRS = Unified Parkinson's Disease Rating Scale

For Review Only
