# Supplementary material for: Microstructural alterations predict impaired bimanual control in Parkinson’s disease
Source: Brain Commun. 2022 May 22;4(3):fcac137. doi: 10.1093/braincomms/fcac137 (PMC9185383; doi:10.1093/braincomms/fcac137)
Supplement: fcac137_Supplementary_Data [file fcac137_supplementary_data.zip › Supplementary_Material.docx]

# Supplementary Material

| Age (years) | Gender | EHI | MMSE | DemTect | BDI-2 |
| --- | --- | --- | --- | --- | --- |
| 52 | F | 82 | 30 | 18 | 0 |
| 54 | M | 85 | 30 | 18 | 0 |
| 64 | M | 8 | 30 | 18 | 0 |
| 52 | M | 60 | 29 | 18 | 0 |
| 57 | M | 75 | 28 | 18 | 5 |
| 63 | M | 88 | 30 | 18 | 3 |
| 62 | M | 91 | 29 | 18 | 0 |
| 68 | M | 75 | 30 | 18 | 0 |
| 62 | F | 83 | 29 | 15 | 0 |
| 62 | F | 92 | 30 | 18 | 2 |
| 50 | M | 83 | 30 | 18 | 0 |
| 56 | M | 100 | 29 | 17 | 1 |
| 62 | M | 100 | 28 | 18 | 0 |
| 68 | M | 100 | 29 | 17 | 3 |
| 52 | F | 91 | 30 | 18 | 3 |
| 62 | F | 64 | 30 | 15 | 10 |
| 55 | F | 91 | 30 | 18 | 1 |
| 56 | M | 94 | 30 | 18 | 3 |
| 68 | M | 78 | 30 | 18 | 1 |
| 50 | M | 100 | 30 | 18 | 2 |
| 62 | M | 5 | 30 | 16 | 0 |
| 56 | F | 76 | 29 | 18 | 2 |
| 58 | F | 100 | 30 | 18 | 0 |
| 56 | F | 100 | 28 | 18 | 0 |
| 61 | F | 100 | 29 | 18 | 0 |
| 53 | F | 100 | 28 | 17 | 5 |
| Mean:  58.5 | Ratio:  F:M | Mean:  81.6 | Mean:  29.4 | Mean:  17.6 | Mean: 1.6 |
| SD: 5.5 | 11:15 | SD: 24.9 | SD: .8 | SD: .9 | SD: 2.3 |

**Supplementary Table 1.** Sociodemographic data and neuropsychological test scores of healthy controls. BDI-2 = Beck’s Depression Inventory 2; DemTect = Dementia Detection Test; EHI = Edinburgh Handedness Inventory; F = female; M = male; MMSE = Minimental State Examination; SD = standard deviation

| Age (years) | Gender | EHI | MMSE | DemTect | BDI-2 |
| --- | --- | --- | --- | --- | --- |
| 52 | M | 26 | 25 | 15 | 11 |
| 43 | M | 41 | 29 | 18 | 10 |
| 54 | M | 100 | 30 | 15 | 13 |
| 51 | M | 100 | 29 | 18 | 9 |
| 46 | M | 88 | 28 | 18 | 9 |
| 64 | F | 75 | 30 | 18 | 2 |
| 48 | F | 100 | 29 | 18 | 1 |
| 63 | F | 58 | 30 | 17 | 11 |
| 64 | F | 90 | 29 | 18 | 7 |
| 60 | F | 92 | 30 | 17 | 13 |
| 58 | M | 75 | 29 | 18 | 1 |
| 49 | M | 68 | 29 | 14 | 7 |
| 57 | F | 45 | 30 | 17 | 7 |
| 61 | M | 100 | 29 | 16 | 6 |
| 64 | M | 83 | 27 | 18 | 4 |
| 65 | M | 70 | 28 | 15 | 4 |
| 50 | M | 83 | 29 | 16 | 4 |
| 61 | F | 43 | 30 | 16 | 11 |
| 58 | F | 92 | 30 | 18 | 4 |
| 56 | M | 89 | 29 | 18 | 2 |
| 49 | M | 67 | 28 | 17 | 3 |
| 58 | M | 92 | 29 | 14 | 9 |
| 57 | M | 100 | 29 | 18 | 3 |
| Mean:  56.0 | Ratio:  F:M | Mean:  77 | Mean:  28.9 | Mean:  16.8 | Mean: 6.6 |
| SD: 6.5 | 8:15 | SD: 21.8 | SD: 1.2 | SD: 1.4 | SD: 3.9 |

**Supplementary Table 2.** Neuropsychological test scores of Parkinson’s disease patients. BDI-2 = Beck’s Depression Inventory 2; DemTect = Dementia Detection Test; EHI = Edinburgh Handedness Inventory; F = female; M = male; MMSE = Minimental State Examination; SD = standard deviation

| Negative  Cluster | Location | p-Value | Volume in mm³ | MNI152-Coordinates | | |
| --- | --- | --- | --- | --- | --- | --- |
|  |  |  |  | X | Y | Z |
| 1 | Left red nucleus  Left ncl. reticularis polaris  Left substantia nigra | 0.024 | 245 | -6 | -16 | -9 |
| 2 | Right ncl. reticularis polaris  Right red nucleus  Right substantia nigra | 0.031 | 236 | 8 | -15 | -4 |
| Positive  Cluster |  |  |  |  |  |  |
|  |  |  |  |  |  |  |
| 1 | Left corticospinal tract | 0.003 | 310 | -18 | -21 | -5 |
| 2 | Right inferior fronto-occipital fasciculus  Right inferior longitudinal fasciculus | 0.009 | 271 | 27 | -21 | -7 |
| 3 | Left cingulum (hippocampus) | 0.042 | 224 | -25 | -17 | -22 |

**Supplementary Table 3.** Characteristics of clusters differing in FA-values between Parkinson’s disease patients and HC. “Negative Cluster” denotes clusters with reduced FA-values in Parkinson’s disease patients compared to HC, whereas “Positive Cluster” denotes clusters with increased FA-values in Parkinson’s disease patients. “Location” indicates the anatomical landmark comprising the majority of voxels of a cluster according to Johns Hopkins University (JHU) white matter atlas, Harvard-Oxford cortical and subcortical atlas, University College London (UCL) cerebellar atlas, and a brainstem atlas based on the DISTAL Atlas of Lead DBS. P-Values are clusterwise p-values corrected for multiple comparisons. “Volume in mm³” denotes the size of a cluster and “MNI152-coordinates” describes the coordinates of the cluster’s center of gravity in MNI152-space.


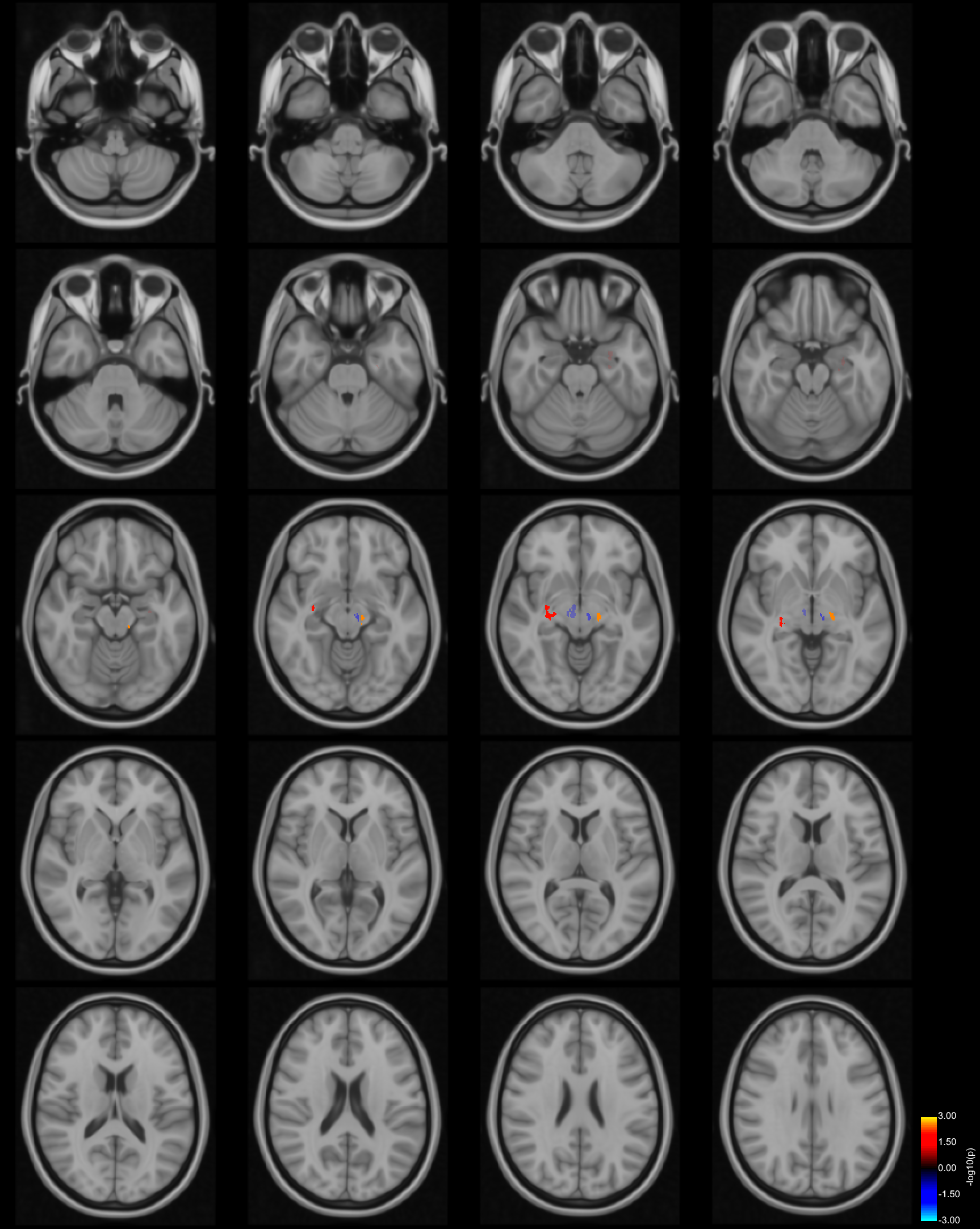


**Supplementary Figure 1.** Clusters differing in fractional anisotropy between Parkinson’s disease patients and HC as revealed by the whole brain analysis. P-Values were corrected for multiple comparisons using a permutation-based approach. Blue to light blue colour indicates clusters with lower FA-values in Parkinson’s disease patients compared to HC. Red to yellow colour indicates clusters with higher FA-values in Parkinson’s disease patients compared to HC. Results are displayed as the negative decadic logarithm of the p-value (p=10^-x^).

| Negative  Cluster | Location | p-Value | Volume in mm³ | MNI152-Coordinates | | |
| --- | --- | --- | --- | --- | --- | --- |
|  |  |  |  | X | Y | Z |
| 1 | Left crus I | < .001 | 795 | -35 | -65 | -22 |
| 2 | Left putamen  Left ncl. accumbens | < .001 | 481 | -15 | 6 | -10 |
| 3 | Forceps Major | < .001 | 373 | 3 | -36 | 11 |
| 4 | Right forceps major  Right precuneous cortex | < .001 | 340 | 16 | -44 | 23 |
| 5 | Right putamen  Right ncl. accumbens | < .001 | 330 | 17 | 8 | -8 |
| 6 | Right crus I | < .001 | 240 | 29 | -67 | -24 |
| 7 | Right inferior fronto-occipital fasciculus | < .001 | 235 | 31 | -34 | 3 |
| 8 | Left IX | < .001 | 216 | -9 | -52 | -25 |
| 9 | Right VI | < .001 | 208 | 17 | -66 | -13 |
| 10 | Left and right I-IV | .001 | 197 | -1 | -58 | -15 |
| 11 | Left forceps minor  Left frontal pole  Left anterior thalamic radiation | .001 | 193 | -22 | 45 | 9 |
| 12 | Left parahippocampal gyrus, anterior division | .015 | 159 | -17 | -12 | -26 |
| 13 | Left Cingulum (cingulate gyrus, anterior division) | .037 | 143 | -7 | -7 | 24 |
| 14 | Right inferior fronto-occipital fasciculus  Right optic tract | .037 | 143 | 15 | -8 | -10 |
| 15 | Right cingulum (cingulate gyrus, posterior division) | .048 | 138 | 13 | -27 | 27 |
| Positive Cluster |  |  |  |  |  |  |
| 1 | Left corticospinal tract | < .001 | 965 | -19 | -13 | 42 |
| 2 | Left Pallidum | < .001 | 451 | -23 | -9 | 3 |
| 3 | Right corticospinal tract | < .001 | 306 | 25 | -27 | 35 |
| 4 | Left Cingulum | < .001 | 217 | -30 | -26 | -19 |
| 5 | Right Pallidum | .048 | 138 | 19 | -7 | 3 |

**Supplementary Table 4.** Characteristics of clusters differing in AD-values between Parkinson’s disease patients and HC. “Negative Cluster” denotes clusters with reduced AD-values in Parkinson’s disease patients compared to HC, whereby “Positive Cluster” denotes clusters with increased AD-values in Parkinson’s disease patients. “Location” indicates the anatomical landmark comprising the majority of voxels of a cluster according to Johns Hopkins University (JHU) white matter atlas, Harvard-Oxford cortical and subcortical atlas, University College London (UCL) cerebellar atlas, and a brainstem atlas based on the DISTAL Atlas of Lead DBS. P-Values are clusterwise p-values corrected for multiple comparisons. “Volume in mm³” denotes the size of a cluster and “MNI152-coordinates” describes the coordinates of the cluster’s center of gravity in MNI152-space.

| Negative  Cluster | Location | p-Value | Volume in mm³ | MNI152-Coordinates | | |
| --- | --- | --- | --- | --- | --- | --- |
|  |  |  |  | X | Y | Z |
| 1 | Left crus I | < .001 | 666 | -35 | -65 | -21 |
| 2 | Left Putamen  Left uncinate fasciculus | < .001 | 369 | -17 | 6 | -11 |
| 3 | Left anterior thalamic radiation | < .001 | 220 | -28 | -28 | 9 |
| 4 | Right forceps major  Right precuneous cortex | < .001 | 220 | 23 | -47 | 22 |
| 5 | Right inferior longitudinal fasciculus  Right corticospinal tract  Right inferior fronto-occipital fasciculus | .002 | 210 | 27 | -22 | -5 |
| 6 | Left IX | .002 | 198 | -9 | -51 | -26 |
| 7 | Right inferior longitudinal fasciculus  Right planum polare | .015 | 164 | 44 | -23 | -9 |
| 8 | Left inferior longitudinal fasciculus  Lateral occipital cortex, superior division | .028 | 153 | -23 | -60 | 32 |
| Positive Cluster |  |  |  |  |  |  |
|  |  |  |  |  |  |  |
| 1 | Left anterior thalamic radiation | .006 | 182 | -3 | -32 | -22 |

**Supplementary Table 5.** Characteristics of clusters differing in RD-values between Parkinson’s disease patients and HC. “Negative Cluster” denotes clusters with reduced RD-values in Parkinson’s disease patients compared to HC, whereby “Positive Cluster” denotes clusters with increased RD-values in Parkinson’s disease patients. “Location” indicates the anatomical landmark comprising the majority of voxels of a cluster according to Johns Hopkins University (JHU) white matter atlas, Harvard-Oxford cortical and subcortical atlas, University College London (UCL) cerebellar atlas, and a brainstem atlas based on the DISTAL Atlas of Lead DBS. P-Values are clusterwise p-values corrected for multiple comparisons. “Volume in mm³” denotes the size of a cluster and “MNI152-coordinates” describes the coordinates of the cluster’s center of gravity in MNI152-space.


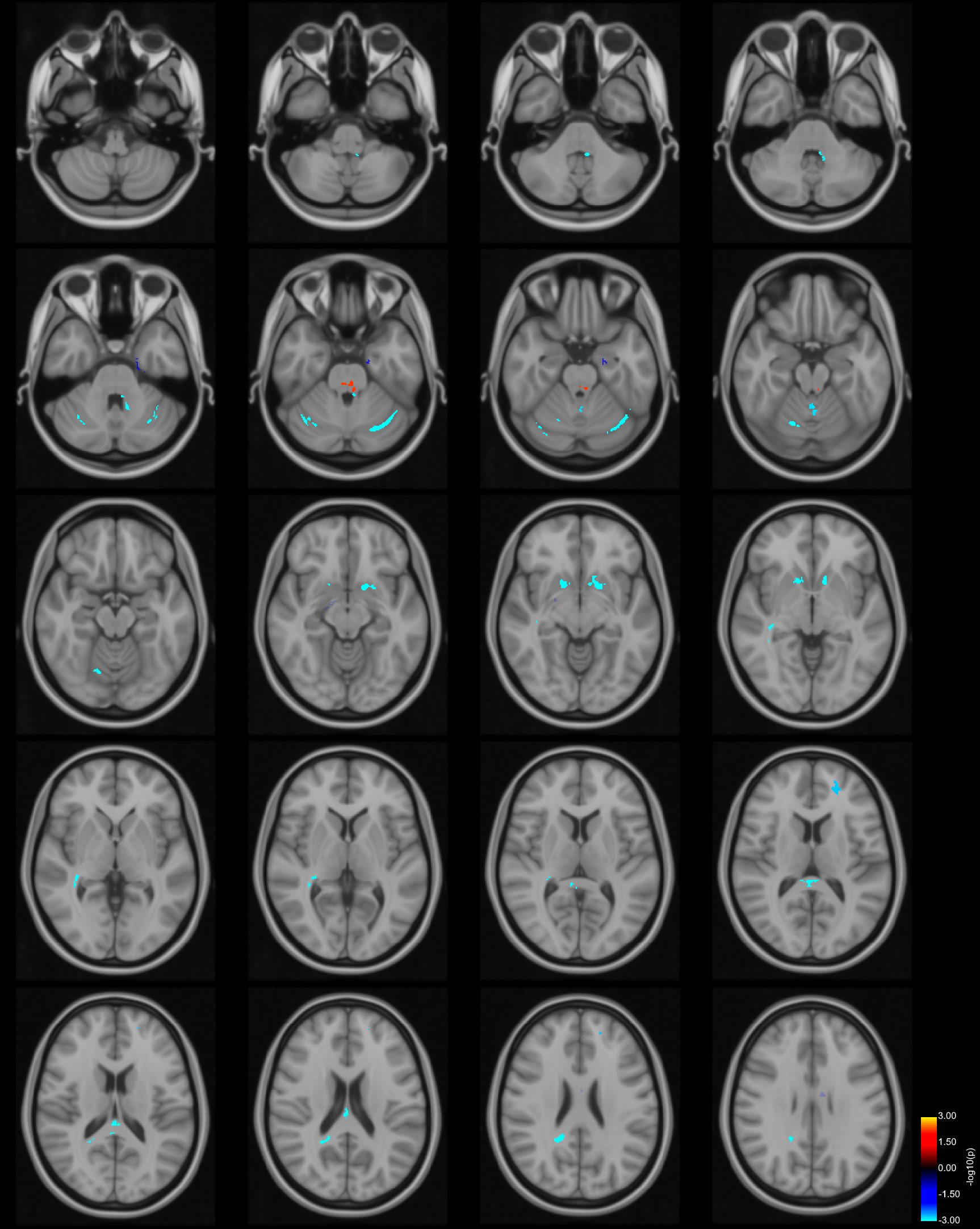


**Supplementary Figure 2.** Clusters differing in axial and radial diffusivity between Parkinson’s disease patients and HC as revealed by the whole brain analysis. P-Values were corrected for multiple comparisons using a permutation-based approach. Blue to light blue colour indicates clusters with lower AD-values in Parkinson’s disease patients compared to HC. Red to yellow colour indicates clusters with higher RD-values in Parkinson’s disease compared to HC patients. Results are displayed as the negative decadic logarithm of the p-value (p=10^-x^).

| Positive  Cluster | Location | p-Value | Volume in mm³ | MNI152-Coordinates | | |
| --- | --- | --- | --- | --- | --- | --- |
|  |  |  |  | X | Y | Z |
| 1 | Left crus I | < .001 | 798 | -35 | -65 | -21 |
| 2 | Left putamen  Left ncl. accumbens | < .001 | 658 | -17 | 6 | -10 |
| 3 | Right forceps major  Right precuneous cortex | < .001 | 515 | 22 | -49 | 15 |
| 4 | Right inferior fronto-occipital fasciculus  Right inferior longitudinal fasciculus | < .001 | 507 | 31 | -33 | 4 |
| 5 | Forceps Major | < .001 | 448 | 3 | -36 | 10 |
| 6 | Left frontal pole  Left anterior thalamic radiation  Left forceps minor | .004 | 316 | -19 | 41 | 20 |
| 7 | Right putamen  Right anterior thalamic radiation | < .009 | 296 | 15 | 5 | -9 |
| 8 | Right crus I | .01 | 282 | 31 | -65 | -23 |
| 9 | Right VI | .02 | 270 | 29 | -58 | -16 |

**Supplementary Table 6.** Characteristics of clusters differing in ICVF-values between Parkinson’s disease patients and HC. “Positive Cluster” denotes clusters with increased ICVF-values in Parkinson’s disease patients. “Location” indicates the anatomical landmark comprising the majority of voxels of a cluster according to Johns Hopkins University (JHU) white matter atlas, Harvard-Oxford cortical and subcortical atlas, University College London (UCL) cerebellar atlas, and a brainstem atlas based on the DISTAL Atlas of Lead DBS. P-Values are clusterwise p-values corrected for multiple comparisons. “Volume in mm³” denotes the size of a cluster and “MNI152-coordinates” describes the coordinates of the cluster’s center of gravity in MNI152-space.

| Negative  Cluster | Location | p-Value | Volume in mm³ | MNI152-Coordinates | | |
| --- | --- | --- | --- | --- | --- | --- |
|  |  |  |  | X | Y | Z |
| 1 | Left corticospinal tract Left superior longitudinal fasciculus | < .001 | 963 | -19 | -14 | 42 |
| 2 | Left pallidum  Left anterior thalamic radiation | < .001 | 450 | -13 | 5 | 3 |
| 3 | Left parahippocampal gyrus, anterior division Left Amygdala | < .001 | 305 | -26 | -6 | -20 |
| 4 | Left parahippocampal gyrus, posterior division | .004 | 245 | -28 | -26 | -21 |
| 5 | Right corticospinal tract | .02 | 202 | 25 | -19 | 36 |
| 6 | Left temporal fusiform cortex, posterior division | .04 | 186 | -28 | -36 | -17 |
| Positive Cluster |  |  |  |  |  |  |
|  |  |  |  |  |  |  |
| 1 | Left crus I Left VI | < .001 | 785 | -30 | -68 | -22 |
| 2 | Left and right I-IV | .001 | 478 | 3 | -53 | -16 |
| 3 | Left putamen  Left ncl. accumbens | < .001 | 427 | -12 | 12 | -6 |
| 4 | Right putamen  Right ncl. Accumbens Right anterior thalamic radiation | < .001 | 401 | 13 | 10 | -6 |
| 5 | Right inferior fronto-occipital fasciculus | < .001 | 310 | 39 | -28 | -4 |
| 6 | Right crus I | .002 | 260 | 42 | -59 | -19 |
| 7 | Right precuneous cortex  Right forceps major | .002 | 250 | 16 | -44 | 23 |
| 8 | Right insular cortex | .004 | 239 | 40 | -8 | 7 |
| 9 | Left anterior thalamic radiation Left Thalamus | .02 | 201 | -3 | -2 | -7 |
| 10 | Right intracalcarine cortex Right lingual gyrus | .02 | 195 | 10 | -69 | 7 |
| 11 | Right VI | .04 | 184 | 18 | -67 | -14 |
| 12 | Left frontal pole  Left forceps minor  Left anterior thalamic radiation | .04 | 183 | -21 | 51 | 10 |

**Supplementary Table 7.** Characteristics of clusters differing in ODI-values between Parkinson’s disease patients and HC. “Negative Cluster” denotes clusters with reduced ODI-values in Parkinson’s disease patients compared to HC, whereby “Positive Cluster” denotes clusters with increased ODI-values in Parkinson’s disease patients. “Location” indicates the anatomical landmark comprising the majority of voxels of a cluster according to Johns Hopkins University (JHU) white matter atlas, Harvard-Oxford cortical and subcortical atlas, University College London (UCL) cerebellar atlas, and a brainstem atlas based on the DISTAL Atlas of Lead DBS. P-Values are clusterwise p-values corrected for multiple comparisons. “Volume in mm³” denotes the size of a cluster and “MNI152-coordinates” describes the coordinates of the cluster’s center of gravity in MNI152-space.


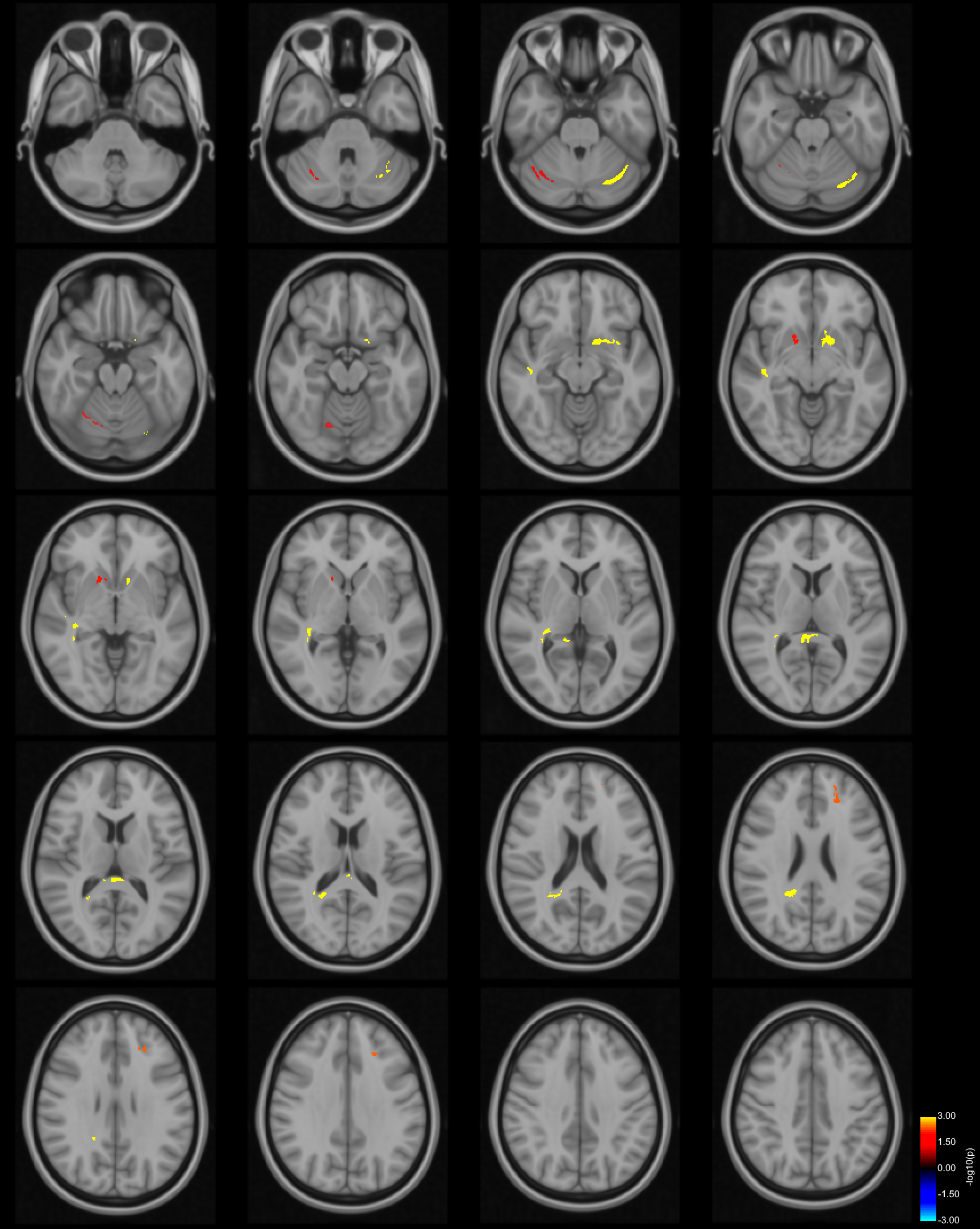


**Supplementary Figure 3.** Clusters differing in ICVF between Parkinson’s disease patients and HC as revealed by the whole brain analysis. P-Values were corrected for multiple comparisons using a permutation-based approach. Red to yellow colour indicates clusters with higher ICVF-values in Parkinson’s disease patients compared to HC. Results are displayed as the negative decadic logarithm of the p-value (p=10^-x^).

**
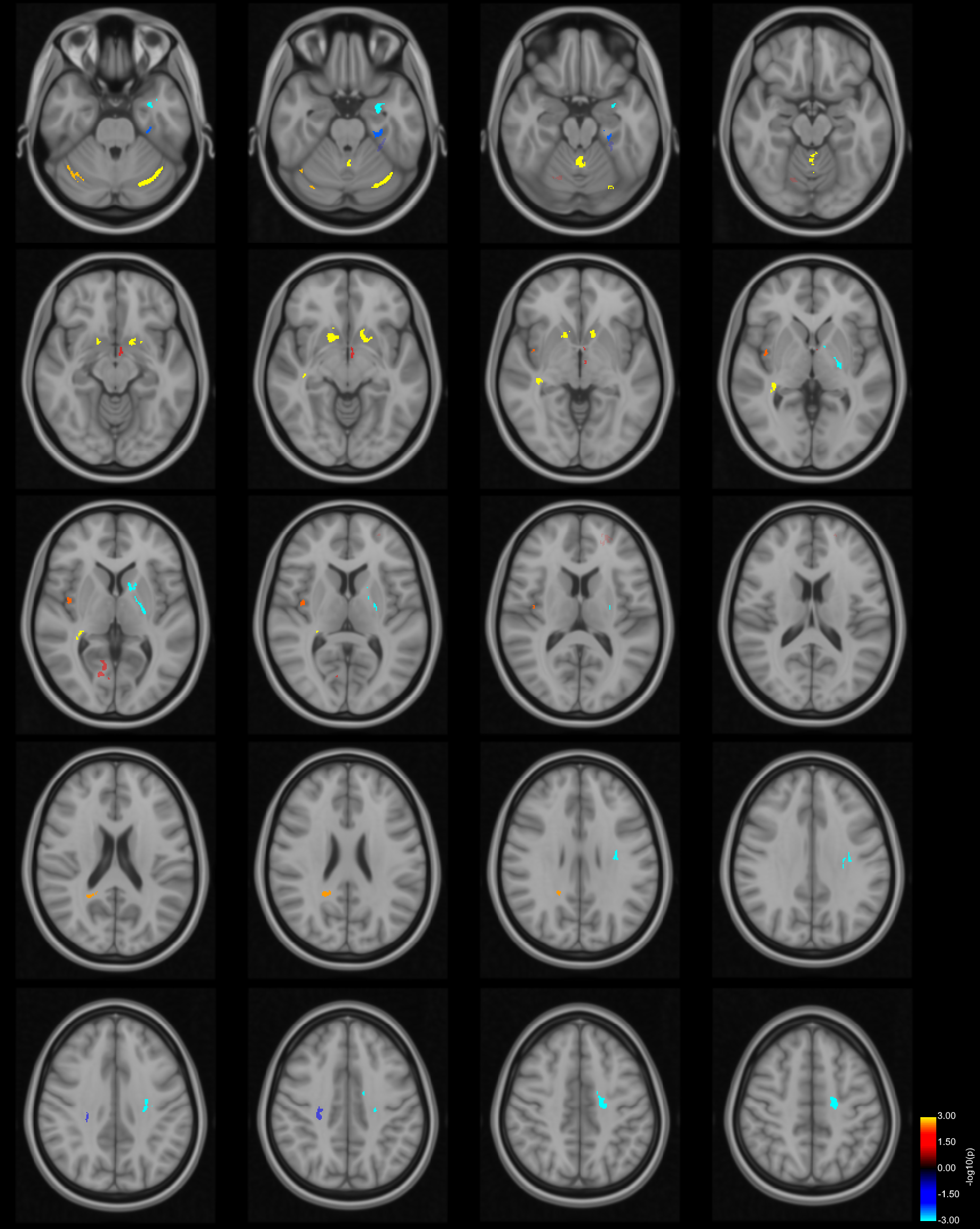
**

**Supplementary Figure 4.** Clusters differing in ODI between Parkinson’s disease patients and HC as revealed by the whole brain analysis. P-Values were corrected for multiple comparisons using a permutation-based approach. Red to yellow colour indicates clusters with higher ODI-values in Parkinson’s disease compared to HC patients. Blue to light blue colour indicates clusters with lower ODI-values in Parkinson’s disease patients compared to HC. Results are displayed as the negative decadic logarithm of the p-value (p=10^-x^).

| Negative Cluster | Location | Slope  (HC, PD) | Intercept (HC, PD) | p-Value | Volume in mm³ | MNI152-Coordinates | | |
| --- | --- | --- | --- | --- | --- | --- | --- | --- |
|  |  |  |  |  |  | X | Y | Z |
| 1 | Left anterior thalamic radiation | .0004 -.0011 | .2576 .341 | < .001 | 386 | -22 | 28 | 11 |

**Supplementary Table 8.** Characteristics of the cluster with an interaction between participants’ FA-values and error rates. “Negative Cluster” denotes clusters with a lower slope in Parkinson’s disease patients compared to HC in the association of participants’ FA-values and error rates, i.e. lower FA-values predicted higher error rates. “Location” indicates the anatomical landmark comprising the majority of voxels of a cluster according to Johns Hopkins University (JHU) white matter atlas, Harvard-Oxford cortical and subcortical atlas, University College London (UCL) cerebellar atlas, and a brainstem atlas based on the DISTAL Atlas of Lead DBS. P-Values are clusterwise p-values corrected for multiple comparisons. “Volume in mm³” denotes the size of a cluster and “MNI152-Coordinates” describes the coordinates of the cluster’s center of gravity in MNI152-space.

| Negative Cluster | Location | Slope  (HC,  PD) | Intercept (HC,  PD) | p- Value | Volume in mm³ | MNI152-Coordinates | | |
| --- | --- | --- | --- | --- | --- | --- | --- | --- |
|  |  |  |  |  |  | X | Y | Z |
| 1 | Right superior longitudinal fasciculus  Right postcentral gyrus  Right precentral gyrus | .0019 | 1.0186 | < .001 | 523 | 49 | -7 | 23 |
|  |  | -.0011 | 1.1395 |  |  |  |  |  |
| 2 | Right superior longitudinal fasciculus  Right corticospinal tract | .0012 | 1.015 | < .001 | 310 | 27 | -36 | 29 |
|  |  | -.0011 | 1.1587 |  |  |  |  |  |
| 3 | Right cingulum (cingulate gyrus, anterior division)  Forceps minor | .0021 | 1.2089 | < .001 | 300 | 14 | 8 | 26 |
|  |  | -.0012 | 1.331 |  |  |  |  |  |
| 4 | Left VI | .003 | .93 | < .001 | 265 | -21 | -59 | -16 |
|  |  | -.0011 | 1.0802 |  |  |  |  |  |
| 5 | Left cingulum (cingulate gyrus, anterior division)  Forceps minor  Left superior longitudinal fasciculus | .0025 | 1.1592 | < .001 | 264 | -17 | -2 | 28 |
|  |  | -.0011 | 1.2715 |  |  |  |  |  |
| 6 | Left corticospinal tract  Left superior longitudinal fasciculus  Left precentral gyrus | .0012 | .9034 | < .001 | 234 | -24 | -10 | 32 |
|  |  | -.0007 | 1.0072 |  |  |  |  |  |
| 7 | Left corticospinal tract  Left superior longitudinal fasciculus  Left insular cortex | .0012 | 1.2084 | < .001 | 201 | -29 | -22 | 16 |
|  |  | -.001 | 1.3106 |  |  |  |  |  |
| 8 | Left superior longitudinal fasciculus  Left middle temporal gyrus | .0013 | 1.0582 | < .001 | 177 | -50 | -37 | -4 |
|  |  | -.0018 | 1.1933 |  |  |  |  |  |
| 9 | Left ncl. accumbens  Left uncinate fasciculus | .002 | .8757 | < .001 | 166 | -8 | 5 | -11 |
|  |  | -.0012 | .9955 |  |  |  |  |  |
| 10 | Right VI  Right crus I | .0025 | 1.0054 | .01 | 165 | 37 | -57 | -20 |
|  |  | -.0035 | 1.2659 |  |  |  |  |  |
| 11 | Left Cingulum (hippocampus)  Left parahippocampal gyrus, anterior division | .0053 | .8349 | .012 | 160 | -30 | -9 | -25 |
|  |  | -.0003 | 1.0223 |  |  |  |  |  |
| 12 | Left inferior longitudinal fasciculus  Left inferior fronto-occipital fasciculus  Left temporal occipital fusiform cortex | .0018 | .9678 | .015 | 158 | -40 | -47 | -9 |
|  |  | -.0019 | 1.158 |  |  |  |  |  |
| 13 | Left anterior thalamic radiation | .0012 | .9913 | .037 | 141 | -24 | 19 | 20 |
|  |  | -.0013 | 1.1168 |  |  |  |  |  |
| 14 | Right superior longitudinal fasciculus  Right postcentral gyrus  Right precentral gyrus | .0013 | 1.0513 | .038 | 140 | 38 | -19 | 33 |
|  |  | -.0013 | 1.1766 |  |  |  |  |  |
| Positive Cluster |  |  |  |  |  |  |  |  |
|  |  |  |  |  |  |  |  |  |
| 1 | Left forceps minor  Left frontal medial cortex  Left frontal Pole | -.0014 | 1.1432 | .049 | 137 | -14 | 29 | -18 |
|  |  | .0018 | .9703 |  |  |  |  |  |

**Supplementary Table 9.** Characteristics of clusters with an interaction between participants’ AD-values and error rates. “Negative Cluster” denotes clusters with a lower slope in Parkinson’s disease patients compared to HC in the association of participants’ AD-values and error rates, i.e. lower AD-values predicted higher error rates. “Positive Cluster” denotes clusters with a higher slope in Parkinson’s disease patients compared to HC, i.e. higher AD-values predicted higher error rates. “Location” indicates the anatomical landmark comprising the majority of voxels of a cluster according to Johns Hopkins University (JHU) white matter atlas, Harvard-Oxford cortical and subcortical atlas, University College London (UCL) cerebellar atlas, and a brainstem atlas based on the DISTAL Atlas of Lead DBS. P-Values are clusterwise p-values corrected for multiple comparisons. “Volume in mm³” denotes the size of a cluster and “MNI152-coordinates” describes the coordinates of the cluster’s center of gravity in MNI152-space.

| Negative  Cluster | Location | Slope (HC, PD) | Intercept (HC, PD) | p- Value | Volume in mm³ | MNI152- Coordinates | | |
| --- | --- | --- | --- | --- | --- | --- | --- | --- |
|  |  |  |  |  |  | X | Y | Z |
| 1 | Left VI | .0031 | .7006 | < .001 | 218 | -16 | -62 | -15 |
|  |  | -.0008 | .8426 |  |  |  |  |  |
| 2 | Left superior longitudinal fasciculus  Left postcentral gyrus  Left precentral gyrus | .0011 | .5438 | < .001 | 211 | -30 | -26 | 40 |
|  |  | -.0006 | .6091 |  |  |  |  |  |
| 3 | Left inferior longitudinal fasciculus  Left inferior fronto-occipital fasciculus  Left inferior temporal gyrus | .0013 | .6238 | .019 | 159 | -41 | -43 | -10 |
|  |  | -.0012 | .7389 |  |  |  |  |  |
| 4 | Right VI  Right crus I | .0019 | .8 | .033 | 149 | 37 | -57 | -20 |
|  |  | -.0036 | 1.0501 |  |  |  |  |  |
| Positive Cluster |  |  |  |  |  |  |  |  |
|  |  |  |  |  |  |  |  |  |
| 1 | Left superior longitudinal fasciculus | -.0007 | .6056 | .002 | 194 | -33 | 3 | 17 |
|  |  | .0014 | .5167 |  |  |  |  |  |

**Supplementary Table 10.** Characteristics of clusters with an interaction between participants’ RD-values and error rates. “Negative Cluster” denotes clusters with a lower slope in Parkinson’s disease patients compared to HC in the association of participants’ RD-values and error rates, i.e. lower RD-values predicted higher error rates. “Positive Cluster” denotes clusters with a higher slope in Parkinson’s disease patients compared to HC, i.e. higher RD-values predicted higher error rates. “Location” indicates the anatomical landmark comprising the majority of voxels of a cluster according to Johns Hopkins University (JHU) white matter atlas, Harvard-Oxford cortical and subcortical atlas, University College London (UCL) cerebellar atlas, and a brainstem atlas based on the DISTAL Atlas of Lead DBS. P-Values are clusterwise p-values corrected for multiple comparisons. “Volume in mm³” denotes the size of a cluster and “MNI152-coordinates” describes the coordinates of the cluster’s center of gravity in MNI152-space.

| Positive Cluster | Location | Slope  (HC,  PD) | Intercept (HC,  PD) | p- Value | Volume in mm³ | MNI152-Coordinates | | |
| --- | --- | --- | --- | --- | --- | --- | --- | --- |
|  |  |  |  |  |  | X | Y | Z |
| 1 | Right VI  Right crus I | -.002 | .3837 | .002 | 340 | 36 | -64 | -17 |
|  |  | .003 | .1934 |  |  |  |  |  |
| 2 | Left cingulum (cingulate gyrus, anterior division) | -.001 | .4379 | .01 | 281 | -8 | 12 | 30 |
|  |  | .002 | .2838 |  |  |  |  |  |
| 3 | Left VI | -.002 | .4762 | .03 | 249 | -17 | -61 | -16 |
|  |  | .001 | .3633 |  |  |  |  |  |

**Supplementary Table 11.** Characteristics of clusters with an interaction between participants’ ICVF-values and error rates. “Positive Cluster” denotes clusters with a higher slope in Parkinson’s disease patients compared to HC in the association of participants’ ICVF-values and error rates, i.e. higher ICVF-values predicted higher error rates. “Location” indicates the anatomical landmark comprising the majority of voxels of a cluster according to Johns Hopkins University (JHU) white matter atlas, Harvard-Oxford cortical and subcortical atlas, University College London (UCL) cerebellar atlas, and a brainstem atlas based on the DISTAL Atlas of Lead DBS. P-Values are clusterwise p-values corrected for multiple comparisons. “Volume in mm³” denotes the size of a cluster and “MNI152-coordinates” describes the coordinates of the cluster’s center of gravity in MNI152-space.

| Positive Cluster | Location | Slope  (HC,  PD) | Intercept (HC,  PD) | p- Value | Volume in mm³ | MNI152-Coordinates | | |
| --- | --- | --- | --- | --- | --- | --- | --- | --- |
|  |  |  |  |  |  | X | Y | Z |
| 1 | Right superior longitudinal fasciculus  Right postcentral gyrus  Right precentral gyrus | -.001 | .2264 | < .001 | 473 | 47 | -6 | 22 |
|  |  | .001 | .1406 |  |  |  |  |  |
| 2 | Left anterior thalamic radiation | -.0006 | .2504 | < .001 | 446 | -24 | 19 | 20 |
|  |  | .0009 | .1782 |  |  |  |  |  |
| 3 | Right superior longitudinal fasciculus | -.0008 | .276 | < .001 | 352 | 28 | 3 | 31 |
|  |  | .0005 | .2117 |  |  |  |  |  |
| 4 | Right VI  Right crus I | -.002 | .345 | < .001 | 299 | 36 | -63 | -18 |
|  |  | .0017 | .1904 |  |  |  |  |  |
| 5 | Left superior longitudinal fasciculus  Left superior parietal lobule | -.0007 | .2197 | < .001 | 293 | -36 | -48 | 32 |
|  |  | .0007 | .1428 |  |  |  |  |  |
| 6 | Left cingulum (cingulate gyrus, anterior division) | -.0017 | .3319 | .002 | 254 | -2 | 14 | 30 |
|  |  | .0018 | .1607 |  |  |  |  |  |
| 7 | Right anterior thalamic radiation Right superior longitudinal fasciculus | -.0013 | .2343 | .006 | 231 | 16 | 6 | 27 |
|  |  | .0009 | .1545 |  |  |  |  |  |
| 8 | Left VI | -.002 | .3508 | .006 | 230 | -37 | -51 | -22 |
|  |  | .0015 | .1337 |  |  |  |  |  |
| 9 | Right crus I | -.002 | .4467 | .018 | 200 | 46 | -62 | -26 |
|  |  | .001 | .2873 |  |  |  |  |  |
| 10 | Left superior longitudinal fasciculus | -.001 | .1801 | .023 | 195 | -16 | 10 | 26 |
|  |  | .0005 | .1298 |  |  |  |  |  |
| 11 | Left anterior thalamic radiation Left superior longitudinal fasciculus | -.0003 | .2389 | .042 | 182 | -21 | 9 | 35 |
|  |  | .0007 | .1771 |  |  |  |  |  |
| 12 | Right superior longitudinal fasciculus | -.0006 | .2132 | .042 | 182 | 27 | -36 | 29 |
|  |  | .0008 | .1275 |  |  |  |  |  |
| 13 | Left VI | -.0014 | .366 | .045 | 180 | -21 | -59 | -15 |
|  |  | .0007 | .2843 |  |  |  |  |  |

**Supplementary Table 12.** Characteristics of clusters with an interaction between participants’ ODI-values and error rates. “Positive Cluster” denotes clusters with a higher slope in Parkinson’s disease patients compared to HC in the association of participants’ ODI-values and error rates, i.e. higher ODI-values predicted higher error rates. “Location” indicates the anatomical landmark comprising the majority of voxels of a cluster according to Johns Hopkins University (JHU) white matter atlas, Harvard-Oxford cortical and subcortical atlas, University College London (UCL) cerebellar atlas, and a brainstem atlas based on the DISTAL Atlas of Lead DBS. P-Values are clusterwise p-values corrected for multiple comparisons. “Volume in mm³” denotes the size of a cluster and “MNI152-coordinates” describes the coordinates of the cluster’s center of gravity in MNI152-space.
