# Supplementary material for: Microstructural alterations predict impaired bimanual control in Parkinson’s disease
Source: Brain Commun. 2022 May 22;4(3):fcac137. doi: 10.1093/braincomms/fcac137 (PMC9185383; doi:10.1093/braincomms/fcac137)
Supplement: fcac137_Supplementary_Data [file BRAINCOM-2022-033.R1 Decision letter attachment.pdf › BRAINCOM-2022-033.R1 Decision letter attachment.pdf]

In this study Loehrer and coauthors investigate whether microstructural abnormalities in PD are
associated with the performance of a complex task measuring bimanual control and coordination.
The question is relevant to the pathophysiology of motor impairment in PD. The task employed in
this study has been used and previously validated and was performed in on and off states.
Microstructure was investigated through diffusion MRI and the application of DTI and DTI-
NODDI models. Several associations between altered microstructure (in cerebellar, midbrain,
cortical areas) and reduced performance in the bimanual control task are found.
Although the results are sometimes difficult to parse in text, figures and supplementary tables, the
main issues are clearly analyzed in the discussion.
To this reviewer the following points should be clarified:

- In the statistical analysis section it is stated: “A non-Gaussian distribution of error rates was
revealed by the Shapiro-Wilk test...”. It is not clear whether the square root of error rates
obtained a normal distribution, and whether all assumptions of ANOVA were fulfilled.
- Statistical analysis section: “Voxelwise cross-subject statistics were carried out as described
previously,34 employing FreeSurfer. The data was fit into a generalized linear model, and
an unpaired t-test was performed.”
  - Voxelwise statistics seem critical enough to this study that a brief explanation is
needed on top of reference 34.
  - It seems like comparisons in DTI metrics between PD and HC were assessed through
a generalized linear model. Howerver, the first sentence of paragraph 3.2.1 states
otherwise (“To assess differences in microstructure between Parkinson’s disease
patients and HC we employed a general linear model (GLM).”)
  - The authors used a generalized linear model (?) to compare diffusion metrics

between PD and HC. However, methodological information about voxelwise association analysis with clinical measures does not seem to be available throughout the paper (except for a brief sentence at the beginning of paragraph 3.2.4).

- In regard to the previous comment, it seems like a GLM was fit to DTI data and error rates from all participants (PD and HC). A p-value for each cluster is given and the slopes for HC and PD are provided. Figures 2 and 3 show significant differences in slopes between PD and HC. Would there be a way to provide such data altogether, e.g. also in the supplementary tables (8-12)?

• As the authors point out in the discussion, microstructural alterations are now always easy to interpret, especially in the setting of pathology; this is made even more difficult by the fact that PD is not a condition prominently and primarily involving white matter.

- Could the author briefly comment on diffusion metrics findings between patients and controls? Are these in agreement with previous studies employing similar acquisition and processing techniques? Although the literature has provided conflicting results, a comment on this could improve interpretability by a larger readership.
- Sometimes clusters involve grey and white matter areas. An explanation about the interpretation of the results in these clusters would allow more readers to understand the findings.

• In the discussion section the authors state: “Concluding, evidence exists that microstructural alterations in ILF and IFOF impairs visual information processing which contributes to the evolvement of specific clinical symptoms and can be associated to impaired behavioural measures. In the context of Parkinson’s disease, our results suggest that compromised microstructure in both fiber tracts can be a contributing factor to reduced bimanual coordination.”

- Since the task performed involved learning a tapping sequence from a computer screen, would it be possible that disruption of visual association pathways were the cause driving impaired bimanual control? Could the authors speculate about this result in the setting of a “bimanual control task” without visual instruction?
